# Structure of [18]Annulene Revisited: Challenges for Computing Benzenoid Systems

**DOI:** 10.1021/acs.jpca.3c07797

**Published:** 2024-02-02

**Authors:** Rollin A. King, Peter R. Schreiner, T. Daniel Crawford

**Affiliations:** † Department of Chemistry, Bethel University, St. Paul, Minnesota 55112, United States; ‡ Institute of Organic Chemistry, Justus Liebig University, Heinrich-Buff-Ring 17, Giessen 35392, Germany; § Department of Chemistry, Virginia Tech, Blacksburg, Virginia 24061, United States

## Abstract

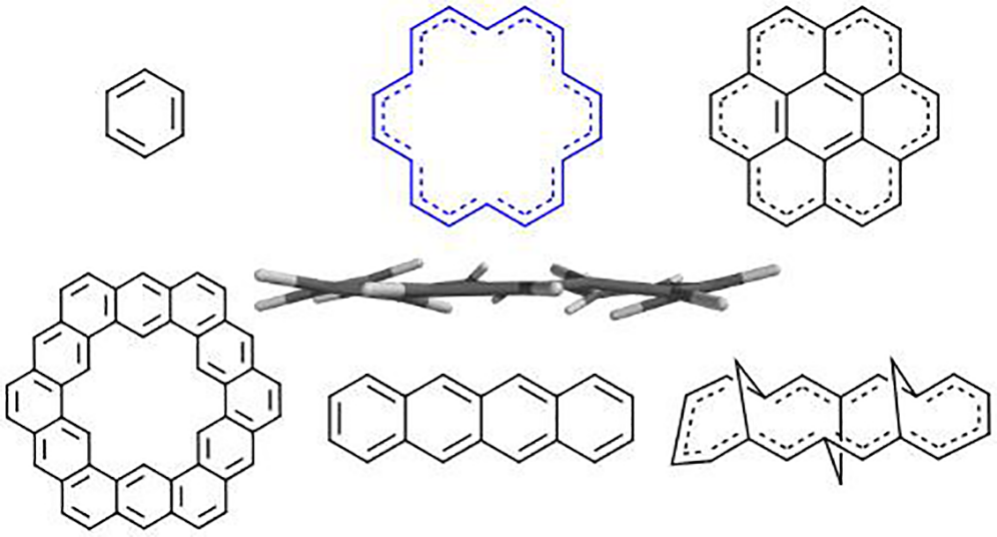

For cyclic conjugated structures, erratic computational results have been obtained with Hartree–Fock (HF) molecular orbital (MO) methods as well as density functional theory (DFT) with large HF-exchange contributions. In this work, the reasons for this unreliability are explored. Extensive computations on [18]annulene and related compounds highlight the pitfalls to be avoided and the due diligence required for such computational investigations. In particular, a careful examination of the MO singlet-stability eigenvalues is recommended. The appearance of negative eigenvalues is not (necessarily) problematic, but near-zero (positive or negative) eigenvalues can lead to dramatic errors in vibrational frequencies and related properties. DFT approaches with a lower HF admixture generally appear more robust in this regard for the description of benzenoid structures, although they may exaggerate the tendency toward planarity and C–C bond-equalization. For the iconic [18]annulene, the results support a nonplanar equilibrium structure. The density-fitted frozen natural orbital coupled-cluster singles and doubles with perturbative triples [DF-FNO CCSD(T)] method of electron correlation with an aug-pVQZ/aug-pVTZ basis set places the *C*_2_ global minimum 1.1 kcal mol^–1^ below the *D*_6*h*_ stationary point.

## 1 Introduction

**Figure 1:**
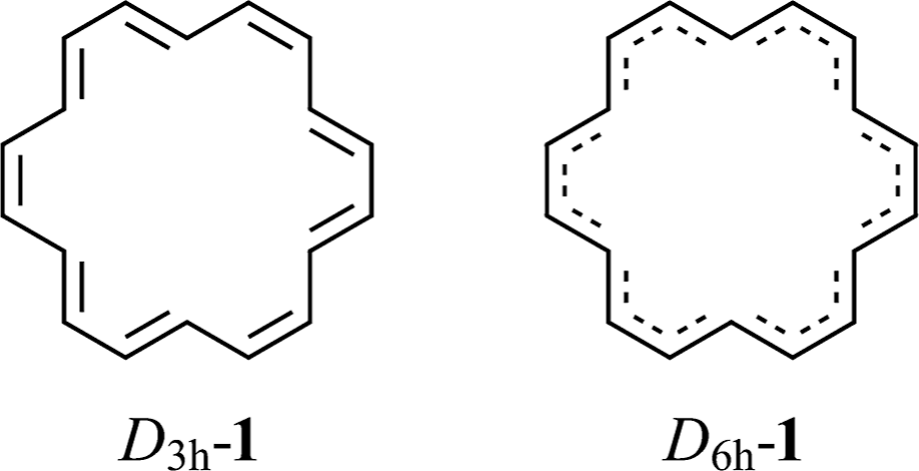
[18]Annulene (**1**) in *D*_3*h*_ and *D*_6*h*_ symmetry.

The 1959 synthesis of [18]annulene (1,3,5,7,9,11,13,15,17-cyclooctadecanonaene, **1**, Figure 1)^1^ was a milestone achievement because **1** is an approximately planar, virtually unstrained, and highly aromatic higher homologue of (4*n* + 2)π-aromatic^2^ benzene. While there is no doubt about the *aromatic* nature of **1** (as evident, e.g., from ^1^H NMR data),^3^ its *structure* has become an example of the suggestive power of high symmetry on human perception.

Prior to its synthesis, chemists doubted the viability of a completely planar structure, whether *D*_3*h*_ or *D*_6*h*_ (Figure 1), despite its unstrained carbon bond angles and the limited repulsive interactions between its internal protons. In 1952, Mislow predicted significant nonplanarity based on the van der Waals radii of the inner protons.^4^ In 1960, Coulson and Golebiewski^5^ said planarity “cannot be the case” and predicted deviations from it of 0.9 Å for the H atoms, along with a very large reduction in resonance energy due to nonplanarity.

The concept of a *D*_6*h*_-symmetric structure with only two types of C–C bonds dates back to Sondheimer’s second synthetic paper^6^ in which the final structure presented is that of *D*_6*h*_-**1**, based on unpublished NMR and incomplete X-ray diffraction data. In marked contrast to Sondheimer’s prediction, the X-ray structure published three years later^7^ revealed *C*_*i*_ rather than *D*_6*h*_ symmetry at 80 K. In the 1965 companion paper to the diffraction results, Hirshfeld and Rabinovich observed the *C*_*i*_ symmetry of the crystal but declared it “virtually certain” that an isolated molecule would have at least *S*_6_ symmetry, preferring the view that the symmetry was at least *D*_3*d*_. The authors claimed that nonbonded repulsion between adjacent, internal C–H groups was the cause of nonplanarity, but it was an insufficient explanation for the variation in C–C bond lengths. Hirshfeld and Rabinovich categorized all of the C–C bond lengths into two groups: the inner (1.382 ± 0.003) Å and the outer (1.419 ± 0.004) Å.^8^ The planar *D*_6*h*_-**1** structure, alongside interpretations of early NMR data,^3,9−12^ UV/vis spectra,^6,13^ photoelectron spectra,^14^ and thermochemical analysis,^15^ found its way into textbooks.

The 1995 X-ray analysis of Gorter et al.^16^ identified two molecular orientations in the crystal, the most common of which was identified as that reported in 1965.^7^ The structure of the dominant form was *C*_*i*_, but the authors claimed that it “agree[d] well” with a molecular *D*_6*h*_ structure. The small deviation from planarity was attributed to crystal packing, and the inversion disorder of *D*_3*h*_ molecules was excluded. In 2016, Lungerich et al.^17^ published a new synthetic route with improved yield along with an updated X-ray analysis. They found the same structural characteristics as those from 1995, while emphasizing the small face-to-face distance (3.16 Å) between stacked molecules in the crystal structure and how such close π–π contacts might lead to unique electronic properties.

In summary, the original X-ray analysis,^7,8^ along with re-examinations in 1995 at 111 K^16^ and more recently in 2016 at 100 K,^17^ is consistent but ambiguous with respect to the precise molecular, equilibrium geometry. The X-ray evidence shows a molecule far from a “short-long” C–C bond alternation pattern, instead imperfectly following a “short-short-long” pattern characteristic of the highest-symmetry point groups. Slight deviations from the latter pattern are present alongside a small departure from planarity. Taking the experimental evidence together, the expectation should be that **1** is slightly nonplanar, and its equilibrium geometry may well exhibit multiple C–C bond lengths and a lower-order point group like its smaller (4*n* + 2)π-electron analogues [10]^18^ and [14]annulene.^19^

The continuing interest in the exact structures of these larger annulenes arises from attempts to understand the rivalry between, on one side, aromaticity and sigma-bond equalization, and on the other side, repulsive interactions of the inner protons and the tendency for π-systems to localize,^20,21^ which causes bond alternation for the higher [4*n* + 2]annulenes.^22^ Second, as acenes^23−25^ and graphenes^26−28^ are very popular structures for novel organic electronic materials, understanding their electronic structures from all aspects is key to further developing this field.

Theoretical studies of the structure of **1** have been a challenge. Early studies (using, e.g., HF theory) are now obviously incorrect or fortuitous (vide infra, Table 2). Ab initio MP2^29,30^ as well as density functional theory (DFT) computations (BLYP,^31^ B3LYP,^31^ B3PW91,^29^ and BPW91^29^) include electron correlation crucial for the description of highly delocalized systems, and all yield a *D*_6*h*_ symmetric structure. However, in 2004 Wannere et al.^32^ reported that some density functional methods (e.g., KMLYP^33^ and BHHLYP^34^) predicted a *C*_2_ minimum. Furthermore, single-point energies with SCS-MP2^35^ and CCSD(T)^36,37^ energy points also favored the *C*_2_ structure. The authors then computed ^1^H NMR chemical shifts with DFT methods and argued that the observed, experimental NMR shifts were incompatible with a *D*_6*h*_ structure. The relative energies and vibrational frequencies reported for **1** in that 2004 paper highlighted the difficulty of finding a reliable, low-cost computational approach for similar problems.

**Figure 2:**
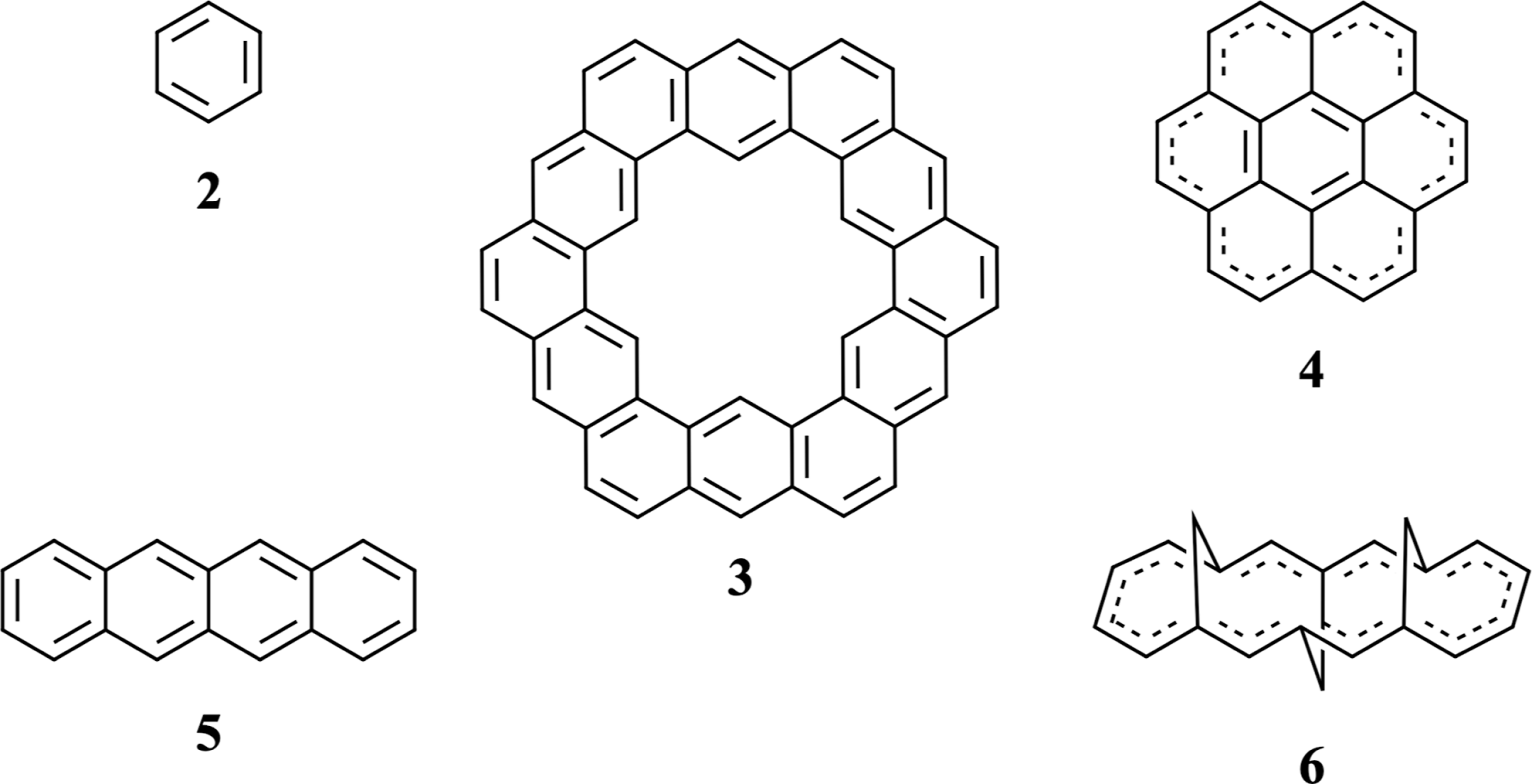
Compounds structurally related to [18]annulene, namely, benzene (**2**), kekulene (**3**), coronene(**4**), tetracene (**5**), and trismethano[18]annulene (**6**).

Among the vibrational frequencies reported by Wannere et al.,^32^ catching our attention were large imaginary values in the range 1075*i* – 1278*i* cm^–1^ and physically unrealistic values like 208,335 cm^–1^ for in-plane distortions at various levels. What are the origins of these abnormally large frequencies, and how reliable are related structural assignments? They may indicate a fundamental challenge for computing the structure of **1**, but, as we will show, also of related systems shown in Figure 2, such as benzene (**2**), kekulene (**3**), coronene (**4**), tetracene (**5**), and trismethano[18]annulene (**6**), some of which are models for understanding the electronic structures of graphenes, fullerenes, and carbon nanotubes.

In the present work, we report molecular orbital (MO) stability analyses at several levels of theory for compounds **1**–**6**. We endeavor to (1) assess the reliability of past and future theoretical results; (2) find a preferred approach for a correct description of compounds such as those in Figure 2; and (3) determine the equilibrium structure of the title compound **1** on the basis of currently feasible but rigorous quantum calculations. The potential energy surface for [18]annulene is clearly very flat, and we do not in this paper attempt to resolve dynamic effects on spectra. However, the proper starting point for a definitive analysis must be the correct description of the global minimum and the nearby potential energy surface (PES); this we attempt to establish.

## 2 Methods

In 1999, King et al.^18^ confirmed that MP2^38^ and B3LYP^34,39^ could not correctly describe the minimum-energy conformer of [10]annulene. While Hartree–Fock (HF) exaggerates bond alternation and deviation from planarity, MP2 and B3LYP instead overestimate delocalized, bond-equalized structures. The same tendencies are seen for [18]annulene; however, the energy differences between conformers are smaller in this case.

In this work, we have largely relied on CCSD(T) calculations.^36,37^ As suggested by the relative energies reported by Wannere et al.,^32^ our geometry optimizations and coupled-cluster (CC) results confirm that the density functional KMLYP^33^ closely tracks the CCSD(T) results for the PES of **1**. Therefore, we extensively employed the KMLYP functional. For comparison, we also report results determined with the SCS-MP2 approach,^35^ as well as with the M06-2X,^40^ BHHLYP,^34^ and CAM-B3LYP^41^ density functionals.

All of the DFT computations on **1** except the instability analyses were carried out with Psi4(42) which utilizes LibXC.^43^ For the other molecules, the structures were computed using the Gaussian03 program.^44^ For all molecules, orbital stability analyses were carried out using Gaussian03. The primary basis sets used were the 6-311+G(d,p) basis^45,46^ and the family of correlation consistent basis sets^47,48^ from cc-pVDZ (pVDZ) up to aug-cc-pVQZ (aug-pVQZ).^49−51^ There were also preliminary CC computations carried out using the double-ζ basis set (DZ) of Huzinaga and Dunning.^52,53^

The energies reported throughout this paper are electronic, equilibrium energies, or differences thereof. Given the difficulty of correctly computing relative energies of distinct geometric conformations and fully characterizing the PES around each of these stationary points, along with the nonharmonic nature of the PES, we have not included any estimate of zero-point vibrational energy.

For the DFT computations, it was found that larger than default grids were necessary to obtain energies and gradients that were consistent with one another. Within Psi4, the finer grid used is prescribed by the following keywords: “spherical points 1202” and “radial points 150”.

The CC computations were performed using the Psi4 and CFOUR(54) programs. The largest computations were carried out using the density-fitted frozen natural orbital coupled-cluster singles and doubles with perturbative triples [DF-FNO-CCSD(T)] code in Psi4 produced by DePrince and Sherrill.^55^ With the exception of the Brueckner CC frequencies discussed in Section 3.3 that were determined numerically, reported frequency analyses were based on analytic second derivative computations.

## 3 Results & Discussion

### 3.1 Stationary Points of [18]Annulene

Table 1 describes the results of optimizing *D*_6*h*_-**1**, carrying out a vibrational analysis, and following vibrational modes corresponding to imaginary frequencies downhill. For both KMLYP and M06-2X, there is one *b*_2u_ imaginary frequency for the *D*_6*h*_ stationary point. The corresponding normal mode represents an in-plane C–C bond alternation and leads to the *D*_3*h*_-**1** structure. Notwithstanding the minor difference of an extra imaginary frequency for the M06-2X *D*_3*h*_-**1** structure, KMLYP and M06-2X are in general agreement, suggesting that the global minimum is *C*_2_, lying ≈3 kcal mol^–1^ lower than *D*_6*h*_-**1**. It is worth noting that most of the relaxation energy from *D*_6*h*_-**1** as the symmetry is reduced occurs in the reduction to the *D*_3*h*_ subgroup; i.e., deviation from planarity plays a significant but far from dominant role.

**Table 1:** [18]Annulene Stationary Points with Common DFT Methods

symmetry	Δ*E*	description of the stationary point
KMLYP		
*D*_6*h*_	0.0	1218*i b*_2u_ (*b*_3u_ in *D*_2*h*_)
*D*_3*h*_	–2.6	61*i e*″ (*a*_2_ and *b*_1_ in *C*_2*v*_) [from following *b*_2u_]
*C*_s_	–2.9	80*i a*″ [from following *b*_1_]
*C*_2_	–3.0	minimum [from following *a*_2_ or *a*″ in *C*_1_]
M06-2X		
*D*_6*h*_	0.0	1078*i b*_2u_ (*b*_3u_ in *D*_2*h*_)
*D*_3*h*_	–2.2	115*i e*″ (*a*_2_ and *b*_1_ in *C*_2*v*_); 70*i a*_2_^″^ (*a*_2_ in *C*_2*v*_) [from following *b*_2u_]
*C*_s_	–2.9	121*i a*″ [from following *b*_1_]
*C*_2_	–3.2	minimum [from following either *a*_2_ or *a*″ in *C*_1_]
B3LYP		
*D*_6*h*_	0.0	minimum

Energies are relative electronic energies in kilocalories per mole computed with the 6-311+G(d,p) basis. Optimizations with KMLYP and M06-2X starting from *D*_3*d*_ structures (*C*_2*h*_ computational subgroup) converged to *D*_6*h*_. Optimizations with B3LYP starting from the *C*_s_, *C*_2_, and *D*_3*h*_ stationary points with KMLYP converged to *D*_6*h*_.

For B3LYP, *D*_6*h*_-**1** is a minimum, as previously found by others. To reduce the possibility of missing other significant minima, optimizations with B3LYP were carried out starting from each of the KMLYP stationary points, and all of these converged to *D*_6*h*_-**1**. Indeed, we have not observed *any* theoretical method to arrive at more than one minimum for **1**, in contrast to the situation for [10]annulene.^18^

The challenge of identifying a reliable low-cost, wave function-based method for systems like **1** is highlighted by the single-point energies reported in Table 2. HF strongly favors *C*_2_-**1** by 20 kcal mol^–1^, while MP2 favors *D*_6*h*_-**1** by 16 kcal mol^–1^. The DFT methods in Table 2 including exact exchange give similar results, but the frequently relied upon functionals B3LYP and M06-2X give opposite results. Compared to *D*_6*h*_-**1**, B3LYP places *C*_2_-**1** ≈ 4 kcal mol^–1^*higher* in energy, while M06-2X places *C*_2_-**1** ≈ 3 kcal mol^–1^*lower* in energy. Note that the differences in Table 2 are computed with *identical* geometries.

**Table 2:** Single-point Energies of **1** with Low-Cost Methods^a^

	HF	MP2	SCS-MP2	B3LYP	BHHLYP	M06-2X	KMLYP
*D*_6*h*_	0.0	0.0	0.0	0.0	0.0	0.0	0.0
*D*_3*h*_	–17.5	17.1	5.0	2.8	–2.2	–2.2	–2.6
*C*_*s*_	–19.7	16.4	3.8	3.7	–2.4	–2.8	–2.9
*C*_2_	–20.4	16.2	3.4	4.0	–2.4	–3.1	–3.0

a From 6-311+G(d,p) single-point energies at KMLYP/6-311+G(d,p) structures.

Applying CC theory to the problem, we first determined the optimized geometries of *D*_3*h*_-**1** and *D*_6*h*_-**1** and computed analytic frequencies at the CCSD/DZ level. Once again, we found one imaginary *b*_2u_ frequency for *D*_6*h*_-**1**, and the *D*_3*h*_ structure lies 12.2 kcal mol^–1^ lower in energy. Strikingly, the imaginary frequency of *D*_6*h*_-**1** is 10,679*i*—clearly unphysical. The three small imaginary frequencies obtained for the *D*_3*h*_-structure (e.g., 151*i e*″ and 142*i a*_2_″) are akin to those of M06-2X reported in Table 1. Clearly, a more complete basis set, better treatment of electron correlation, and suitable caution regarding instabilities (vide infra, Section 3.3) are in order.

As the next step, we optimized the structures of the key stationary points at the CCSD(T)/pVDZ level. All the structures are now much closer in energy as shown in Table 3. At the CCSD(T)/pVDZ level, the *C*_2_ structure remains the lowest energy but now only −2.2 kcal mol^–1^ relative to *D*_6*h*_-**1**. Executing single-point energies with a larger pVTZ basis reduces this energy difference slightly to −1.8 kcal mol^–1^.

**Table 3:** Relative Energies in kcal mol^–1^ for [18]annulene (**1**) Computed with Conventional CC Theory^a^

symmetry	CCSD(T)/pVDZ	CCSD/pVDZ	CCSD(T)/pVDZ	CCSD(T)/pVTZ
*D*_6*h*_	0.0	0.00	0.00	0.00
*D*_3*d*_	—b	–0.03	–0.08	–0.03
*D*_3*h*_	–1.1	–10.0	–1.3	–1.1
*C*_s_	–1.7	–11.4	–2.0	–1.6
*C*_2_	–1.9	–11.9	–2.2	–1.8
geometry	KMLYP/6-311+G(d,p)	CCSD(T)/pVDZ		

a Core electrons were frozen.

b No distinct *D*_3*d*_ structure was found with KMLYP.

The only qualitatively new stationary point found at the CCSD(T)/pVDZ level, relative to Tables 1 and 2, is the *D*_3*d*_ structure, which is very close *D*_6*h*_-**1**. According to CCSD(T)/pVTZ single-point energies, this stationary point is only −0.03 kcal mol^–1^ relative to *D*_6*h*_-**1**, and there is no distinct *D*_3*d*_ stationary point with KMLYP. Given that our additional CC calculations (Table 4) consistently keep the *D*_6*h*_ structure highest in energy, we did not perform further calculations directly on the *D*_3*d*_ structure. The (T) correction to the CCSD is seen to increase the relative energy of the *C*_2_ structure by ≈10 kcal mol^–1^. While repeating these computations with the full triple contribution is impractical at present, we report the analogous CCSDT computations for [10]annulene in Section 3.4.

**Table 4:** Relative Energies of [18]Annulene (**1**) Computed with DF-FNO CCSD(T)^a^

	pVDZ	aug-pVDZ	pVTZ	aug-pVTZ	pVQZ/pVTZ	aug-pVQZ/aug-pVTZ
Frozen-Core						
*D*_6*h*_	0.0	0.0	0.0	0.0	0.0	0.0
*D*_3*h*_	–1.0	–0.3	–0.9	–0.5	–0.6	–0.7
*C*_s_	–1.7	–0.3	–1.5	–1.0	–1.2	–1.1
*C*_2_	–1.8	–0.3	–1.7	–1.1	–1.3	–1.2
All-Electron						
*D*_6*h*_	0.0	0.00	0.0	0.0	0.0	0.0
*D*_3*h*_	–1.0	–0.3	–0.8	–0.4	–0.7	–0.5
*C*_s_	–1.6	–0.01	–0.8	0.5	–1.2	–1.0
*C*_2_	–1.7	0.02	–0.9	0.8	–1.4	–1.1

a Relative energies (in kcal mol^–1^) from DF-FNO CCSD(T) single-point computations at KMLYP/6-311+G(d,p) geometries.

Table 3 also reports very similar relative energies computed by using KMLYP-optimized geometries. This agreement supports the use of KMLYP as a cost-effective option for exploring the PES, as suggested by the earlier results of Wannere et al.^32^

The various conformations of **1** are close enough in energy that an extension to a larger basis set could be decisive. To address this possibility, we first carried out a series of computations to establish the accuracy of the density-fitted frozen natural orbital (DF-FNO) CC approach specifically for the conformations of **1**. The orbital cutoff value was optimized for efficiency and accuracy, and the results, reported in Supporting Information, demonstrate excellent agreement between DF-FNO and conventional CC theory applied to conformations of **1** with basis sets as large as pVTZ, including core electrons.

The calibrated DF-FNO CCSD(T) approach was then applied to compute single-point energies for the conformations of **1**. The results in Table 4 show that extension of the basis set does not change the qualitative picture of the PES from that of the CCSD(T)/pVDZ results; however, the PES is somewhat flattened by the increase in basis set size. Our single largest DF-FNO CCSD(T) calculation, with an aug-pVQZ/aug-pVTZ basis set on the C/H atoms, places the *C*_2_-**1** structure at −1.1 kcal mol^–1^ relative to the *D*_6*h*_-**1** one.

### 3.2 Geometric Structure of [18]Annulene (**1**)

**Figure 3:**
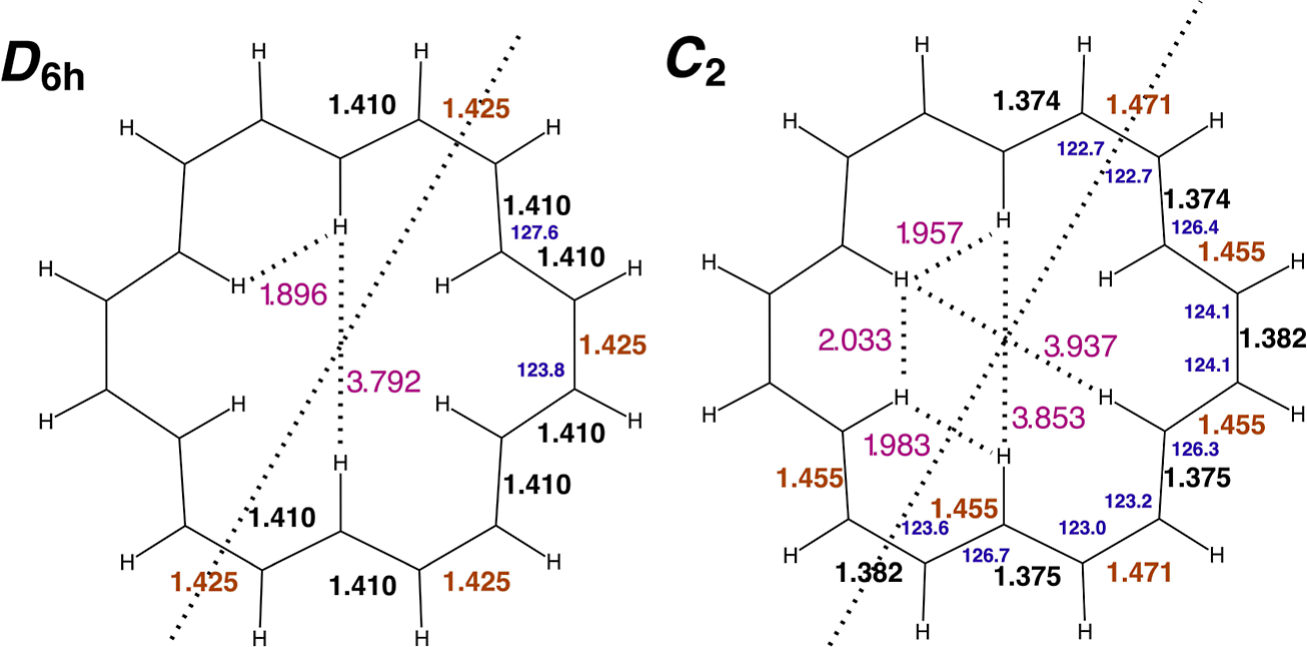
Comparison of *D*_6*h*_-**1** and *C*_2_-**1** structures optimized with CCSD(T)/pVDZ.

The CCSD(T)/pVDZ optimized structures of *D*_6*h*_-**1** and *C*_2_-**1** are shown in Figure 3. Relaxation to the lower symmetry is seen to increase the internal, adjacent H···H distance from 1.896 Å in *D*_6*h*_ to 1.957 Å, 1.983 Å, and 2.033 Å in *C*_2_. As far as the determination of the positions of the H atoms is possible by X-ray crystallography, Lungerich et al.^17^ reported distances ranging from 1.965 to 2.031 Å, in good agreement with the CC structures.

In 2015, Kwan and Liu^56^ applied quasiclassical dynamics to the interpretation of the NMR chemical shifts of **1**. Their ultimate conclusion was that, once dynamical effects are taken into account, low- (but still above 200 K) and high-temperature ^1^H and ^13^C shifts “provide strong evidence that the equilibrium structure of [18]annulene is planar and aromatic”. Kwan and Liu began by determining an approximate *D*_6*h*_ structure by computing the DLPNO–CCSD(T)/pVTZ energy points and fitting the PES within the *D*_6*h*_ subspace. The resulting structure (estimated to be within 0.1 kcal mol^–1^) compares well with the X-ray structure. Their final geometrical values are 1.413 and 1.395 Å and 123.7 and 127.4°. For comparison, the corresponding values for the CCSD(T)/pVDZ structure in Figure 3 are 1.425 and 1.410 Å and 123.8 and 127.6°. The bond angles agree within 0.2°, while the C–C bond lengths obtained with our smaller pVDZ basis set are longer as expected. However, the difference between the two lengths for each (0.018 vs 0.015 Å) is similar. The average C–C bond lengths reported by Lungerich et al. derived from X-ray data were 1.407 and 1.389 Å, also exhibiting a difference of 0.018 Å.

Kwan and Liu^56^ compared single-point energies of several density functionals to those obtained by DLPNO–CCSD(T) for 75 geometries generated by random displacement along low-frequency vibrational modes from stationary points of **1**. They concluded that M06-2X outperformed other functionals (including KMLYP) at paralleling the CC surface most closely. For comparison, we repeated our M06-2X computations with the smaller pVDZ basis used by Kwan and Liu (see Supporting Information). The results with the pVDZ basis are quite similar to those in Table 1 (with a 1056*i* cm^–1^ frequency for a *b*_2u_ mode of the *D*_6*h*_ structure and a *C*_2_ minimum lying 2.3 kcal mol^–1^ lower in energy).

It is not the goal of this paper to pursue dynamical effects, but we note that the two theoretical methods considered most reliable by Kwan and Liu, namely, DLPNO–CCSD(T) and (by virtue of its fit to it) M06-2X, predict a nonplanar, equilibrium geometry—that is, if “equilibrium” is taken to mean a vibrationless, zero-Kelvin geometry. The large basis set results of Table 4 conclusively show that, at least with CCSD(T) as the standard of electron correlation, the global minimum is not planar.

**Figure 4:**
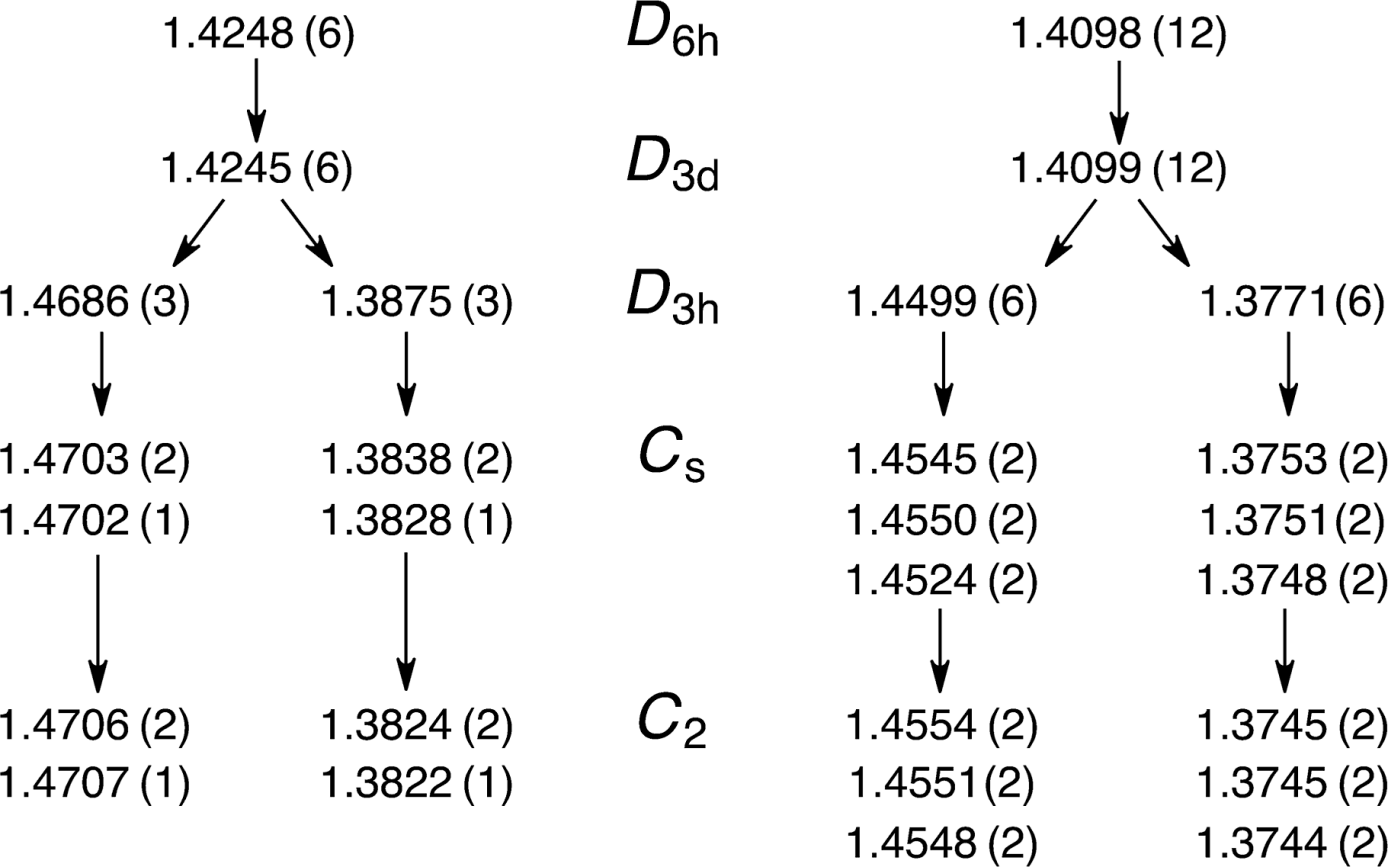
CCSD(T)/pVDZ bond lengths in Å. The number of each bond is given in parentheses.

C–C bond variation in the various stationary points can be examined in a variety of ways. Figure 4 shows the bond lengths computed at the CCSD(T)/pVDZ level of theory organized by length. The 2016 experimental structure^17^ displays *C*_*i*_ symmetry and has nine pairs of bond lengths, but these very nearly partition into a group of 12 longer and 6 shorter bonds of the same length. The structure is quite near *D*_3*d*_.

The distortion of the bond lengths that occurs when relaxing from the *D*_3*h*_ symmetry structure to the *C*_2_ symmetry structure is seen to be 0.002–0.005 Å. The longer bond lengths get longer, the shorter ones get shorter, and the resulting variation among the formerly equivalent bonds remains less than 0.001 Å.

Table 5 presents the shortest and longest C–C bond lengths of **1**, along with the maximum and average bond length difference between adjacent bonds, for a variety of point groups and theoretical methods. The molecular geometries from the X-ray studies were constructed and included for comparison. As expected, a wide range is apparent in the computed extent of the bond length variation.

**Table 5:** C–C Bond Lengths^a^

	shortest	longest	Δ_max_^b^	Δ_ave_^c^
*D*_6*h*_ (nofc) CCSD/DZ	1.4232	1.4375	0.0143	0.0095
*D*_3*d*_ (fc) CCSD(T)/pVDZ	1.4099	1.4245	0.0146	0.0098
*D*_6*h*_ (fc) CCSD(T)/pVDZ	1.4098	1.4248	0.0150	0.0100
*D*_6*h*_ BLYP/6-311+G(d,p)	1.3745	1.3900	0.0155	0.0103
*D*_6*h*_ KMLYP/6-311+G(d,p)	1.3746	1.3901	0.0155	0.0104
*D*_6*h*_ BHHLYP/6-311+G(d,p)	1.3838	1.3999	0.0161	0.0107
X-ray 2016 ref (17)	1.3867	1.4083	0.0215	0.0126
X-ray 1995 ref (16)	1.3776	1.4105	0.0329	0.0155
X-ray 1965 refs (7) and (8)	1.3731	1.4307	0.0515	0.0265
*D*_3*h*_ (fc) CCSD(T)/pVDZ	1.3771	1.4686	0.0915	0.0755
*D*_3*h*_ KMLYP/6-311+G(d,p)	1.3403	1.4349	0.0945	0.0781
*C*_s_ (fc) CCSD(T)/pVDZ	1.3748	1.4703	0.0955	0.0815
*C*_2_ (fc) CCSD(T)/pVDZ	1.3744	1.4707	0.0963	0.0832
*C*_s_ KMLYP/6-311+G(d,p)	1.3382	1.4370	0.0987	0.0837
*C*_2_ KMLYP/6-311+G(d,p)	1.3377	1.4369	0.0992	0.0855
*C*_2_ BHHLYP/6-311+G(d,p)	1.3470	1.4466	0.0996	0.0848
*C*_2_ CAM-B3LYP/6-311+G(d,p)	1.3489	1.4525	0.1036	0.0897
*C*_2_ (nofc) CCSD/pVDZ	1.3610	1.4763	0.1152	0.1039
*D*_3*h*_ (nofc) CCSD/DZ	1.3803	1.4991	0.1188	0.1035
*C*_2_ HF/pVDZ	1.3357	1.4747	0.1390	0.1288

a Bond lengths are in Å.

b Δ_max_ is the largest absolute difference between adjacent C–C bonds.

c Δ_ave_ is the mean absolute difference between adjacent C–C bonds (values of zero were included).

The values for Δ_max_ from the X-ray studies range from 0.052 Å in the 1965 structure to 0.022 Å in 2016. The *C*_2_ structures for both CCSD(T)/pVDZ and KMLYP/6-311+G(d,p) are much larger, with Δ_max_ = 0.096 and 0.099 Å, respectively. The corresponding differences for the *D*_6*h*_ stationary points of CCSD(T)/pVDZ and KMLYP/6-311+G(d,p), 0.015 and 0.016 Å, respectively, are too small but somewhat closer to the average X-ray values, even though neither of these methods gives a *D*_6*h*_-**1** minimum. As highlighted in the 2016 paper, however, in addition to the effect of dynamical time-averaging, the effects of crystal packing cannot be ignored as a possible cause of discrepancy between these geometric values.

To quantify the nonplanarity of each structure, Table 6 shows the distance of the carbon atoms (as well as the hydrogen atoms) from a plane defined by the position of the carbon atoms.

**Table 6:** Deviation from Planarity^a^

	carbons		hydrogens	
	RMS	max.	RMS	max.
*D*_3*d*_ (fc) CCSD(T)/pVDZ	0.017	0.029	0.066	0.105
X-ray 1995 ref (16)	0.048	0.084	0.089	0.188
X-ray 1965 refs (7) and (8)	0.051	0.084	0.124	0.251
X-ray 2016 ref (17)	0.052	0.087	0.093	0.197
*C*_2_ BHHLYP/6-311+G(d,p)	0.059	0.104	0.221	0.332
*C*_s_ KMLYP/6-311+G(d,p)	0.054	0.104	0.207	0.346
*C*_2_ KMLYP/6-311+G(d,p)	0.063	0.112	0.241	0.356
*C*_2_ CAM-B3LYP/6-311+G(d,p)	0.066	0.116	0.248	0.372
*C*_s_ (fc) CCSD(T)/pVDZ	0.063	0.116	0.236	0.380
*C*_2_ (fc) CCSD(T)/pVDZ	0.074	0.130	0.272	0.399
*C*_2_ (nofc) CCSD/pVDZ	0.092	0.164	0.337	0.507
*C*_2_ HF/pVDZ	0.107	0.190	0.385	0.593

a Distances in Å from the plane determined by the C atoms via SVD.

Here, the three experimental X-ray structures are in close agreement. In the most recent X-ray structure, the RMS distance of carbon atoms to the plane 0.052 Å compares well to 0.063 Å for the *C*_2_ KMLYP structure and less well to the 0.074 Å of the *C*_2_ CCSD(T)/pVDZ structure. By this metric, the effective nonplanarity observed by the NMR studies is clearly closer to the computed *C*_2_ structures than to a planar one (which would have value zero).

**Figure 5:**
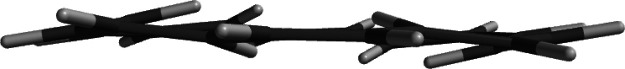
Side-view of the CCSD(T)/pVDZ minimum. The *C*_2_ axis is oriented toward the viewer.

Finally, for the purposes of visualization, we present a side-view of the *C*_2_ minimum computed using CCSD(T)/pVDZ in Figure 5.

### 3.3 On Instabilities

In general, abnormal vibrational frequencies^57−59^ can appear because of RHF–RHF (RHF = restricted HF) instabilities (“internal instabilities” where spatial-orbital symmetry breaking takes place^60^) of the reference wave function (indicated by a negative eigenvalue of the stability matrix) or because of interaction between the ground electronic state and nearby excited states (second-order Jahn–Teller (SOJT) or pseudo Jahn–Teller interactions).^61^ Both effects result in poles in the force constants. In the case of the SOJT interaction (or conical intersection), the interaction is real (at least, within the ability of the given method to describe it) and the force-constant pole is first order (different signs on each side of the singularity).^58,59^ However, in the case of orbital instabilities [sometimes called CPHF (coupled perturbed HF) instabilities], the interaction is artifactual and the pole is second order (same sign on both sides of the singularity) at correlated wave function levels of theory (e.g., CC and MP2) and first order with HF or DFT.^58,59,61^ For the CPHF poles, the MO Hessian as well as the stability matrix possess near-zero eigenvalues, while for the RHF–RHF instabilities, they are negative. The latter also leads to singularities in force constants and consequently to abnormal vibrational frequencies. To determine whether the unphysical vibrational frequencies for **1** are artifactual due to CPHF poles in the quadratic force constants, RHF–RHF instabilities, or whether they are due to real correlation poles indicating SOJT effects, we have examined the stability matrices at different levels of theory (Table 8 and Supporting Information). Apart from RHF-UHF instabilities (often indicative of low-lying triplet electronic states) found for all HF-based methods and DFT functionals employing large HF exchange contributions, one RHF–RHF instability was found at MP2/pVDZ and CCSD(T)/pVDZ, and near-zero eigenvalues in the HF stability matrix are present with all HF MO methods. In contrast, no unusual vibrational frequencies, instabilities, or near-zero eigenvalues of the [Kohn–Sham (KS)] stability matrices were observed at B3LYP/6-311++G(d,p), BLYP/6-311+G(d), OLYP/6-311+G(d,p), B3PW91/DZd, and BPW91/DZd for *D*_6*h*_-**1**.

As our investigation of the basis set influence at HF suggests (see Supporting Information), this result is not an artifact due to basis set dependence, as reported in a number of other cases^62^ for the imaginary frequencies of highly symmetrical structures of cyclic conjugated molecules. The near-zero eigenvalues found in the stability analyses at MP2, CCSD, and CCSD(T) with a pVDZ basis set in the *b*_2u_ irreducible representation block explain why the CCSD and MP2 vibrational analyses yield a large, unphysical vibrational frequency of *b*_2u_ symmetry. However, the question remains whether there is a true near degeneracy (SOJT) besides the artifactual CPHF poles for *D*_6*h*_-**1**. The fact that neither of the DFT stability computations reveal a similarly small eigenvalue of the KS MO Hessian suggests that the unusual DFT vibrational frequencies do not arise from the same kind of orbital near-instabilities that plague the ab initio results but instead are due to true SOJT interactions.

To discern MO instability effects versus SOJT interactions, we turned our attention to BHHLYP that lacks near-zero eigenvalues of the stability matrix. With this functional the imaginary vibrational frequency corresponds to a *b*_2u_ symmetric normal mode. Thus, if the symmetry breaking arises from a SOJT interaction, then the electronic states in question must be the ^1^A_g_ ground state and a ^1^B_2u_ excited state. Therefore, we examined the lowest lying excited singlet state of *D*_6*h*_-**1** (*E*_exc_ = 1.79 eV; 690 nm) that arises from an E_2u_–E_1g_ excitation; the direct product of E_2u_ and E_1g_ includes B_2u_ (as well as B_1u_ and E_1u_), which is the symmetry of the imaginary frequency with BHHLYP. This low-energy gap supports the notion that a SOJT interaction is the source of the symmetry breaking predicted at this level of theory. As expected for a SOJT effect, the *b*_2u_ force constant displays a singularity as a distortion along the symmetry preserving coordinate is introduced through a uniform elongation of both types of C–C distances within *D*_6*h*_ symmetry (Figure S1, Supporting Information). Although the pole is expected to be first order, this cannot be verified in this case as only one branch can be obtained because at elongations between 0.5320 and 0.5328 Å the *b*_2u_ imaginary frequency disappears and an e_2g_ imaginary frequency surfaces instead. The compression of the C–C bonds by about 0.05 Å leads to the disappearance of the imaginary *b*_2u_ frequency and the emergence of a new imaginary *b*_2g_ frequency. In addition, the TD-BHHLYP excitation energy drops as the uniform elongation of C–C bonds increases (Figure S2, Supporting Information). That is, at BHHLYP the broken symmetry structure is real. The near degeneracy of states combined with the notion of easy symmetry breaking of the *D*_3*h*_ structure to *C*_2_ at CCSD/DZ and convergence of *C*_*i*_, *C*_2_, *D*_3*d*_, and *D*_3*h*_ symmetric structures to *D*_6*h*_ with B3LYP/6-311+G(d,p) suggests that a high density of states exists for **1**. At BHHLYP/6-311+G(d,p), for example, the *D*_6*h*_ symmetric structure is less stable than the *D*_3*h*_ symmetric structure by 2.1 kcal mol^–1^, which lies 0.4 kcal mol^–1^ above the *C*_2_ minimum; the *C*_*i*_ symmetric structure converges to *D*_6*h*_-**1**.

In agreement with previous reports of the dramatic failure of the Brueckner approach in certain SOJT cases,^61,63^ attempts to compute the vibrational frequencies of *D*_6*h*_-**1** with CC Brueckner methods were unhelpful. For vibrations corresponding to most of the irreducible representations, the frequencies computed via finite differences of B-CCD/DZ energies were close to those determined via analytical derivatives at the CCSD/DZ level (see Supporting Information). However, the a_2g_ and e_2g_ B-CCD/DZ frequencies were clearly in error and were hugely dependent on the size of the displacement. Also, within the key *b*_1u_ group, the aforementioned 10, 679*i* CCSD/DZ frequency became, at the B-CCD/DZ level, 89, 561*i* or 26, 315*i*, depending on the displacement size. While such finite-difference computations are treacherous, as a consistent electronic state across all structures cannot be guaranteed, there is no evidence of an improved description by the use of Brueckner orbitals.

This problem might be overcome by the inclusion of full triple excitations. The maximal T2 amplitude^64^ with CCSD(T)/pVDZ reveals a value of 0.098 indicative of some potential closed-shell multireference character of the wave function. However, it has been argued in the literature that while MCSCF is traditionally considered to be a solution for symmetry breaking problems,^63−66^ the notion that SOJT effects “require a multireference treatment relies on flawed logic”,^59^ as empirical data show.^67^ The best treatment of symmetry breaking cases is currently believed to be provided by EOM(LR)-CC methods since they avoid orbital instability problems and cope well with SOJT situations. As we see in the case of **1**, CC theory is suitable for strong SOJT cases lacking near-singularities in the MO Hessian, but is not reliable for cases with near CPHF poles,^59,67^ a situation that might improve by including full triple excitations.^68^

In agreement with earlier studies, the DFT treatments show increased stability as compared to HF.^58,65,69^ The known ordering of stability is HF < B3LYP < BP < SVWN.^67^ As has been argued before,^59,65^ the exchange functional seems more important than the correlation functional in determining whether the symmetry is preserved: in several cases BLYP, B3LYP, BP86, PW91, B3PW91, EDF1, and SVWN yield symmetric densities, while hybrid functionals with higher degree of HF-exchange give artifactually symmetry broken solutions.^61,70^ However, the case of large unphysical frequencies due to near-zero eigenvalues of the KS MO Hessian at B3LYP is known.^58^ Thus, the sensitivity of the method toward symmetry breaking should be tested in each case. At B3LYP, which seems immune toward these problems in the case of **1**, the *D*_3*h*_, *D*_3*d*_, *C*_2_, and *C*_*i*_ symmetric structures of **1** all converge to *D*_6*h*_ symmetry. DFT (here B3LYP) itself tends to overestimate electron delocalization,^18^ favoring *D*_6*h*_-**1**. However, the admixture of HF-exchange with its “localizing” tendency to overcome this deficiency is potentially treacherous due to problems emerging with the HF-based approaches discussed above.

### 3.4 [10]Annulene

Given the large impact of electron correlation (see HF versus MP2 results in Table 2), the computation of a nonplanar geometry for **1** with CCSD(T) could be due to the inadequate treatment of electron correlation provided by the (T) correction. Since the direct exploration of the PES of **1** with full CCSDT is impractical at present, we revisited [10]annulene (**7**) to determine whether, for this comparable system, full CCSDT predictions would differ significantly from those of CCSD(T). In 1999, King et al.^18^ presented three minima and two transition states of [10]annulene, including CCSD(T)/DZd optimizations for the three minima. Comparing the results in that paper to those for **1** reported here, we observe similarities in the properties of the CCSD(T) relative energies: (1) they are not very sensitive to the method chosen to determine the structures; and (2) larger basis sets modestly contract their magnitude, without changing their ordering.

In Table 7, we report the relative energies of these five structures of [10]annulene with the same methods used for **1**. These results provide a direct comparison of these systems. More significantly, CCSDT computations are feasible for [10]annulene. These results provide significant evidence for the reliability of the (T) correction for **1** (though see the next section).

**Table 7:** Relative Energies in kcal mol^–1^ for Conformations of [10]Annulene^a^^,^^b^

	KMLYP/6-311G+(d,p)	CCSD(T)^c^/pVDZ	CCSDT^c^/pVDZ
*C*_2_ azulene-like (TS)	4.9	5.9	6.5
*C*_s_ boat (TS)	4.6	5.9	5.8
*C*_2_ naphthalene-like (min.)	0.8	0.8	0.8
*C*_s_ heart (min.)	0.5	3.4	3.9
*C*_2_ twist (min.)	0.0	0.0	0.0

a Structures provided in Supporting Information; names from ref (18).

b All energies computed at the KMLYP/6-311+G(d,p) geometries.

c Core electrons were frozen.

### 3.5 Other Benzenoid Systems

Our findings are troublesome, especially in view of the many computations on benzenoid hydrocarbons that attempt to determine their electronic properties in light of their use in molecular organic electronics (e.g., graphenes, acenes, polythiophenes, etc.). To explore whether the problems associated with **1** also apply to other structures we examined some of the most relevant larger aromatic systems here as well (Table 8).

**Table 8:**
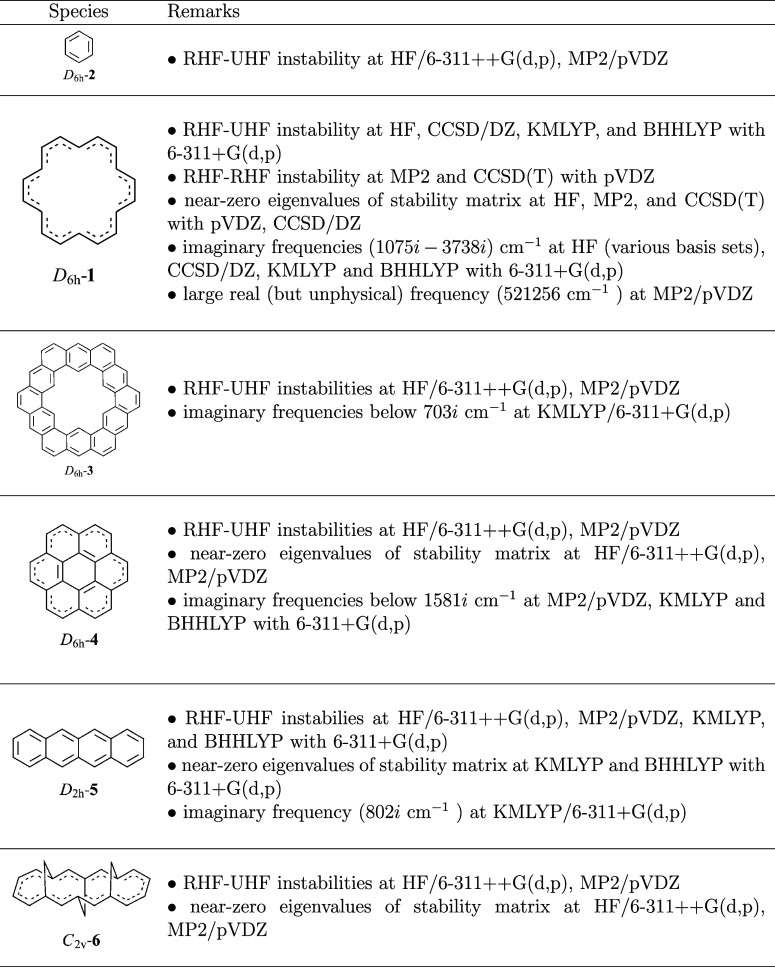
Behavior of Benzenoid Systems Related to [18]Annulene (**1**) at Different Levels of Theory

In agreement with our results for **2** with HF-based methods, a RHF-UHF instability has been reported.^71^ For *D*_6*h*_-**3**,^72−75^ only one paper^76^ mentions wave function instabilities as observed here; note, however, that the reported imaginary frequency at B3LYP/6-31G^76^ may well be due to basis-set deficiency since we do not find an instability at B3LYP/6-311++G(d,p). While we find instabilities, near-instabilities, and imaginary frequencies, no irregular features were reported for *D*_6*h*_-**4** at MP2,^77−79^ HF,^80−84^ and BHHLYP/6-31G.^85^ In agreement with our findings for **5**, triplet instabilities as well as near-zero eigenvalues of the stability matrices were found for *D*_2*h*_-symmetric naphthacene, pentacene, and hexacene at HF/6-31++G(d,p)^71^ and [6]cyclacene at B3LYP/6-31G(d).^86^ While HF singlet instabilities for the *D*_2*h*_-symmetric polyacenes with an odd number of benzene rings have been considered since 1970,^87^ both acetylenic and allenic cyclic C_18_ carbon cluster structures were found to display RHF-UHF instabilities at HF/6-31G(d).^88^ We also observe near-zero eigenvalues of the stability matrices at KMLYP and BHHLYP and one imaginary frequency at KMLYP for **5**. Even the nonplanar acene *C*_2*v*_-**6** displays near-zero eigenvalues of the stability matrix in addition to triplet instabilities with HF-based methods. Structures **2**–**6** do not show unusual vibrations, instabilities, or near-zero eigenvalues of the stability matrix at B3LYP/6-311++G(d,p). The X-ray determinations for **3**(89) and **4** also give nonplanar *C*_*i*_ symmetric structures, just as found for **1**.^90,91^

## 4 Conclusions

Owing to MO instabilities and especially the near-zero eigenvalues of the stability matrices, the computational results obtained with HF-based MO methods and DFT functionals including large HF exchange contributions for (even nonplanar) cyclic conjugated structures are not reliable. DFT approaches with lower HF admixture, such as B3LYP, appear in this regard more robust for the description of benzenoid structures.^92−94^

The most reliable approach, however, is thorough testing of the chosen quantum chemical method before accepting its results. We specifically recommend the careful examination of MO singlet-stability eigenvalues. The appearance of negative eigenvalues is not (necessarily) problematic, but near-zero (positive or negative) eigenvalues can lead to dramatic errors in vibrational frequencies and related properties and thus should be treated with skepticism.

For the specific challenge of [18]annulene, we confirm a nonplanar equilibrium structure. Our single largest DF-FNO CCSD(T) energy computation with an aug-pVQZ/aug-pVTZ basis set places the *C*_2_ minimum lower than the *D*_6*h*_ stationary point by 1.1 kcal mol^–1^.

## Supporting Information Available

Comparison of DF-FNO and conventional CC results for **1**, CCSD/DZ and B-CCD/DZ vibrational frequencies of *D*_6h_-**1**, total KMLYP and CC energies for [10]annulene, total CC energies and total DF-FNO CC energies for **1**, CCSD(T)/pVDZ structures and energies for *D*_6h_, *D*_3h_, *D*_3d_, *C*_s_, and *C*_2_ stationary points of **1**, KMLYP/6-311+G(d,p) structures of *D*_6h_, *D*_3h_, *D*_s_, and *C*_2_ stationary points of **1**, KMLYP/6-311+G(d,p) structures of [10]annulene, details of stability analyses for [18]annulene and structures **2**–**6**, dependence of the force constant and excitation energy of **1** on a uniform C–C bond elongation, total energies from stability analyses of **1**–**6**, and structures from stability analyses of **1**–**6** (PDF)
