# Supplementary material for: Structure of [18]Annulene Revisited: Challenges for Computing Benzenoid Systems
Source: J Phys Chem A. 2024 Feb 2;128(6):1098–108. doi: 10.1021/acs.jpca.3c07797 (PMC10875677; doi:10.1021/acs.jpca.3c07797)
Supplement: Supplementary file 1 — jp3c07797_si_001.pdf [file jp3c07797_si_001.pdf]

Supplemental Information for "The Structure of [18]Annulene Revisited: Challenges for Computing Benzenoid Systems"

- Rollin A. King, Dept. of Chemistry, Bethel University, St. Paul, Minnesota 55112, USA
- Peter R. Schreiner, Institute of Organic Chemistry, Justus Liebig University, Heinrich-Buff-Ring 17, 35392 Giessen, Germany
- T. Daniel Crawford, Dept. of Chemistry, Virginia Tech, Blacksburg, Virginia 24061, USA

SI Table of Contents

Table S1: Comparison of DF-FNO and conventional CC results for **1**.

Table S2 and Table S3: CCSD/DZ and B-CCD/DZ vibrational frequencies of D_{6h}-**1**.

Table S4: Total KMLYP and CC energies for [10]annulene for Table 7.

Table S5:Total CC energies for conformations of **1** for Table 3.

Table S6:Total DF-FNO CC energies for **1** for Table 4.

Table S7: Geometries (Ångstroms) and energies (au) for **1** for Table 1 (M06-2X, KMLYP, B3LYP).

Table S8: CCSD/DZ structures (Ångstroms) and energies (au) of D_{3h}-**1** and D_{6h}-**1**, as well as CCSD(T)/pVDZ structures and energies for D_{6h}, D_{3h}, D_{3d}, C_s, and C_2 stationary points of **1**.

Table S9: KMLYP/6-311+G(d,p) structures (Ångstroms) of D_{6h}, D_{3h}, C_s, and C_2 stationary points of **1**.

Table S10: Previous X-ray structures (Ångstroms).

Table S11: Additional structures (Ångstroms) of **1** used in Tables 5 or 6, including C_2 structures from BHHLYP/6-311+G(d,p), CAM-B3LYP/6-311+G(d,p), and HF/pVDZ, as well as D_{6h} structures from BHHLYP/6-311+G(d,p) and BLYP/6-311+G(d,p).

Table S12: KMLYP/6-311+G(d,p) structures (Ångstroms) of [10]annulene.

Table S13: Details of stability analyses for [18]annulene **1**.

Table S14: Details of stability analyses for structures **2**-**6**.

Figure S1: Dependence of the force constant for the b_{2u} imaginary frequency of **1** on a uniform C–C bond elongation.

Figure S2: Dependence of the excitation energy for the lowest singlet excited state of **1** on a uniform C–C bond elongation.

Table S15: Total energies from stability analyses of **1**.

Table S16: Total energies from stability analyses of **2**-**6**.

Table S17: Structures (Ångstroms) from stability analyses of **1**.

Table S18: Structures (Ångstroms) from stability analyses of **2**-**6**.

Table S1: DF-FNO vs. Conventional CCSD(T) for conformations of [18]annulene (1).[a]

	pVDZ/fc	pVTZ/fc	aug-pVDZ/fc	aug-pVDZ/all
Conventional CCSD(T)				
D_{6h}	0.00	0.00	0.00	0.00
D_{3h}	-1.14	-1.00	-0.39	-0.35
C_s	-1.76	-1.57	-0.32	-0.08
C_2	-1.93	-1.75	-0.34	-0.06
Density Fitted - Frozen Natural Orbital (5e-5) CCSD(T)				
D_{6h}	0.00	0.00	0.00	0.00
D_{3h}	-1.04	-0.86	-0.33	-0.29
C_s	-1.65	-1.49	-0.25	-0.01
C_2	-1.83	-1.72	-0.27	0.02

[a] Relative energies in kcal mol^{-1} from CCSD(T) single-point energies computed at KMLYP/6-311+G(d,p) geometries.

Table S2: Vibrational frequencies of D_{6h}-**1**

	CCSD/DZ Analytic Derivative	B-CCD/DZ Finite-Difference (0.001)[a]	B-CCD/DZ Finite-Difference (0.0005)[a]
a_{1g}	290.2	290.9	289.6
a_{1g}	1012.8	1014.3	1013.8
a_{1g}	1323.5	1324.3	1323.8
a_{1g}	1529.7	1531.1	1531.3
a_{1g}	3129.9	3131.7	3132.1
a_{1g}	3191.0	3193.7	3193.4
a_{1u}	484.3	484.6	
a_{1u}	844.3	844.6	
a_{2g}	681.8	$46318.1i$	808.3
a_{2g}	1262.1	$17683.6i$	2041.4
a_{2g}	1349.6	1294.7	2517.2
a_{2g}	1629.3	38041.4	2931.1
a_{2g}	3099.1	49934.4	3695.9
e_{1g}	113.2	94.2	
e_{1g}	440.8	449.4	
e_{1g}	790.9	771.7	
e_{1g}	826.0	827.2	
e_{1g}	930.0	927.7	
e_{1u}	354.5	356.2	
e_{1u}	652.1	653.7	
e_{1u}	1047.8	1064.7	
e_{1u}	1268.3	1287.4	
e_{1u}	1305.2	1311.6	
e_{1u}	1370.6	1373.7	
e_{1u}	1499.0	1506.7	
e_{1u}	1621.6	1625.2	
e_{1u}	3099.6	3102.2	
e_{1u}	3128.6	3131.9	
e_{1u}	3178.1	3183.0	
e_{2u}	71.9	68.4	
e_{2u}	141.0	115.5	
e_{2u}	323.8	323.6	
e_{2u}	762.7	766.0	
e_{2u}	806.7	802.9	
e_{2u}	936.9	857.8	

[a] Displacement size in amu$^{1/2}$ bohr.

Table S3: Vibrational frequencies of D_{6h}-**1**

	CCSD/DZ Analytic Derivative	B-CCD/DZ Finite-Difference (0.001)[a]	B-CCD/DZ Finite-Difference (0.0005)[a]
e_{2g}	123.3	111.6	148.7
e_{2g}	401.2	251.6	1511.4i
e_{2g}	636.5	416.1	639.1i
e_{2g}	1106.8	1007.3	762.3
e_{2g}	1246.9	1174.7	1139.8
e_{2g}	1267.4	1281.3	1246.6
e_{2g}	1346.3	1335.2	1363.6
e_{2g}	1469.5	1481.7	1676.9
e_{2g}	1559.3	1623.7	2491.4
e_{2g}	3100.1	3086.5	3039.9
e_{2g}	3127.4	3133.3	3121.3
e_{2g}	3152.4	3149.6	3396.9
b_{1g}	147.9	147.5	
b_{1g}	214.4	215.1	
b_{1g}	805.6	805.4	
b_{1g}	940.7	941.2	
b_{2u}	256.9	242.8	
b_{2u}	682.3	686.9	
b_{2u}	1273.2	1276.0	
b_{2u}	1468.7	1471.0	
b_{2u}	3102.5	3103.4	
b_{2u}	3140.1	3144.1	
b_{2g}	155.4	155.5	
b_{2g}	740.2	743.5	
b_{1u}	10679.1i	89560.6i	26315.2i
b_{1u}	323.2	12582.7i	11323.7i
b_{1u}	1133.3	940.6	776.3
b_{1u}	1275.2	13229.4	1497.2
b_{1u}	1490.0	1486.0	6224.9
b_{1u}	3126.7	3102.4	12457.6
a_{2u}	112.3	132.6	
a_{2u}	798.0	800.8	
a_{2u}	930.1	944.5	

[a] Displacement size in amu$^{1/2}$ bohr.

Table S4: Total energies in au of [10]annulene at KMLYP/6-311+G(d,p) geometries.

			KMLYP 6-311+G(d,p)	CCSD(T) pVDZ	CCSDT pVDZ
TS	C_2	azulene-like	-386.269669	-385.844425	-385.8447252
TS	C_s	boat	-386.270158	-385.844494	-385.8457752
Min	C_2	naphthalene-like	-386.276241	-385.852664	-385.8537955
Min	C_s	heart	-386.276650	-385.848522	-385.8488391
Min	C_2	twist	-386.277485	-385.853902	-385.8550179

Table S5: Total energies for [18]-annulene computed with (fc) coupled-cluster.

	CCSD(T)/pVDZ	CCSD/pVDZ	CCSD(T)/pVDZ	CCSD(T)/pVTZ
D_{6h}	-694.6066872	-694.4993224	-694.6333457	-695.2963230
D_{3d}	–	-694.4993776	-694.6334722	-695.2963646
D_{3h}	-694.6083843	-694.5152013	-694.6353696	-695.2980437
C_s	-694.6093612	-694.5175505	-694.6365102	-695.2989403
C_2	-694.6096428	-694.5182227	-694.6368103	-695.2992165
Geometry	KMLYP/6-311+G(d,p)	CCSD(T)/pVDZ	CCSD(T)/pVDZ	CCSD(T)/pVDZ

Table S6: Total energies in au for Table 4 of [18]-annulene from DF-FNO coupled-cluster.[a]

	pVDZ	aug-pVDZ	pVTZ	aug-pVTZ	pVQZ/ pVTZ	aug-pVQZ/ aug-pVTZ
			Frozen core			
D_{6h}	-694.60680	-694.72351	-695.29996	-695.35110	-695.49266	-695.51839
D_{3h}	-694.60845	-694.72404	-695.30134	-695.35196	-695.49368	-695.51957
C_s	-694.60944	-694.72392	-695.30234	-695.35265	-695.49450	-695.52008
C_2	-694.60971	-694.72394	-695.30270	-695.35285	-695.49478	-695.52024
			All-electron			
D_{6h}	-694.65139	-694.77541	-695.60082	-695.71376	-696.01322	-696.05471
D_{3h}	-694.65298	-694.77587	-695.60203	-695.71447	-696.01435	-696.05557
C_s	-694.65392	-694.77542	-695.60213	-695.71301	-696.01514	-696.05632
C_2	-694.65417	-694.77537	-695.60222	-695.71254	-696.01544	-696.05647

[a] Energies from DF-FNO CCSD(T) single-point computations at KMLYP/6-311+G(d,p) geometries. The orbital cutoff threshold was set at 5.0e-5.

Table S7: Geometries (Ångstroms) and energies (au) for **1** for Table 1 (M06-2X, KMLYP, B3LYP).

D_{6h} M06-2X\6-311+G(d,p); -696.4678574245; 1078i b_{2u}

H	0.0000000000	-1.8788943113	0.0000000000
H	1.6274904552	-0.9394681542	0.0000000000
H	1.6274904552	0.9394681542	0.0000000000
H	-0.0000000000	1.8788943113	0.0000000000
H	-1.6274904552	0.9394681542	0.0000000000
H	-1.6274904552	-0.9394681542	0.0000000000
C	-1.2494110921	-3.5699780215	-0.0000000000
C	1.2494110921	-3.5699780215	-0.0000000000
C	2.4671399150	-2.8670512772	0.0000000000
C	3.7166866597	-0.7030255410	-0.0000000000
C	3.7166866597	0.7030255410	-0.0000000000
C	2.4671399150	2.8670512772	0.0000000000
C	1.2494110921	3.5699780215	-0.0000000000
C	-1.2494110921	3.5699780215	-0.0000000000
C	-2.4671399150	2.8670512772	-0.0000000000
C	-3.7166866597	0.7030255410	-0.0000000000
C	-3.7166866597	-0.7030255410	-0.0000000000
C	-2.4671399150	-2.8670512772	0.0000000000
C	0.0000000000	-2.9599647003	0.0000000000
C	2.5636994032	-1.4800390804	0.0000000000
C	2.5636994032	1.4800390804	0.0000000000
C	-0.0000000000	2.9599647003	0.0000000000
C	-2.5636994032	1.4800390804	0.0000000000
C	-2.5636994032	-1.4800390804	0.0000000000
H	1.2906577858	-4.6556613483	0.0000000000
H	3.3866589501	-3.4457498253	0.0000000000
H	4.6775646029	-1.2100967759	0.0000000000
H	4.6775646029	1.2100967759	0.0000000000
H	3.3866589501	3.4457498253	0.0000000000
H	1.2906577858	4.6556613483	0.0000000000
H	-1.2906577858	4.6556613483	0.0000000000
H	-3.3866589501	3.4457498253	0.0000000000
H	-4.6775646029	1.2100967759	0.0000000000
H	-4.6775646029	-1.2100967759	0.0000000000
H	-3.3866589501	-3.4457498253	0.0000000000
H	-1.2906577858	-4.6556613483	0.0000000000

D_{3h} M06-2X\6-311+G(d,p); -696.4713075537; 115i e'', 70i a_2''

H	-0.0000000000	1.8826671707	0.0065845180
H	-0.0000000000	0.9470585090	1.6272597526
H	0.0000000000	-0.9470585090	1.6272597526
H	0.0000000000	-1.8826671707	0.0065845180
H	0.0000000000	-0.9356288703	-1.6338581727
H	-0.0000000000	0.9356288703	-1.6338581727
C	-0.0000000000	3.5617520846	-1.2187633460
C	-0.0000000000	3.5923927469	1.2845147583
C	-0.0000000000	2.9086378404	2.4689057232
C	-0.0000000000	0.7254179515	3.6940583029
C	0.0000000000	-0.7254179515	3.6940583029
C	0.0000000000	-2.9086378404	2.4689057232
C	0.0000000000	-3.5923927469	1.2845147583
C	0.0000000000	-3.5617520846	-1.2187633460
C	0.0000000000	-2.8363699650	-2.4752429029
C	0.0000000000	-0.6837954373	-3.7534698951
C	-0.0000000000	0.6837954373	-3.7534698951
C	-0.0000000000	2.8363699650	-2.4752429029
C	-0.0000000000	2.9636516122	-0.0015238988
C	-0.0000000000	1.4805309492	2.5674716595
C	0.0000000000	-1.4805309492	2.5674716595
C	0.0000000000	-2.9636516122	-0.0015238988
C	0.0000000000	-1.4831655779	-2.5659483278
C	-0.0000000000	1.4831655779	-2.5659483278
H	-0.0000000000	4.6783076052	1.3119350744
H	-0.0000000000	3.4754030729	3.3955873723
H	-0.0000000000	1.2216371347	4.6607142990
H	0.0000000000	-1.2216371347	4.6607142990
H	0.0000000000	-3.4754030729	3.3955873723
H	0.0000000000	-4.6783076052	1.3119350744
H	0.0000000000	-4.6470108248	-1.2723382001
H	0.0000000000	-3.4254447134	-3.3882855782
H	0.0000000000	-1.2029788029	-4.7076237516
H	-0.0000000000	1.2029788029	-4.7076237516
H	-0.0000000000	3.4254447134	-3.3882855782
H	-0.0000000000	4.6470108248	-1.2723382001

C_s M06-2X\6-311+G(d,p); -696.4724636971; 121i (a'')

H	-0.4077979220	-0.0485640251	1.9444873546
H	-0.1704832766	1.5903112572	0.9585056096
H	-0.1704832766	1.5903112572	-0.9585056096
H	-0.4077979220	-0.0485640251	-1.9444873546
H	0.5267626071	-1.5979491467	-0.9798725358
H	0.5267626071	-1.5979491467	0.9798725358
C	0.1534802031	-1.2078099575	3.5717207300
C	-0.0686135350	1.2813704210	3.6021157839
C	-0.1594252194	2.4540367220	2.9147389498
C	-0.2925811050	3.6555255544	0.7269984954
C	-0.2925811050	3.6555255544	-0.7269984954
C	-0.1594252194	2.4540367220	-2.9147389498
C	-0.0686135350	1.2813704210	-3.6021157839
C	0.1534802031	-1.2078099575	-3.5717207300
C	0.1596446334	-2.4610730693	-2.8364646145
C	0.2231503349	-3.7102171614	-0.6805630626
C	0.2231503349	-3.7102171614	0.6805630626
C	0.1596446334	-2.4610730693	2.8364646145
C	-0.1128644900	-0.0175839284	2.9863469290
C	-0.2226460810	2.5351898307	1.4826332677
C	-0.2226460810	2.5351898307	-1.4826332677
C	-0.1128644900	-0.0175839284	-2.9863469290
C	0.3178861693	-2.5285247650	-1.4941459976
C	0.3178861693	-2.5285247650	1.4941459976
H	0.0568855347	1.3174217708	4.6805462144
H	-0.1406838458	3.3877805587	3.4694298935
H	-0.3364185571	4.6219136909	1.2219104778
H	-0.3364185571	4.6219136909	-1.2219104778
H	-0.1406838458	3.3877805587	-3.4694298935
H	0.0568855347	1.3174217708	-4.6805462144
H	0.3635362818	-1.2441507323	-4.6376849207
H	0.0328820765	-3.3802504061	-3.4029708762
H	0.0987627155	-4.6573916001	-1.1979072069
H	0.0987627155	-4.6573916001	1.1979072069
H	0.0328820765	-3.3802504061	3.4029708762
H	0.3635362818	-1.2441507323	4.6376849207

C_2 M06-2X\6-311+G(d,p); -696.4728840414; Min.

H	0.4389473646	1.9158593994	-0.0109799506
H	0.4219904863	0.9406883100	1.6798192117
H	-0.4219904863	-0.9406883100	1.6798192117
H	-0.4389473646	-1.9158593994	-0.0109799506
H	0.3076371140	-0.9463036514	-1.6430772188
H	-0.3076371140	0.9463036514	-1.6430772188
C	-0.0377828203	3.5454108475	-1.2121155206
C	-0.0521981996	3.5707463367	1.2792926273
C	-0.0612092481	2.8924134189	2.4582894731
C	-0.0477673739	0.7253622976	3.6920627369
C	0.0477673739	-0.7253622976	3.6920627369
C	0.0612092481	-2.8924134189	2.4582894731
C	0.0521981996	-3.5707463367	1.2792926273
C	0.0377828203	-3.5454108475	-1.2121155206
C	-0.0389370669	-2.8228683347	-2.4714248475
C	0.0058571100	-0.6799039456	-3.7545210546
C	-0.0058571100	0.6799039456	-3.7545210546
C	0.0389370669	2.8228683347	-2.4714248475
C	0.1415627812	2.9576217806	-0.0076900723

C	0.1245919948	1.4705292429	2.5769027440
C	-0.1245919948	-1.4705292429	2.5769027440
C	-0.1415627812	-2.9576217806	-0.0076900723
C	0.0835542814	-1.4791736025	-2.5596689250
C	-0.0835542814	1.4791736025	-2.5596689250
H	-0.2549626247	4.6379472766	1.2932323772
H	-0.2688914973	3.4424468407	3.3717852018
H	-0.2631131223	1.2197140754	4.6360585692
H	0.2631131223	-1.2197140754	4.6360585692
H	0.2688914973	-3.4424468407	3.3717852018
H	0.2549626247	-4.6379472766	1.2932323772
H	0.2630884638	-4.6080449616	-1.2554710982
H	-0.1765825489	-3.4045310673	-3.3793708398
H	-0.0185037719	-1.2045457676	-4.7054171700
H	0.0185037719	1.2045457676	-4.7054171700
H	0.1765825489	3.4045310673	-3.3793708398
H	-0.2630884638	4.6080449616	-1.2554710982

D_{6h} KMLYP/6-311+G(d,p) (opt. in C_{2h} also lead to D_{6h})
-695.3775599650; 1218i b_{2u}

C	-2.5352615429	1.4637556833	0.0000000000
C	-3.6748153005	0.6950576045	0.0000000000
C	-3.6748153005	-0.6950576045	0.0000000000
C	-2.5352615429	-1.4637556833	0.0000000000
C	-2.4393485575	-2.8349883835	0.0000000000
C	-1.2354833607	-3.5300536658	0.0000000000
C	0.0000000000	-2.9275145936	0.0000000000
C	1.2354833607	-3.5300536658	0.0000000000
C	2.4393485575	-2.8349883835	0.0000000000
C	2.5352615429	-1.4637556833	0.0000000000
C	3.6748153005	-0.6950576045	0.0000000000
C	3.6748153005	0.6950576045	0.0000000000
C	2.5352615429	1.4637556833	0.0000000000
C	2.4393485575	2.8349883835	0.0000000000
C	1.2354833607	3.5300536658	0.0000000000
C	-0.0000000000	2.9275145936	0.0000000000
C	-1.2354833607	3.5300536658	0.0000000000
C	-2.4393485575	2.8349883835	0.0000000000
H	-1.6048201620	0.9265779886	0.0000000000
H	-4.6293276447	1.1978071066	0.0000000000
H	-4.6293276447	-1.1978071066	0.0000000000
H	-1.6048201620	-0.9265779886	0.0000000000
H	-3.3520080097	-3.4102297844	0.0000000000
H	-1.2773444355	-4.6080582133	0.0000000000
H	0.0000000000	-1.8531411073	0.0000000000
H	1.2773444355	-4.6080582133	0.0000000000
H	3.3520080097	-3.4102297844	0.0000000000
H	1.6048201620	-0.9265779886	0.0000000000
H	4.6293276447	-1.1978071066	0.0000000000
H	4.6293276447	1.1978071066	0.0000000000
H	1.6048201620	0.9265779886	0.0000000000
H	3.3520080097	3.4102297844	0.0000000000
H	1.2773444355	4.6080582133	0.0000000000
H	-0.0000000000	1.8531411073	0.0000000000
H	-1.2773444355	4.6080582133	0.0000000000
H	-3.3520080097	3.4102297844	0.0000000000

D_{3h} KMLYP/6-311+G(d,p); -695.3817798102; 61i e''

C	0.0000000000	-1.4662499962	-2.5380841474
C	0.0000000000	-0.6757612294	-3.7127052534
C	-0.0000000000	0.6757612294	-3.7127052534
C	-0.0000000000	1.4662499962	-2.5380841474
C	-0.0000000000	2.8035001299	-2.4470250772
C	-0.0000000000	3.5209384832	-1.2043942318
C	-0.0000000000	2.9311789353	-0.0007687553
C	-0.0000000000	3.5531843767	1.2711282256
C	-0.0000000000	2.8774215865	2.4415805153
C	-0.0000000000	1.4649244863	2.5388540013
C	-0.0000000000	0.7174342808	3.6514147860
C	0.0000000000	-0.7174342808	3.6514147860
C	0.0000000000	-1.4649244863	2.5388540013
C	0.0000000000	-2.8774215865	2.4415805153
C	0.0000000000	-3.5531843767	1.2711282256
C	0.0000000000	-2.9311789353	-0.0007687553
C	0.0000000000	-3.5209384832	-1.2043942318
C	0.0000000000	-2.8035001299	-2.4470250772
H	0.0000000000	-0.9216884045	-1.6119982452
H	0.0000000000	-1.1903139505	-4.6606535816
H	-0.0000000000	1.1903139505	-4.6606535816
H	-0.0000000000	0.9216884045	-1.6119982452
H	-0.0000000000	3.3889730028	-3.3533490421
H	-0.0000000000	4.5985747806	-1.2582737799
H	-0.0000000000	1.8568833936	0.0077931836
H	-0.0000000000	4.6314089731	1.2994889251
H	-0.0000000000	3.4410933372	3.3611716103
H	-0.0000000000	0.9351910100	1.6042055342
H	-0.0000000000	1.2095940652	4.6116146460
H	0.0000000000	-1.2095940652	4.6116146460
H	0.0000000000	-0.9351910100	1.6042055342
H	0.0000000000	-3.4410933372	3.3611716103
H	0.0000000000	-4.6314089731	1.2994889251
H	0.0000000000	-1.8568833936	0.0077931836
H	0.0000000000	-4.5985747806	-1.2582737799
H	0.0000000000	-3.3889730028	-3.3533490421

C_s KMLYP/6-311+G(d,p); -695.3822531157; 80i a''

C	0.0861542408	-0.0101378693	-2.9434113192
C	0.0322164868	1.2693856752	-3.5572524074
C	0.0901666895	2.4339384871	-2.8805300262
C	0.1481927613	2.5225541392	-1.4659298903
C	0.1750173297	3.6324253080	-0.7185146114
C	0.1750173297	3.6324253080	0.7185146114
C	0.1481927613	2.5225541392	1.4659298903
C	0.0901666895	2.4339384871	2.8805300262
C	0.0322164868	1.2693856752	3.5572524074
C	0.0861542408	-0.0101378693	2.9434113192
C	-0.1000529823	-1.1999674067	3.5268286531
C	-0.0858004433	-2.4401681542	2.8027697249
C	-0.2246163544	-2.5193945391	1.4739256125
C	-0.1202028340	-3.6878732489	0.6735872259
C	-0.1202028340	-3.6878732489	-0.6735872259
C	-0.2246163544	-2.5193945391	-1.4739256125
C	-0.0858004433	-2.4401681542	-2.8027697249
C	-0.1000529823	-1.1999674067	-3.5268286531
H	0.3141371443	-0.0238375796	-1.8923855009
H	-0.0718501934	1.3012739222	-4.6303677798
H	0.0559962375	3.3570732731	-3.4373030027
H	0.1380090620	1.5845816635	-0.9415194713
H	0.1797806439	4.5930141156	-1.2099437288
H	0.1797806439	4.5930141156	1.2099437288
H	0.1380090620	1.5845816635	0.9415194713
H	0.0559962375	3.3570732731	3.4373030027
H	-0.0718501934	1.3012739222	4.6303677798
H	0.3141371443	-0.0238375796	1.8923855009
H	-0.2583952686	-1.2459618850	4.5935207632
H	0.0461684832	-3.3477773512	3.3719796526
H	-0.4231503691	-1.5992502155	0.9529205548
H	0.0065056801	-4.6281936030	1.1867053010
H	0.0065056801	-4.6281936030	-1.1867053010
H	-0.4231503691	-1.5992502155	-0.9529205548
H	0.0461684832	-3.3477773512	-3.3719796526
H	-0.2583952686	-1.2459618850	-4.5935207632

C_2 KMLYP/6-311+G(d,p); -695.3824087567; Min.

C	0.0315565027	-1.4650689811	-2.5347961418
C	-0.0109243071	-0.6732269292	-3.7139695410
C	0.0109243071	0.6732269292	-3.7139695410
C	-0.0315565027	1.4650689811	-2.5347961418
C	0.0865283308	2.7945309679	-2.4452418085
C	0.0403576381	3.5101164640	-1.2000927110
C	0.1712602331	2.9241706777	-0.0046923235
C	0.0220118151	3.5382200895	1.2675529333
C	0.0010041093	2.8661992784	2.4343026527
C	0.1319432774	1.4557369594	2.5464737013
C	-0.0266153846	0.7178354126	3.6509577860
C	0.0266153846	-0.7178354126	3.6509577860

C	-0.1319432774	-1.4557369594	2.5464737013
C	-0.0010041093	-2.8661992784	2.4343026527
C	-0.0220118151	-3.5382200895	1.2675529333
C	-0.1712602331	-2.9241706777	-0.0046923235
C	-0.0403576381	-3.5101164640	-1.2000927110
C	-0.0865283308	-2.7945309679	-2.4452418085
H	0.2128524611	-0.9333097121	-1.6175018245
H	-0.0416942464	-1.1904710005	-4.6599756991
H	0.0416942464	1.1904710005	-4.6599756991
H	-0.2128524611	0.9333097121	-1.6175018245
H	0.2007434175	3.3742944089	-3.3484077676
H	-0.1216830838	4.5762374755	-1.2467046884
H	0.3905672226	1.8710561639	-0.0027422603
H	-0.1268771194	4.6063908647	1.2867263565
H	-0.1639282625	3.4213053531	3.3443220214
H	0.3750130731	0.9237450009	1.6434600721
H	-0.1998429677	1.2119388162	4.5949352955
H	0.1998429677	-1.2119388162	4.5949352955
H	-0.3750130731	-0.9237450009	1.6434600721
H	0.1639282625	-3.4213053531	3.3443220214
H	0.1268771194	-4.6063908647	1.2867263565
H	-0.3905672226	-1.8710561639	-0.0027422603
H	0.1216830838	-4.5762374755	-1.2467046884
H	-0.2007434175	-3.3742944089	-3.3484077676

D_{6h} B3LYP/6-311+G(d,p) (opt. in C_{2h} also lead to D_{6h})
-696.8230669028

H	-0.0478943656	1.8980206605	-0.0000006686
H	-0.0239414636	0.9490104875	1.6442389949
H	0.0239414636	-0.9490104875	1.6442389949
H	0.0478943656	-1.8980206605	-0.0000006686
H	0.0239466151	-0.9490098440	-1.6442385552
H	-0.0239466151	0.9490098440	-1.6442385552
C	-0.0904059841	3.5830904147	-1.2554086550
C	-0.0904144252	3.5830917247	1.2554066804
C	-0.0726355346	2.8784145277	2.4763214370
C	-0.0177823326	0.7046729312	3.7317241271
C	0.0177823326	-0.7046729312	3.7317241271
C	0.0726355346	-2.8784145277	2.4763214370
C	0.0904144252	-3.5830917247	1.2554066804
C	0.0904059841	-3.5830904147	-1.2554086550
C	0.0726243233	-2.8784125399	-2.4763224021
C	0.0177798292	-0.7046725494	-3.7317213981
C	-0.0177798292	0.7046725494	-3.7317213981
C	-0.0726243233	2.8784125399	-2.4763224021
C	-0.0751971566	2.9801522599	-0.0000008152
C	-0.0375995274	1.4900746206	2.5816927809
C	0.0375995274	-1.4900746206	2.5816927809
C	0.0751971566	-2.9801522599	-0.0000008152
C	0.0375967952	-1.4900727562	-2.5816917474
C	-0.0375967952	1.4900727562	-2.5816917474
H	-0.1178073640	4.6686946339	1.3044644659
H	-0.0874072700	3.4636830076	3.3922550195
H	-0.0304096817	1.2050052759	4.6967118261
H	0.0304096817	-1.2050052759	4.6967118261
H	0.0874072700	-3.4636830076	3.3922550195
H	0.1178073640	-4.6686946339	1.3044644659
H	0.1177973395	-4.6686930258	-1.3044672997
H	0.0873898309	-3.4636792894	-3.3922561520
H	0.0304032188	-1.2050041134	-4.6967077214
H	-0.0304032188	1.2050041134	-4.6967077214
H	-0.0873898309	3.4636792894	-3.3922561520
H	-0.1177973395	4.6686930258	-1.3044672997

D_{6h} M06-2X/pVDZ (opt. in C_s, C_2, D_{3h} also lead to D_{6h})
-696.3618648220; 1056*i* b_{2u}

H	0.0000000000	-1.8754013407	0.0000000000
H	1.6245345358	-0.9377550362	0.0000000000
H	1.6245345358	0.9377550362	0.0000000000
H	-0.0000000000	1.8754013407	0.0000000000
H	-1.6245345358	0.9377550362	0.0000000000
H	-1.6245345358	-0.9377550362	0.0000000000
C	-1.2518052017	-3.5761600758	0.0000000000
C	1.2518052017	-3.5761600758	0.0000000000
C	2.4713231091	-2.8722392674	0.0000000000
C	3.7232919810	-0.7040527098	0.0000000000
C	3.7232919810	0.7040527098	0.0000000000
C	2.4713231091	2.8722392674	0.0000000000
C	1.2518052017	3.5761600758	0.0000000000
C	-1.2518052017	3.5761600758	0.0000000000
C	-2.4713231091	2.8722392674	0.0000000000
C	-3.7232919810	0.7040527098	0.0000000000
C	-3.7232919810	-0.7040527098	0.0000000000
C	-2.4713231091	-2.8722392674	0.0000000000
C	0.0000000000	-2.9641029124	0.0000000000
C	2.5673486587	-1.4821364929	0.0000000000
C	2.5673486587	1.4821364929	0.0000000000
C	-0.0000000000	2.9641029124	0.0000000000
C	-2.5673486587	1.4821364929	0.0000000000
C	-2.5673486587	-1.4821364929	0.0000000000
H	1.2929301721	-4.6689573387	0.0000000000
H	3.3970358500	-3.4544330241	0.0000000000
H	4.6902781439	-1.2147673934	0.0000000000
H	4.6902781439	1.2147673934	0.0000000000
H	3.3970358500	3.4544330241	0.0000000000
H	1.2929301721	4.6689573387	0.0000000000
H	-1.2929301721	4.6689573387	0.0000000000
H	-3.3970358500	3.4544330241	0.0000000000
H	-4.6902781439	1.2147673934	0.0000000000
H	-4.6902781439	-1.2147673934	0.0000000000
H	-3.3970358500	-3.4544330241	0.0000000000
H	-1.2929301721	-4.6689573387	0.0000000000

C_2 M06-2X/pVDZ; -696.3654871054; Min.

H	0.3359944749	1.8969757574	-0.0047945712
H	0.3457787451	0.9398054122	1.6618974394
H	-0.3457787451	-0.9398054122	1.6618974394
H	-0.3359944749	-1.8969757574	-0.0047945712
H	0.2423159664	-0.9398684671	-1.6369973807
H	-0.2423159664	0.9398684671	-1.6369973807
C	-0.0356811113	3.5573800567	-1.2185947094
C	-0.0587796769	3.5810584215	1.2812070483
C	-0.0640557434	2.8992185983	2.4658812042
C	-0.0404516431	0.7251425698	3.7026622547
C	0.0404516431	-0.7251425698	3.7026622547
C	0.0640557434	-2.8992185983	2.4658812042
C	0.0587796769	-3.5810584215	1.2812070483
C	0.0356811113	-3.5573800567	-1.2185947094
C	-0.0241616720	-2.8347378249	-2.4772974074
C	0.0030005649	-0.6833834368	-3.7597792760
C	-0.0030005649	0.6833834368	-3.7597792760
C	0.0241616720	2.8347378249	-2.4772974074
C	0.1000031753	2.9614541746	-0.0054685827
C	0.0954984021	1.4753221304	2.5783460724
C	-0.0954984021	-1.4753221304	2.5783460724
C	-0.1000031753	-2.9614541746	-0.0054685827
C	0.0653429471	-1.4822162031	-2.5663418267
C	-0.0653429471	1.4822162031	-2.5663418267
H	-0.2301864909	4.6608797213	1.2998058190
H	-0.2401410268	3.4583363730	3.3889689998
H	-0.2154451251	1.2246004987	4.6602976844
H	0.2154451251	-1.2246004987	4.6602976844
H	0.2401410268	-3.4583363730	3.3889689998
H	0.2301864909	-4.6608797213	1.2998058190
H	0.2100063471	-4.6364959677	-1.2644185519
H	-0.1272033744	-3.4230992799	-3.3938518783
H	-0.0213146773	-1.2091532588	-4.7182276100
H	0.0213146773	1.2091532588	-4.7182276100
H	0.1272033744	3.4230992799	-3.3938518783
H	-0.2100063471	4.6364959677	-1.2644185519

Table S8: CCSD/DZ structures (Ångstroms) and energies (au) of D_{3h}-**1** and D_{6h}-**1**, as well as CCSD(T)/pVDZ structures and energies for D_{6h}, D_{3h}, D_{3d}, C_s, and C_2 stationary points of **1**.

D_{6h} (no fc) CCSD/DZ, -693.65710278, 10679i b_{2u}

C	0.0000000000	-3.0241017590	0.0000000000
C	1.2778164875	-3.6507522847	0.0000000000
C	2.5227359805	-2.9319976823	0.0000000000
C	2.6189489436	-1.5120508795	0.0000000000
C	3.8005524680	-0.7187546024	0.0000000000
C	3.8005524680	0.7187546024	0.0000000000
C	2.6189489436	1.5120508795	0.0000000000
C	2.5227359805	2.9319976823	0.0000000000
C	1.2778164875	3.6507522847	0.0000000000
C	0.0000000000	3.0241017590	0.0000000000
C	-1.2778164875	3.6507522847	0.0000000000
C	-2.5227359805	2.9319976823	0.0000000000
C	-2.6189489436	1.5120508795	0.0000000000
C	-3.8005524680	0.7187546024	0.0000000000
C	-3.8005524680	-0.7187546024	0.0000000000
C	-2.6189489436	-1.5120508795	0.0000000000
C	-2.5227359805	-2.9319976823	0.0000000000
C	-1.2778164875	-3.6507522847	0.0000000000
H	0.0000000000	-1.9295162568	0.0000000000
H	1.6710100937	-0.9647581284	0.0000000000
H	1.6710100937	0.9647581284	0.0000000000
H	0.0000000000	1.9295162568	0.0000000000
H	-1.6710100937	0.9647581284	0.0000000000
H	-1.6710100937	-0.9647581284	0.0000000000
H	1.3196919471	-4.7495207369	0.0000000000
H	3.4533596388	-3.5176471185	0.0000000000
H	4.7730515859	-1.2318736131	0.0000000000
H	4.7730515859	1.2318736131	0.0000000000
H	3.4533596388	3.5176471185	0.0000000000
H	1.3196919471	4.7495207369	0.0000000000
H	-1.3196919471	4.7495207369	0.0000000000
H	-3.4533596388	3.5176471185	0.0000000000
H	-4.7730515859	1.2318736131	0.0000000000
H	-4.7730515859	-1.2318736131	0.0000000000
H	-3.4533596388	-3.5176471185	0.0000000000
H	-1.3196919471	-4.7495207369	0.0000000000

D_{3h} (no fc) CCSD/DZ, -693.67656723, 151i e'', 142i a_2''

C	3.031757300	-0.001696966	0.000000000
C	3.644446519	-1.238601805	0.000000000
C	2.894883884	-2.536882368	0.000000000
C	1.517348265	-2.624730358	0.000000000
C	0.694398436	-3.855421201	0.000000000
C	-0.694398436	-3.855421201	0.000000000
C	-1.517348265	-2.624730358	0.000000000
C	-2.894883884	-2.536882368	0.000000000
C	-3.644446519	-1.238601805	0.000000000
C	-3.031757300	-0.001696966	0.000000000
C	-3.686091921	1.326343917	0.000000000
C	-2.991693486	2.529077284	0.000000000
C	-1.514409035	2.626427323	0.000000000
C	-0.749562634	3.775484172	0.000000000
C	0.749562634	3.775484172	0.000000000
C	1.514409035	2.626427323	0.000000000
C	2.991693486	2.529077284	0.000000000
C	3.686091921	1.326343917	0.000000000
H	-4.785399865	1.346235288	0.000000000
H	-3.558573889	3.471160210	0.000000000
H	1.226825976	-4.817395493	0.000000000
H	-1.226825976	-4.817395493	0.000000000
H	4.785399865	1.346235288	0.000000000
H	3.558573889	3.471160210	0.000000000
H	-1.937415637	0.010750051	0.000000000
H	-0.978017634	1.672476136	0.000000000
H	0.959398003	-1.683226182	0.000000000
H	-0.959398003	-1.683226182	0.000000000
H	1.937415637	0.010750051	0.000000000
H	0.978017634	1.672476136	0.000000000
H	3.496319562	-3.457531245	0.000000000
H	4.742469672	-1.299135935	0.000000000
H	-4.742469672	-1.299135935	0.000000000
H	-3.496319562	-3.457531245	0.000000000
H	-1.246150116	4.756667180	0.000000000
H	1.246150116	4.756667180	0.000000000

C_2 (no fc) CCSD/pVDZ, -694.56172675

C	0.7116390248	0.1960233273	-3.7183131634
C	1.4882972549	0.1284586170	-2.6025684776
C	2.8680339508	0.5978208465	-2.4827403130
C	3.5405677089	0.7177276158	-1.2983211756
C	2.9705229059	0.3949487274	0.0089162442
C	3.5007847422	0.7306082776	1.2166307565
C	2.7999001460	0.5005526304	2.4953594422
C	1.4464943267	0.3854564398	2.5824167101
C	0.6676829076	0.1464529391	3.7975529800
C	-0.6676829076	-0.1464529391	3.7975529800
C	-1.4464943267	-0.3854564398	2.5824167101
C	-2.7999001460	-0.5005526304	2.4953594422
C	-3.5007847422	-0.7306082776	1.2166307565
C	-2.9705229059	-0.3949487274	0.0089162442
C	-3.5405677089	-0.7177276158	-1.2983211756
C	-2.8680339508	-0.5978208465	-2.4827403130
C	-1.4882972549	-0.1284586170	-2.6025684776
C	-0.7116390248	-0.1960233273	-3.7183131634
H	1.0301863748	-0.2967022405	-1.7041112664
H	2.0133268173	-0.1354933578	0.0128470638
H	0.8742046251	0.5147407631	1.6583940494
H	-0.8742046251	-0.5147407631	1.6583940494
H	-2.0133268173	0.1354933578	0.0128470638
H	-1.0301863748	0.2967022405	-1.7041112664
H	3.3692574725	0.9166299555	-3.4055126974
H	4.5571341388	1.1310710408	-1.3115927218
H	4.4880336722	1.2099089449	1.2598942464
H	3.4089549560	0.4502699234	3.4077988853
H	1.1906700986	0.2371400826	4.7580049488
H	-1.1906700986	-0.2371400826	4.7580049488
H	-3.4089549560	-0.4502699234	3.4077988853
H	-4.4880336722	-1.2099089449	1.2598942464
H	-4.5571341388	-1.1310710408	-1.3115927218
H	-3.3692574725	-0.9166299555	-3.4055126974
H	-1.1450966712	-0.5510319661	-4.6630179640
H	1.1450966712	0.5510319661	-4.6630179640

C_2 (fc) CCSD(T)/pVDZ, -694.63681030

C	1.4891947956	-0.1225266010	-2.6052760625
C	2.9294273878	0.0517470375	-2.4921275082
C	3.6189424610	0.0420007770	-1.2940560601
C	2.9913579031	-0.1379652889	0.0060319661
C	3.5938090360	0.0300993237	1.2299835987
C	2.8626548122	-0.0426036350	2.5038371594
C	1.4960301345	0.0760324807	2.5906972118
C	0.6911094968	0.0040034497	3.8011652888
C	-0.6911094968	-0.0040034497	3.8011652888
C	-1.4960301345	-0.0760324807	2.5906972118
C	-2.8626548122	0.0426036350	2.5038371594
C	-3.5938090360	-0.0300993237	1.2299835987
C	-2.9913579031	0.1379652889	0.0060319661
C	-3.6189424610	-0.0420007770	-1.2940560601
C	-2.9294273878	-0.0517470375	-2.4921275082
C	-1.4891947956	0.1225266010	-2.6052760625
C	-0.7339006466	-0.0462923279	-3.7411155336
C	0.7339006466	0.0462923279	-3.7411155336
H	0.9510682528	-0.3984978688	-1.6892487308

H	3.4901777595	0.2453052079	-3.4190188096
H	4.7033190732	0.2288763178	-1.3108630518
H	1.9267122153	-0.4044473190	0.0067512717
H	4.6745669393	0.2358739684	1.2742393709
H	3.4525550807	-0.1686244061	3.4248963964
H	0.9529988577	0.2743890790	1.6576383545
H	1.2209235496	-0.0179056198	4.7653924228
H	-1.2209235496	0.0179056198	4.7653924228
H	-0.9529988577	-0.2743890790	1.6576383545
H	-3.4525550807	0.1686244061	3.4248963964
H	-4.6745669393	-0.2358739684	1.2742393709
H	-1.9267122153	0.4044473190	0.0067512717
H	-4.7033190732	-0.2288763178	-1.3108630518
H	-3.4901777595	-0.2453052079	-3.4190188096
H	-0.9510682528	0.3984978688	-1.6892487308
H	-1.2360459645	-0.2505735976	-4.6995480732
H	1.2360459645	0.2505735976	-4.6995480732

D_{3d} (C_{2h}) (fc) CCSD(T)/pVDZ, -694.63347218

C	-2.9911357046	-0.0290133112	0.0000000000
C	-3.6141922819	0.0053311486	-1.2642500816
C	-2.9019688284	-0.0053311486	-2.4978572895
C	-1.4955678523	0.0290133112	-2.5903995064
C	-0.7122234536	-0.0053311486	-3.7621073711
C	0.7122234536	0.0053311486	-3.7621073711
C	1.4955678523	-0.0290133112	-2.5903995064
C	2.9019688284	0.0053311486	-2.4978572895
C	3.6141922819	-0.0053311486	-1.2642500816
C	2.9911357046	0.0290133112	0.0000000000
C	3.6141922819	-0.0053311486	1.2642500816
C	2.9019688284	0.0053311486	2.4978572895
C	1.4955678523	-0.0290133112	2.5903995064
C	0.7122234536	0.0053311486	3.7621073711
C	-0.7122234536	-0.0053311486	3.7621073711
C	-1.4955678523	0.0290133112	2.5903995064
C	-2.9019688284	-0.0053311486	2.4978572895
C	-3.6141922819	0.0053311486	1.2642500816
H	-1.8971307327	-0.1048908921	0.0000000000
H	-4.7138198481	0.0326802131	-1.3048271084
H	-3.4869233475	-0.0326802131	-3.4298741831
H	-0.9485653663	0.1048908921	-1.6429634088
H	-1.2268965006	-0.0326802131	-4.7347012915
H	1.2268965006	0.0326802131	-4.7347012915
H	0.9485653663	-0.1048908921	-1.6429634088
H	3.4869233475	0.0326802131	-3.4298741831
H	4.7138198481	-0.0326802131	-1.3048271084
H	1.8971307327	0.1048908921	-0.0000000000
H	4.7138198481	-0.0326802131	1.3048271084
H	3.4869233475	0.0326802131	3.4298741831
H	0.9485653663	-0.1048908921	1.6429634088
H	1.2268965006	0.0326802131	4.7347012915
H	-1.2268965006	-0.0326802131	4.7347012915
H	-0.9485653663	0.1048908921	1.6429634088
H	-3.4869233475	-0.0326802131	3.4298741831
H	-4.7138198481	0.0326802131	1.3048271084

D_{3h} (C_{2v}) (fc) CCSD(T)/pVDZ, -694.63536956

C	0.0000000000	-2.9959429087	-0.0007612553
C	0.0000000000	-3.6073110419	-1.2347576386
C	0.0000000000	-2.8729870035	-2.5066441824
C	0.0000000000	-1.4986307208	-2.5941820395
C	0.0000000000	-0.6937518673	-3.8001384303
C	0.0000000000	0.6937518673	-3.8001384303
C	0.0000000000	1.4986307208	-2.5941820395
C	0.0000000000	2.8729870035	-2.5066441824
C	0.0000000000	3.6073110419	-1.2347576386
C	0.0000000000	2.9959429087	-0.0007612553
C	0.0000000000	3.6378923522	1.2992624741
C	0.0000000000	2.9441404849	2.5008759561
C	0.0000000000	1.4973121879	2.5949432949
C	0.0000000000	0.7343240384	3.7414018210
C	0.0000000000	-0.7343240384	3.7414018210
C	0.0000000000	-1.4973121879	2.5949432949
C	0.0000000000	-2.9441404849	2.5008759561
C	0.0000000000	-3.6378923522	1.2992624741
H	0.0000000000	0.9565898856	1.6417962948
H	0.0000000000	3.5180499969	3.4397230150
H	0.0000000000	4.7379125114	1.3268591617
H	0.0000000000	1.9001322420	0.0075329945
H	0.0000000000	4.7066709033	-1.2880326851
H	0.0000000000	3.4688044778	-3.4320802270
H	0.0000000000	0.9435423564	-1.6493292894
H	0.0000000000	1.2198625144	-4.7665821766
H	0.0000000000	-1.2198625144	-4.7665821766
H	0.0000000000	-0.9435423564	-1.6493292894
H	0.0000000000	-3.4688044778	-3.4320802270
H	0.0000000000	-4.7066709033	-1.2880326851
H	0.0000000000	-1.9001322420	0.0075329945
H	0.0000000000	-4.7379125114	1.3268591617
H	0.0000000000	-3.5180499969	3.4397230150
H	0.0000000000	-0.9565898856	1.6417962948
H	0.0000000000	-1.2378664255	4.7201129120
H	0.0000000000	1.2378664255	4.7201129120

D_{6h} (D_{2h}) (fc) CCSD(T)/pVDZ, -694.63334567

C	0.0000000000	-2.9924886462	0.0000000000
C	1.2647145600	-3.6153873513	0.0000000000
C	2.4986600107	-2.9029686131	0.0000000000
C	2.5915711881	-1.4962443231	0.0000000000
C	3.7633745707	-0.7124187382	0.0000000000
C	3.7633745707	0.7124187382	0.0000000000
C	2.5915711881	1.4962443231	0.0000000000
C	2.4986600107	2.9029686131	0.0000000000
C	1.2647145600	3.6153873513	0.0000000000
C	0.0000000000	2.9924886462	0.0000000000
C	-1.2647145600	3.6153873513	0.0000000000
C	-2.4986600107	2.9029686131	0.0000000000
C	-2.5915711881	1.4962443231	0.0000000000
C	-3.7633745707	0.7124187382	0.0000000000
C	-3.7633745707	-0.7124187382	0.0000000000
C	-2.5915711881	-1.4962443231	0.0000000000
C	-2.4986600107	-2.9029686131	0.0000000000
C	-1.2647145600	-3.6153873513	0.0000000000
H	0.0000000000	-1.8962335266	0.0000000000
H	1.3059794736	-4.7152988522	0.0000000000
H	3.4305788556	-3.4886608271	0.0000000000
H	1.6421864055	-0.9481167633	0.0000000000
H	4.7365583292	-1.2266380251	0.0000000000
H	4.7365583292	1.2266380251	0.0000000000
H	1.6421864055	0.9481167633	0.0000000000
H	3.4305788556	3.4886608271	0.0000000000
H	1.3059794736	4.7152988522	0.0000000000
H	0.0000000000	1.8962335266	0.0000000000
H	-1.3059794736	4.7152988522	0.0000000000
H	-3.4305788556	3.4886608271	0.0000000000
H	-1.6421864055	0.9481167633	0.0000000000
H	-4.7365583292	1.2266380251	0.0000000000
H	-4.7365583292	-1.2266380251	0.0000000000
H	-1.6421864055	-0.9481167633	0.0000000000
H	-3.4305788556	-3.4886608271	0.0000000000
H	-1.3059794736	-4.7152988522	0.0000000000

C_s (fc) CCSD(T)/pVDZ, -694.6365079

C	0.0070434186	0.1093674666	-3.0168164807
C	1.2328134702	-0.0587860171	-3.6161326020
C	2.4987266874	0.0296552301	-2.8736114119
C	2.5780427461	-0.1219193186	-1.5091968169
C	3.7677630101	0.0550723421	-0.6913844184
C	3.7677630101	0.0550723421	0.6913844184
C	2.5780427461	-0.1219193186	1.5091968169
C	2.4987266874	0.0296552301	2.8736114119
C	1.2328134702	-0.0587860171	3.6161326020
C	0.0070434186	0.1093674666	3.0168164807
C	-1.2976909110	-0.0264833675	3.6461864310
C	-2.4927050374	-0.0258080069	2.9483715057
C	-2.5756211667	0.0261807782	1.4992363348

C	-3.7190893925	0.0140279313	0.7351105024
C	-3.7190893925	0.0140279313	-0.7351105024
C	-2.5756211667	0.0261807782	-1.4992363348
C	-2.4927050374	-0.0258080069	-2.9483715057
C	-1.2976909110	-0.0264833675	-3.6461864310
H	0.0125503266	0.3800486398	-1.9531956328
H	1.2849529111	-0.2521845251	-4.6988333024
H	3.4180092086	0.2196383399	-3.4491881492
H	1.6507430438	-0.3825444212	-0.9827770442
H	4.7175147056	0.2415055767	-1.2151595115
H	4.7175147056	0.2415055767	1.2151595115
H	1.6507430438	-0.3825444212	0.9827770442
H	3.4180092086	0.2196383399	3.4491881492
H	1.2849529111	-0.2521845251	4.6988333024
H	0.0125503266	0.3800486398	1.9531956328
H	-1.3254998930	-0.1437104368	4.7400000841
H	-3.4340158217	-0.1097851130	3.5119656554
H	-1.6176986709	0.0433344333	0.9662100167
H	-4.6980165281	-0.0118651749	1.2376328054
H	-4.6980165281	-0.0118651749	-1.2376328054
H	-1.6176986709	0.0433344333	-0.9662100167
H	-3.4340158217	-0.1097851130	-3.5119656554
H	-1.3254998930	-0.1437104368	-4.7400000841

Table S9: KMLYP/6-311+G(d,p) structures (Ångstroms) of D_{6h}, D_{3h}, C_s, and C_2 stationary points of **1**.

D_{6h} KMLYP/6-311+G(d,p)

C	-2.5352615429	1.4637556833	0.0000000000
C	-3.6748153005	0.6950576045	0.0000000000
C	-3.6748153005	-0.6950576045	0.0000000000
C	-2.5352615429	-1.4637556833	0.0000000000
C	-2.4393485575	-2.8349883835	0.0000000000
C	-1.2354833607	-3.5300536658	0.0000000000
C	0.0000000000	-2.9275145936	0.0000000000
C	1.2354833607	-3.5300536658	0.0000000000
C	2.4393485575	-2.8349883835	0.0000000000
C	2.5352615429	-1.4637556833	0.0000000000
C	3.6748153005	-0.6950576045	0.0000000000
C	3.6748153005	0.6950576045	0.0000000000
C	2.5352615429	1.4637556833	0.0000000000
C	2.4393485575	2.8349883835	0.0000000000
C	1.2354833607	3.5300536658	0.0000000000
C	-0.0000000000	2.9275145936	0.0000000000
C	-1.2354833607	3.5300536658	0.0000000000
C	-2.4393485575	2.8349883835	0.0000000000
H	-1.6048201620	0.9265779886	0.0000000000
H	-4.6293276447	1.1978071066	0.0000000000
H	-4.6293276447	-1.1978071066	0.0000000000
H	-1.6048201620	-0.9265779886	0.0000000000
H	-3.3520080097	-3.4102297844	0.0000000000
H	-1.2773444355	-4.6080582133	0.0000000000
H	0.0000000000	-1.8531411073	0.0000000000
H	1.2773444355	-4.6080582133	0.0000000000
H	3.3520080097	-3.4102297844	0.0000000000
H	1.6048201620	-0.9265779886	0.0000000000
H	4.6293276447	-1.1978071066	0.0000000000
H	4.6293276447	1.1978071066	0.0000000000
H	1.6048201620	0.9265779886	0.0000000000
H	3.3520080097	3.4102297844	0.0000000000
H	1.2773444355	4.6080582133	0.0000000000
H	-0.0000000000	1.8531411073	0.0000000000
H	-1.2773444355	4.6080582133	0.0000000000
H	-3.3520080097	3.4102297844	0.0000000000

D_{3h} KMLYP/6-311+G(d,p)

C	0.0000000000	-1.4662499962	-2.5380841474
C	0.0000000000	-0.6757612294	-3.7127052534
C	-0.0000000000	0.6757612294	-3.7127052534
C	-0.0000000000	1.4662499962	-2.5380841474
C	-0.0000000000	2.8035001299	-2.4470250772
C	-0.0000000000	3.5209384832	-1.2043942318
C	-0.0000000000	2.9311789353	-0.0007687553
C	-0.0000000000	3.5531843767	1.2711282256
C	-0.0000000000	2.8774215865	2.4415805153
C	-0.0000000000	1.4649244863	2.5388540013
C	-0.0000000000	0.7174342808	3.6514147860
C	0.0000000000	-0.7174342808	3.6514147860
C	0.0000000000	-1.4649244863	2.5388540013
C	0.0000000000	-2.8774215865	2.4415805153
C	0.0000000000	-3.5531843767	1.2711282256
C	0.0000000000	-2.9311789353	-0.0007687553
C	0.0000000000	-3.5209384832	-1.2043942318
C	0.0000000000	-2.8035001299	-2.4470250772
H	0.0000000000	-0.9216884045	-1.6119982452
H	0.0000000000	-1.1903139505	-4.6606535816
H	-0.0000000000	1.1903139505	-4.6606535816
H	-0.0000000000	0.9216884045	-1.6119982452
H	-0.0000000000	3.3889730028	-3.3533490421
H	-0.0000000000	4.5985747806	-1.2582737799
H	-0.0000000000	1.8568833936	0.0077931836
H	-0.0000000000	4.6314089731	1.2994889251
H	-0.0000000000	3.4410933372	3.3611716103
H	-0.0000000000	0.9351910100	1.6042055342
H	-0.0000000000	1.2095940652	4.6116146460
H	0.0000000000	-1.2095940652	4.6116146460
H	0.0000000000	-0.9351910100	1.6042055342
H	0.0000000000	-3.4410933372	3.3611716103
H	0.0000000000	-4.6314089731	1.2994889251
H	0.0000000000	-1.8568833936	0.0077931836
H	0.0000000000	-4.5985747806	-1.2582737799
H	0.0000000000	-3.3889730028	-3.3533490421

C_s KMLYP/6-311+G(d,p)

C	0.0861542408	-0.0101378693	-2.9434113192
C	0.0322164868	1.2693856752	-3.5572524074
C	0.0901666895	2.4339384871	-2.8805300262
C	0.1481927613	2.5225541392	-1.4659298903
C	0.1750173297	3.6324253080	-0.7185146114
C	0.1750173297	3.6324253080	0.7185146114
C	0.1481927613	2.5225541392	1.4659298903
C	0.0901666895	2.4339384871	2.8805300262
C	0.0322164868	1.2693856752	3.5572524074
C	0.0861542408	-0.0101378693	2.9434113192
C	-0.1000529823	-1.1999674067	3.5268286531
C	-0.0858004433	-2.4401681542	2.8027697249
C	-0.2246163544	-2.5193945391	1.4739256125
C	-0.1202028340	-3.6878732489	0.6735872259
C	-0.1202028340	-3.6878732489	-0.6735872259
C	-0.2246163544	-2.5193945391	-1.4739256125
C	-0.0858004433	-2.4401681542	-2.8027697249
C	-0.1000529823	-1.1999674067	-3.5268286531
H	0.3141371443	-0.0238375796	-1.8923855009
H	-0.0718501934	1.3012739222	-4.6303677798
H	0.0559962375	3.3570732731	-3.4373030027
H	0.1380090620	1.5845816635	-0.9415194713
H	0.1797806439	4.5930141156	-1.2099437288
H	0.1797806439	4.5930141156	1.2099437288
H	0.1380090620	1.5845816635	0.9415194713
H	0.0559962375	3.3570732731	3.4373030027
H	-0.0718501934	1.3012739222	4.6303677798
H	0.3141371443	-0.0238375796	1.8923855009
H	-0.2583952686	-1.2459618850	4.5935207632
H	0.0461684832	-3.3477773512	3.3719796526
H	-0.4231503691	-1.5992502155	0.9529205548

H	0.0065056801	-4.6281936030	1.1867053010
H	0.0065056801	-4.6281936030	-1.1867053010
H	-0.4231503691	-1.5992502155	-0.9529205548
H	0.0461684832	-3.3477773512	-3.3719796526
H	-0.2583952686	-1.2459618850	-4.5935207632

*C*2 KMLYP/6-311+G(d,p)

C	0.0315565027	-1.4650689811	-2.5347961418
C	-0.0109243071	-0.6732269292	-3.7139695410
C	0.0109243071	0.6732269292	-3.7139695410
C	-0.0315565027	1.4650689811	-2.5347961418
C	0.0865283308	2.7945309679	-2.4452418085
C	0.0403576381	3.5101164640	-1.2000927110
C	0.1712602331	2.9241706777	-0.0046923235
C	0.0220118151	3.5382200895	1.2675529333
C	0.0010041093	2.8661992784	2.4343026527
C	0.1319432774	1.4557369594	2.5464737013
C	-0.0266153846	0.7178354126	3.6509577860
C	0.0266153846	-0.7178354126	3.6509577860
C	-0.1319432774	-1.4557369594	2.5464737013
C	-0.0010041093	-2.8661992784	2.4343026527
C	-0.0220118151	-3.5382200895	1.2675529333
C	-0.1712602331	-2.9241706777	-0.0046923235
C	-0.0403576381	-3.5101164640	-1.2000927110
C	-0.0865283308	-2.7945309679	-2.4452418085
H	0.2128524611	-0.9333097121	-1.6175018245
H	-0.0416942464	-1.1904710005	-4.6599756991
H	0.0416942464	1.1904710005	-4.6599756991
H	-0.2128524611	0.9333097121	-1.6175018245
H	0.2007434175	3.3742944089	-3.3484077676
H	-0.1216830838	4.5762374755	-1.2467046884
H	0.3905672226	1.8710561639	-0.0027422603
H	-0.1268771194	4.6063908647	1.2867263565
H	-0.1639282625	3.4213053531	3.3443220214
H	0.3750130731	0.9237450009	1.6434600721
H	-0.1998429677	1.2119388162	4.5949352955
H	0.1998429677	-1.2119388162	4.5949352955
H	-0.3750130731	-0.9237450009	1.6434600721
H	0.1639282625	-3.4213053531	3.3443220214
H	0.1268771194	-4.6063908647	1.2867263565
H	-0.3905672226	-1.8710561639	-0.0027422603
H	0.1216830838	-4.5762374755	-1.2467046884
H	-0.2007434175	-3.3742944089	-3.3484077676

Table S10: Previous X-ray structures (Ångstroms)

*C*i X-ray Bregman et al, Acta Cryst. (1965), 19 227.

C	-1.8698	-2.1955	0.4092
C	-2.2710	-2.4427	1.7196
C	-1.7824	-1.7410	2.8435
C	-0.8690	-0.7118	2.7626
C	-0.2605	-0.0394	3.8017
C	0.7045	0.9970	3.5981
C	1.1761	1.3810	2.3543
C	1.9950	2.4403	2.0498
C	2.3467	2.8243	0.7328
C	1.8698	2.1955	-0.4092
C	2.2710	2.4427	-1.7196
C	1.7824	1.7410	-2.8435
C	0.8690	0.7118	-2.7626
C	0.2605	0.0394	-3.8017
C	-0.7045	-0.9970	-3.5981
C	-1.1761	-1.3810	-2.3543
C	-1.9950	-2.4403	-2.0498
C	-2.3467	-2.8243	-0.7328
H	-1.1888	-1.3344	0.2950
H	-2.8714	-3.2064	1.8747
H	-2.1097	-2.0496	3.7113
H	-0.6454	-0.3744	1.8557
H	-0.4657	-0.2592	4.7486
H	1.0360	1.5360	4.4155
H	0.9375	0.8064	1.6558
H	2.2608	3.0048	2.8263
H	2.9155	3.5952	0.5710
H	1.1888	1.3344	-0.2950
H	2.8714	3.2064	-1.8747
H	2.1097	2.0496	-3.7113
H	0.6454	0.3744	-1.8557
H	0.4657	0.2592	-4.7486
H	-1.0360	-1.5360	-4.4155
H	-0.9375	-0.8064	-1.6558
H	-2.2608	-3.0048	-2.8263
H	-2.9155	-3.5952	-0.5710

*C*i X-ray Gorter et al, Acta Cryst. (1995), B51, 1036.

C	-1.8865	-2.1835	0.4142
C	-2.2744	-2.4375	1.7247
C	-1.7752	-1.7470	2.8377
C	-0.8536	-0.7155	2.7613
C	-0.2280	-0.0384	3.7969
C	0.7171	0.9786	3.5965
C	1.1929	1.3628	2.3518
C	2.0183	2.4221	2.0445
C	2.3631	2.7972	0.7292
C	1.8865	2.1835	-0.4142
C	2.2744	2.4375	-1.7247
C	1.7752	1.7470	-2.8377
C	0.8536	0.7155	-2.7613
C	0.2280	0.0384	-3.7969
C	-0.7171	-0.9786	-3.5965
C	-1.1929	-1.3628	-2.3518
C	-2.0183	-2.4221	-2.0445
C	-2.3631	-2.7972	-0.7292
H	-1.2030	-1.4838	0.3150
H	-2.9179	-3.1645	1.8899
H	-2.1008	-2.0072	3.6938
H	-0.6103	-0.4130	1.8517
H	-0.4657	-0.2929	4.7151
H	1.0595	1.4886	4.3810
H	0.8961	0.8259	1.6035
H	2.3773	2.9292	2.7871
H	3.0031	3.5055	0.6300
H	1.2030	1.4838	-0.3150
H	2.9179	3.1645	-1.8899
H	2.1008	2.0072	-3.6938
H	0.6103	0.4130	-1.8517
H	0.4657	0.2929	-4.7151
H	-1.0595	-1.4886	-4.3810
H	-0.8961	-0.8259	-1.6035
H	-2.3773	-2.9292	-2.7871
H	-3.0031	-3.5055	-0.6300

*C*i X-ray Lungerich et al, ChemComm (2016), 52, 4710.

C	1.671272	2.594905	5.348974
C	2.562146	1.975041	4.479812
C	3.910006	2.350600	4.325671
C	4.497695	3.422669	4.980284
C	5.830806	3.803977	4.958900
C	6.367871	4.820479	5.772237
C	5.626398	5.503097	6.728745
C	6.039046	6.531574	7.566106
C	5.178277	7.233353	8.426796
C	3.816467	6.985694	8.537611
C	2.925593	7.605559	9.406773
C	1.577733	7.230000	9.560914
C	0.990044	6.157931	8.906301
C	-0.343066	5.776623	8.927685
C	-0.880131	4.760121	8.114348

C	-0.138658	4.077503	7.157840
C	-0.551306	3.049026	6.320479
C	0.309463	2.347247	5.459789
H	2.032422	3.319678	5.935126
H	2.219850	1.216736	3.942402
H	4.473619	1.791572	3.717438
H	3.911567	3.975949	5.531027
H	6.451754	3.295726	4.371497
H	7.316797	5.053766	5.651839
H	4.686261	5.231008	6.818314
H	6.982218	6.802226	7.536250
H	5.554950	7.932737	8.977677
H	3.455318	6.260922	7.951459
H	3.267890	8.363864	9.944183
H	1.014121	7.789028	10.169147
H	1.576173	5.604651	8.355557
H	-0.964015	6.284873	9.515088
H	-1.829058	4.526833	8.234746
H	0.801479	4.349592	7.068271
H	-1.494478	2.778374	6.350335
H	-0.067210	1.647863	4.908908

Table S11: Additional structures (Ångstroms) of **1** used in Tables 5 or 6, including C_2 structures from BHHLYP/6-311+G(d,p), CAM-B3LYP/6-311+G(d,p), and HF/pVDZ, as well as D_{6h} structures from BHHLYP/6-311+G(d,p) and BLYP/6-311+G(d,p).

C_2 BHHLYP/6-311+G(d,p)

C	-1.4584519218	-0.2285422100	-2.5699741162
C	-2.8085287366	-0.6842036847	-2.4540817968
C	-3.4709772610	-0.8271112337	-1.2786185673
C	-2.9067317045	-0.5476598125	0.0043863131
C	-3.4473573110	-0.8049714355	1.2110856926
C	-2.7566926347	-0.5945733669	2.4646792723
C	-1.4301542655	-0.3805235065	2.5596607529
C	-0.6612873310	-0.1515955747	3.7429009094
C	0.6612873310	0.1515955747	3.7429009094
C	1.4301542655	0.3805235065	2.5596607529
C	2.7566926347	0.5945733669	2.4646792723
C	3.4473573110	0.8049714355	1.2110856926
C	2.9067317045	0.5476598125	0.0043863131
C	3.4709772610	0.8271112337	-1.2786185673
C	2.8085287366	0.6842036847	-2.4540817968
C	1.4584519218	0.2285422100	-2.5699741162
C	0.6966322298	0.1943360136	-3.6804060827
C	-0.6966322298	-0.1943360136	-3.6804060827
H	-0.9969119452	0.1157913722	-1.6624327947
H	-3.3161392509	-0.9659186889	-3.3634463264
H	-4.4770395088	-1.2159709425	-1.3036944176
H	-1.9292316233	-0.1012516614	0.0002648877
H	-4.4500091260	-1.2015324036	1.2646854324
H	-3.3500836482	-0.6351573586	3.3655419319
H	-0.8720422977	-0.4122211761	1.6419608173
H	-1.1665502563	-0.2454801621	4.6914897589
H	1.1665502563	0.2454801621	4.6914897589
H	0.8720422977	0.4122211761	1.6419608173
H	3.3500836482	0.6351573586	3.3655419319
H	4.4500091260	1.2015324036	1.2646854324
H	1.9292316233	0.1012516614	0.0002648877
H	4.4770395088	1.2159709425	-1.3036944176
H	3.3161392509	0.9659186889	-3.3634463264
H	0.9969119452	-0.1157913722	-1.6624327947
H	1.1340390791	0.4637919988	-4.6299920711
H	-1.1340390791	-0.4637919988	-4.6299920711

C_2 CAM-B3LYP/6-311+G(d,p)

C	-0.18415	0.70241	-3.68916
C	-0.18415	1.46778	-2.57835
C	-0.62543	2.82957	-2.45936
C	-0.75507	3.49497	-1.28282
C	-0.47490	2.92696	0.00592
C	-0.74081	3.46886	1.21228
C	-0.53846	2.77322	2.47122
C	-0.36216	1.43916	2.56520
C	-0.14104	0.66414	3.75425
C	0.14104	-0.66414	3.75425
C	0.36216	-1.43916	2.56520
C	0.53846	-2.77322	2.47122
C	0.74081	-3.46886	1.21228
C	0.47490	-2.92696	0.00592
C	0.75507	-3.49497	-1.28282
C	0.62543	-2.82957	-2.45936
C	0.18415	-1.46778	-2.57835
C	0.18415	-0.70241	-3.68916
H	0.17691	1.00095	-1.67081
H	-0.02203	1.94396	0.00378
H	-0.42352	0.87609	1.64297
H	0.42352	-0.87609	1.64297
H	0.02203	-1.94396	0.00378
H	-0.17691	-1.00095	-1.67081
H	-0.91034	3.34046	-3.37436
H	-1.13799	4.51100	-1.30480
H	-1.14142	4.47776	1.26296
H	-0.55295	3.37211	3.37782
H	-0.22680	1.17665	4.70781
H	0.22680	-1.17665	4.70781
H	0.55295	-3.37211	3.37782
H	1.14142	-4.47776	1.26296
H	1.13799	-4.51100	-1.30480
H	0.91034	-3.34046	-3.37436
H	0.46865	-1.14440	-4.64039
H	-0.46865	1.14440	-4.64039

C_2 HF/pVDZ

C	0.7098190892	0.1994608851	-3.6706067720
C	1.4882064283	0.0985683828	-2.5896181733
C	2.8634955203	0.5777073481	-2.4613320343
C	3.5255560843	0.6950384779	-1.3006386679
C	2.9721283260	0.3598446742	0.0106034160
C	3.4613392737	0.7378098180	1.1946271766
C	2.7650732864	0.4987285019	2.4724928014
C	1.4359567304	0.4038155859	2.5667370637
C	0.6525237151	0.1545006745	3.7767107477
C	-0.6525237151	-0.1545006745	3.7767107477
C	-1.4359567304	-0.4038155859	2.5667370637
C	-2.7650732864	-0.4987285019	2.4724928014
C	-3.4613392737	-0.7378098180	1.1946271766
C	-2.9721283260	-0.3598446742	0.0106034160
C	-3.5255560843	-0.6950384779	-1.3006386679
C	-2.8634955203	-0.5777073481	-2.4613320343
C	-1.4882064283	-0.0985683828	-2.5896181733
C	-0.7098190892	-0.1994608851	-3.6706067720
H	1.0518605232	-0.3587659563	-1.7141707217
H	2.0611902551	-0.2198890990	0.0159366803
H	0.8675902363	0.5570538738	1.6613784635
H	-0.8675902363	-0.5570538738	1.6613784635
H	-2.0611902551	0.2198890990	0.0159366803
H	-1.0518605232	0.3587659563	-1.7141707217
H	3.3551467135	0.9047975056	-3.3704590218
H	4.5245183400	1.1158596514	-1.3175841158
H	4.4055371925	1.2713313782	1.2431025956
H	3.3789260784	0.4223118742	3.3644156551
H	1.1641477158	0.2541927928	4.7272060774

H	-1.1641477158	-0.2541927928	4.7272060774
H	-3.3789260784	-0.4223118742	3.3644156551
H	-4.4055371925	-1.2713313782	1.2431025956
H	-4.5245183400	-1.1158596514	-1.3175841158
H	-3.3551467135	-0.9047975056	-3.3704590218
H	-1.1157635108	-0.5925290212	-4.5976277542
H	1.1157635108	0.5925290212	-4.5976277542

D$_{6h}$ BHHLYP/6-311+G(d,p)

C	0.0000000000	-0.0000000000	2.9556050082
C	1.2458925841	0.0000000000	3.5578454259
C	2.4582335207	0.0000000000	2.8578972196
C	2.5596223564	0.0000000000	1.4778015144
C	3.7041248530	0.0000000000	0.6999461148
C	3.7041248530	0.0000000000	-0.6999461148
C	2.5596223564	0.0000000000	-1.4778015144
C	2.4582335207	0.0000000000	-2.8578972196
C	1.2458925841	0.0000000000	-3.5578454259
C	0.0000000000	-0.0000000000	-2.9556050082
C	-1.2458925841	-0.0000000000	-3.5578454259
C	-2.4582335207	-0.0000000000	-2.8578972196
C	-2.5596223564	-0.0000000000	-1.4778015144
C	-3.7041248530	-0.0000000000	-0.6999461148
C	-3.7041248530	-0.0000000000	0.6999461148
C	-2.5596223564	-0.0000000000	1.4778015144
C	-2.4582335207	-0.0000000000	2.8578972196
C	-1.2458925841	-0.0000000000	3.5578454259
H	0.0000000000	-0.0000000000	1.8821246744
H	1.2929110733	0.0000000000	4.6358670391
H	3.3683200972	0.0000000000	3.4376256726
H	1.6299601935	0.0000000000	0.9410609074
H	4.6612286482	0.0000000000	1.1982390970
H	4.6612286482	0.0000000000	-1.1982390970
H	1.6299601935	0.0000000000	-0.9410609074
H	3.3683200972	0.0000000000	-3.4376256726
H	1.2929110733	0.0000000000	-4.6358670391
H	0.0000000000	-0.0000000000	-1.8821246744
H	-1.2929110733	-0.0000000000	-4.6358670391
H	-3.3683200972	-0.0000000000	-3.4376256726
H	-1.6299601935	-0.0000000000	-0.9410609074
H	-4.6612286482	-0.0000000000	-1.1982390970
H	-4.6612286482	-0.0000000000	1.1982390970
H	-1.6299601935	-0.0000000000	0.9410609074
H	-3.3683200972	-0.0000000000	3.4376256726
H	-1.2929110733	-0.0000000000	4.6358670391

D$_{6h}$ BLYP/6-311+G(d,p)

C	0.00000	-1.23550	3.53003
C	0.00000	0.00000	2.92766
C	0.00000	1.23550	3.53003
C	0.00000	2.43914	2.83479
C	0.00000	2.53490	1.46360
C	0.00000	3.67435	0.69493
C	0.00000	3.67435	-0.69493
C	0.00000	2.53490	-1.46360
C	0.00000	2.43914	-2.83479
C	0.00000	1.23550	-3.53003
C	0.00000	0.00000	-2.92766
C	0.00000	-1.23550	-3.53003
C	0.00000	-2.43914	-2.83479
C	0.00000	-2.53490	-1.46360
C	0.00000	-3.67435	-0.69493
C	0.00000	-3.67435	0.69493
C	0.00000	-2.53490	1.46360
C	0.00000	-2.43914	2.83479
H	0.00000	0.00000	1.85335
H	0.00000	1.60442	0.92650
H	0.00000	1.60442	-0.92650
H	0.00000	0.00000	-1.85335
H	0.00000	-1.60442	-0.92650
H	0.00000	-1.60442	0.92650
H	0.00000	1.27773	4.60799
H	0.00000	3.35177	3.41005
H	0.00000	4.62894	1.19747
H	0.00000	4.62894	-1.19747
H	0.00000	3.35177	-3.41005
H	0.00000	1.27773	-4.60799
H	0.00000	-1.27773	-4.60799
H	0.00000	-3.35177	-3.41005
H	0.00000	-4.62894	-1.19747
H	0.00000	-4.62894	1.19747
H	0.00000	-3.35177	3.41005
H	0.00000	-1.27773	4.60799

Table S12: KMLYP/6-311+G(d,p) structures (Ångstroms) of [10]annulene.

D$_{6h}$ KMLYP/6-311+G(d,p)

C	-2.5352615429	1.4637556833	0.0000000000
C	-3.6748153005	0.6950576045	0.0000000000
C	-3.6748153005	-0.6950576045	0.0000000000
C	-2.5352615429	-1.4637556833	0.0000000000
C	-2.4393485575	-2.8349883835	0.0000000000
C	-1.2354833607	-3.5300536658	0.0000000000
C	0.0000000000	-2.9275145936	0.0000000000
C	1.2354833607	-3.5300536658	0.0000000000
C	2.4393485575	-2.8349883835	0.0000000000
C	2.5352615429	-1.4637556833	0.0000000000
C	3.6748153005	-0.6950576045	0.0000000000
C	3.6748153005	0.6950576045	0.0000000000
C	2.5352615429	1.4637556833	0.0000000000
C	2.4393485575	2.8349883835	0.0000000000
C	1.2354833607	3.5300536658	0.0000000000
C	-0.0000000000	2.9275145936	0.0000000000
C	-1.2354833607	3.5300536658	0.0000000000
C	-2.4393485575	2.8349883835	0.0000000000
H	-1.6048201620	0.9265779886	0.0000000000
H	-4.6293276447	1.1978071066	0.0000000000
H	-4.6293276447	-1.1978071066	0.0000000000
H	-1.6048201620	-0.9265779886	0.0000000000
H	-3.3520080097	-3.4102297844	0.0000000000
H	-1.2773444355	-4.6080582133	0.0000000000
H	0.0000000000	-1.8531411073	0.0000000000
H	1.2773444355	-4.6080582133	0.0000000000
H	3.3520080097	-3.4102297844	0.0000000000
H	1.6048201620	-0.9265779886	0.0000000000
H	4.6293276447	-1.1978071066	0.0000000000
H	4.6293276447	1.1978071066	0.0000000000
H	1.6048201620	0.9265779886	0.0000000000
H	3.3520080097	3.4102297844	0.0000000000
H	1.2773444355	4.6080582133	0.0000000000
H	-0.0000000000	1.8531411073	0.0000000000
H	-1.2773444355	4.6080582133	0.0000000000
H	-3.3520080097	3.4102297844	0.0000000000

D$_{3h}$ KMLYP/6-311+G(d,p)

C	0.0000000000	-1.4662499962	-2.5380841474
C	0.0000000000	-0.6757612294	-3.7127052534
C	-0.0000000000	0.6757612294	-3.7127052534
C	-0.0000000000	1.4662499962	-2.5380841474
C	-0.0000000000	2.8035001299	-2.4470250772
C	-0.0000000000	3.5209384832	-1.2043942318
C	-0.0000000000	2.9311789353	-0.0007687553
C	-0.0000000000	3.5531843767	1.2711282256
C	-0.0000000000	2.8774215865	2.4415805153
C	-0.0000000000	1.4649244863	2.5388540013
C	-0.0000000000	0.7174342808	3.6514147860
C	0.0000000000	-0.7174342808	3.6514147860
C	0.0000000000	-1.4649244863	2.5388540013

C	0.0000000000	-2.8774215865	2.4415805153
C	0.0000000000	-3.5531843767	1.2711282256
C	0.0000000000	-2.9311789353	-0.0007687553
C	0.0000000000	-3.5209384832	-1.2043942318
C	0.0000000000	-2.8035001299	-2.4470250772
H	0.0000000000	-0.9216884045	-1.6119982452
H	0.0000000000	-1.1903139505	-4.6606535816
H	-0.0000000000	1.1903139505	-4.6606535816
H	-0.0000000000	0.9216884045	-1.6119982452
H	-0.0000000000	3.3889730028	-3.3533490421
H	-0.0000000000	4.5985747806	-1.2582737799
H	-0.0000000000	1.8568833936	0.0077931836
H	-0.0000000000	4.6314089731	1.2994889251
H	-0.0000000000	3.4410933372	3.3611716103
H	-0.0000000000	0.9351910100	1.6042055342
H	-0.0000000000	1.2095940652	4.6116146460
H	0.0000000000	-1.2095940652	4.6116146460
H	0.0000000000	-0.9351910100	1.6042055342
H	0.0000000000	-3.4410933372	3.3611716103
H	0.0000000000	-4.6314089731	1.2994889251
H	0.0000000000	-1.8568833936	0.0077931836
H	0.0000000000	-4.5985747806	-1.2582737799
H	0.0000000000	-3.3889730028	-3.3533490421

C_s KMLYP/6-311+G(d,p)

C	0.0861542408	-0.0101378693	-2.9434113192
C	0.0322164868	1.2693856752	-3.5572524074
C	0.0901666895	2.4339384871	-2.8805300262
C	0.1481927613	2.5225541392	-1.4659298903
C	0.1750173297	3.6324253080	-0.7185146114
C	0.1750173297	3.6324253080	0.7185146114
C	0.1481927613	2.5225541392	1.4659298903
C	0.0901666895	2.4339384871	2.8805300262
C	0.0322164868	1.2693856752	3.5572524074
C	0.0861542408	-0.0101378693	2.9434113192
C	-0.1000529823	-1.1999674067	3.5268286531
C	-0.0858004433	-2.4401681542	2.8027697249
C	-0.2246163544	-2.5193945391	1.4739256125
C	-0.1202028340	-3.6878732489	0.6735872259
C	-0.1202028340	-3.6878732489	-0.6735872259
C	-0.2246163544	-2.5193945391	-1.4739256125
C	-0.0858004433	-2.4401681542	-2.8027697249
C	-0.1000529823	-1.1999674067	-3.5268286531
H	0.3141371443	-0.0238375796	-1.8923855009
H	-0.0718501934	1.3012739222	-4.6303677798
H	0.0559962375	3.3570732731	-3.4373030027
H	0.1380090620	1.5845816635	-0.9415194713
H	0.1797806439	4.5930141156	-1.2099437288
H	0.1797806439	4.5930141156	1.2099437288
H	0.1380090620	1.5845816635	0.9415194713
H	0.0559962375	3.3570732731	3.4373030027
H	-0.0718501934	1.3012739222	4.6303677798
H	0.3141371443	-0.0238375796	1.8923855009
H	-0.2583952686	-1.2459618850	4.5935207632
H	0.0461684832	-3.3477773512	3.3719796526
H	-0.4231503691	-1.5992502155	0.9529205548
H	0.0065056801	-4.6281936030	1.1867053010
H	0.0065056801	-4.6281936030	-1.1867053010
H	-0.4231503691	-1.5992502155	-0.9529205548
H	0.0461684832	-3.3477773512	-3.3719796526
H	-0.2583952686	-1.2459618850	-4.5935207632

C_2 KMLYP/6-311+G(d,p)

C	0.0315565027	-1.4650689811	-2.5347961418
C	-0.0109243071	-0.6732269292	-3.7139695410
C	0.0109243071	0.6732269292	-3.7139695410
C	-0.0315565027	1.4650689811	-2.5347961418
C	0.0865283308	2.7945309679	-2.4452418085
C	0.0403576381	3.5101164640	-1.2000927110
C	0.1712602331	2.9241706777	-0.0046923235
C	0.0220118151	3.5382200895	1.2675529333
C	0.0010041093	2.8661992784	2.4343026527
C	0.1319432774	1.4557369594	2.5464737013
C	-0.0266153846	0.7178354126	3.6509577860
C	0.0266153846	-0.7178354126	3.6509577860
C	-0.1319432774	-1.4557369594	2.5464737013
C	-0.0010041093	-2.8661992784	2.4343026527
C	-0.0220118151	-3.5382200895	1.2675529333
C	-0.1712602331	-2.9241706777	-0.0046923235
C	-0.0403576381	-3.5101164640	-1.2000927110
C	-0.0865283308	-2.7945309679	-2.4452418085
H	0.2128524611	-0.9333097121	-1.6175018245
H	-0.0416942464	-1.1904710005	-4.6599756991
H	0.0416942464	1.1904710005	-4.6599756991
H	-0.2128524611	0.9333097121	-1.6175018245
H	0.2007434175	3.3742944089	-3.3484077676
H	-0.1216830838	4.5762374755	-1.2467046884
H	0.3905672226	1.8710561639	-0.0027422603
H	-0.1268771194	4.6063908647	1.2867263565
H	-0.1639282625	3.4213053531	3.3443220214
H	0.3750130731	0.9237450009	1.6434600721
H	-0.1998429677	1.2119388162	4.5949352955
H	0.1998429677	-1.2119388162	4.5949352955
H	-0.3750130731	-0.9237450009	1.6434600721
H	0.1639282625	-3.4213053531	3.3443220214
H	0.1268771194	-4.6063908647	1.2867263565
H	-0.3905672226	-1.8710561639	-0.0027422603
H	0.1216830838	-4.5762374755	-1.2467046884
H	-0.2007434175	-3.3742944089	-3.3484077676

Table S13. Overview of stability analyses, and key vibrational frequencies for various structures of [18]annulene at different levels of theory

		D_{6h}[a],[e]	D_{3h}[b]	D_{3d}[c],[e]	C_2
**HF/ 6-311G(*d,p*)**	spec. vibrations	B_{2u}, -11725.2555	A_1'', -190.7999<br>E'', -187.0085	A_{1u}, 40167.7067<br>E_u, -120.5687	-
	instabilities	B_{1u}, RHF→ UHF<br>B_{2u}, RHF→ UHF	B_2, RHF→ UHF<br>A_1, RHF→ UHF<br>B_2, RHF→ UHF	E_g, RHF→ UHF<br>E_g, RHF→ UHF<br>**E_g, RHF→ RHF**	B, RHF→ UHF<br>A, RHF→ UHF<br>B, RHF→ UHF
	near-0 eigenval. of stabil. matrix	S, B_{2u}, 0.0017649	-	**S, E_g, -0.0001471**	-
**HF/DZ**	spec. vibrations	B_{2u}, -6035.3827	E'', -153.4529<br>A_1'', -146.773		-
	instabilities	RHF→ UHF<br>**RHF→ UHF**	RHF→ UHF<br>RHF→ UHF<br>RHF→ UHF	conv. To D_{6h}	B, RHF→ UHF<br>A, RHF→ UHF<br>B, RHF→ UHF
	near-0 eigenval. of stabil. matrix	**-0.0062824**<br>S, 0.0058416	-		-
**HF/cc-pVDZ**	spec. vibrations	B_{2u}, -13737.543	E'', -181.4622<br>A_1'', -181.3488		-
	instabilities	RHF→ UHF<br>RHF→ UHF	RHF→ UHF<br>RHF→ UHF<br>RHF→ UHF	conv. To D_{6h}	B, RHF→ UHF<br>A, RHF→ UHF<br>B, RHF→ UHF
	near-0 eigenval. of stabil. matrix	S 0.0012820	-		-
**MP2/cc-pVDZ**	spec. vibrations	B_{2u}, 521256.2224<br>B_{2g}, -266.5714<br>E_{2u}, -137.729;<br>-137.4574	A_1', -2806.2592<br>A_1'',-332.8704<br>E'', -236.6039<br>E'', '-164.3107<br>A_2'', -161.0392	A_{1u},<br>519239.2739	
	instabilities	B_{1u}, RHF→ RHF<br>B_{1u}, RHF→ UHF<br>B_{2u}, RHF→ UHF	A_1', RHF→ UHF<br>A_2', RHF→ UHF<br>A_2', RHF→ UHF	**A_{1u}, RHF→ RHF**<br>A_{1u}, RHF→ UHF<br>A_{2u}, RHF→ UHF	
	near-0 eigenval. of stabil. matrix	S, B_{1u}, -0.0002799	-	**A_{1u}, -0.0001369**	
**CCSD/DZ**	spec. vibrations	B_{1u}, -10679.1263			
	instabilities	**B_{1u}, RHF→ UHF**<br>B_{2u}, RHF→ UHF	**A_1', 2×RHF→ UHF**<br>**A_2', 3×RHF→ UHF**		
	near-0 eigenval. of stabil. matrix	S, B_{2u}, 0.0027161<br>T, A_{1g}, 0.0034018<br>**B_{1u}, -0.0089945**<br>T, B_{1g}, 0.0034018	**2×-0.0040126**	conv. To D_{6h}	
**CCSD(T)/ cc-pVDZ**	spec. vibrations				
	instabilities	**B_{1u}, RHF→ RHF**<br>B_{1u}, RHF→ UHF<br>B_{2u}, RHF→ UHF			
	near-0 eigenval. of stabil. matrix	**B_{1u}, -0.0007774**			
**KMLYP/ 6-**	spec. vibrations	B_{2u}, -1256.2224<br>A, -92.9605<br>E_{2u}, -57.1475	A'', -166.7175<br>2×A'', -153.8498	-	-
	instabilities	RHF→UHF	**A', RHF→UHF**	-	-

	near-0 eigenval. of stabil. matrix	-	**T, *A'*, -0.0077873**	T, *B*, 0.0042360	T, *B*, 0.0060035
**BHLYP/6-311+G(*d,p*)**	spec. vibrations	B_{2u}, -1074.6787	*E''*, -98.6572<br>A''_1, -66,8690	*B*, -63.4454<br>*A*, -31.6033	-
	instabilities	RHF→UHF	RHF→UHF	RHF→UHF	RHF→UHF
	near-0 eigenval. of stabil. matrix	-	-	-	-

[a] For D_{6h}-**1** no special vibrations, instabilities or near-zero eigenvalues of the stability matrix were computed at B3LYP/6-311++G(*d*,*p*), B3PW91/DZd, PPW91/DZd, BLYP/6-311+G(*d*), OLYP/6-311+G(*d*,*p*). [b] At B3LYP/6-311+G(*d*,*p*) D_{3h} converges to D_{6h} [c] At OLYP/6-311+G(*d*,*p*) D_{3d} converges to D_{6h} and C_i to D_{3d}, while no anomalies can be found for C_2, D_{3d}, D_{3h}. [e] The C_i symmetric structure converges at CCSD/DZ to C_1, at MP2/cc-pVDZ to D_{3d}, at BHLYP/6-311+G(*d*,*p*) to D_{6h}, and at HF with different basis sets to D_{6h}.

Table S14. Overview of stability analyses and key vibrational frequencies of other benzenoid systems **2-6** at different levels of theory.

		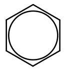 **2**[d],[e]	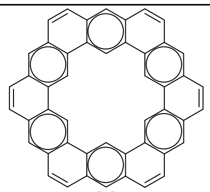 **3**[e]	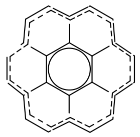 **4**[e]	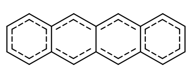 **5**[e]	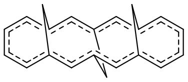 **6**[e]
	PG	D_{6h}	D_{6h}	D_{6h}	D_{2h}	C_{2v}
**HF/ 6-311++G(*d,p*)**	spec. Vibrations[a]	–	–	–	–	–
	instabilities	RHF→UHF	2×RHF→UHF	RHF→UHF	B_{1u}, RHF→UHF<br>B_{3g}, RHF→UHF	A_2, RHF→UHF<br>B_1, RHF→UHF
	[b]near-zero eigenval. of stabil. matrix	–	–	T, E_{2g}, 0.0072689	–	T, A_2, 0.0055202<br>T, B_1, 0.0062263
**MP2/ cc-pVDZ**	spec. vibrations[a]	–		B_{2g}, –1581.1127<br>E_{2u}, –666.1633	–	–
	instabilities	RHF→UHF	RHF→UHF<br>E_{2g}, RHF→UHF	RHF→UHF	B_{1u}, RHF→UHF<br>B_{3g}, RHF→UHF	**2×A_2, RHF→UHF**<br>B_1, RHF→UHF
	[b]near-zero eigenval. of stabil. matrix	–	–	T, E_{2g}, 0.0011787	–	**–0.0076291**[c]<br>T, B_1, 0.0073685
**KMLYP/ 6-311+G(*d,p*)**	spec. vibrations[a]	–	B_{2u}, –702.9507<br>B_{3g}, –568.4069<br>A_g, –568.4021<br>B_{2u}, –295.9885<br>B_{1u}, –295.9770<br>B_{3g}, –130.8091	–596→D_{3h}	–802→C_{2h}	–
	instabilities	–	–	–	B_{1u}, RHF→UHF	–
	[b]near-zero eigenval. of stabil. matrix	–	–	–	–0.0007022	–
**BHLYP/ 6-[illegible]**	spec. vibrations[a]	–	–	–261→C_i	–	–
	instabilities	–	–	–	***B*, RHF→UHF**	–
	[b]near-zero eigenval. of stabil. matrix	–	–	–	–**0.0007402**[c]	–

[a] The vibrational frequencies are given in cm^{-1}. [b]S stands for singlet, T symbolizes triplet. [c]Corresponding pairs of instabilities and near-zero eigenvalues of stability matrix are in bold. [d]For benzene at MP4/cc-pVDZ, CCSD/DZ, and CCSD(T)/cc-pVDZ the same behaviour as at MP2/cc-pVDZ is observed. [e]For none of the cyclic conjugated molecules **2**-**6** no special vibrations, instabilities or near-zero eigenvalues of the stability matrix are observed at B3LYP/6-311++G(*d*,*p*).

Figure S1. Dependence of the force constant for the b_{2u} imaginary frequency on a uniform CC bond elongation computed at BHHLYP/6-311+G(*d*,*p*)).

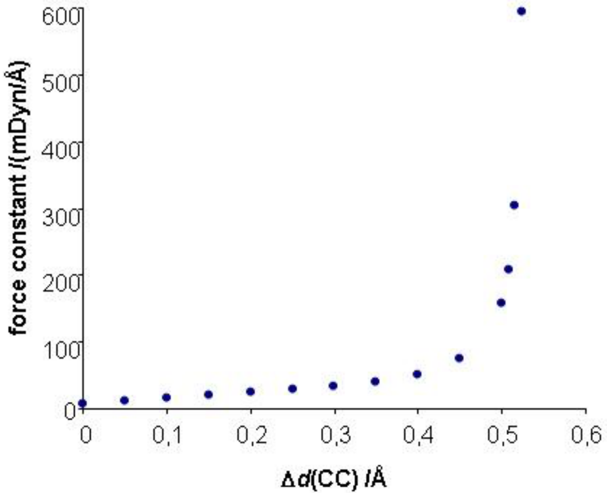

Figure S2. Dependence of the excitation energy for the lowest singlet excited state on a uniform CC bond elongation computed at BHHLYP/6-311+G(*d*,*p*).

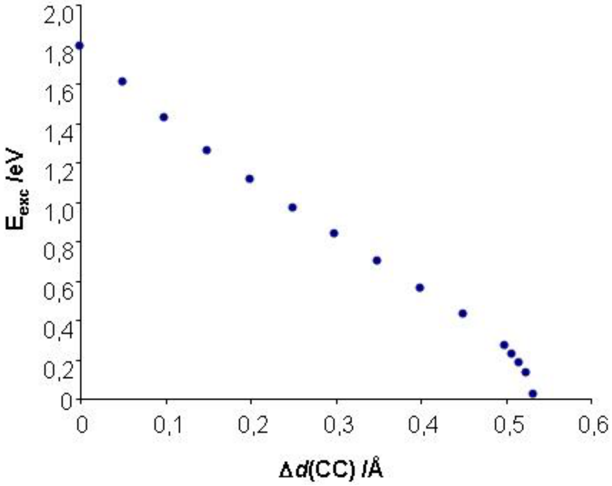

[3]**1** possesses at each BHHLYP/6-311+G(*d*,*p*) and KMLYP/6-311+G(d,p) three small imaginary frequencies: –229,8421; –165,8324; –161,083 cm^{-1} and -178,7145; –173,5409; –116,9523 cm^{-1}.

The single point energy of the X-ray structure of **1** determined 1965 at CCSD(T)/cc-pVDZ is some 130.2 kcal mol^{-1} higher than of the D_{6h} structure optimized at the same level of theory; geometry optimization starting with this structure breaks symmetry toward C_1 at CCSD/DZ.

TDBHLYP predicts the only strong UV/VIS transition at 347.45 nm for the D_{6h} symmetric while the experimental value is 369 nm (F. Sondheimer, R. Wolovsky, Y. Amiel, *J. Am. Chem. Soc.* **1962**, *84*, 274–284; M. Gouterman, G. Wagniere, *J. Chem Phys.* **1962**, *36*, 1188–1196; H. Baumann, J. F. M. Oth, *Helv. Chim. Acta* **1982**, *65*, 1885–1893).

Table S15. Total energies from stability analyses of **1**.

PG	E /h	ZPVE [kcal mol^{-1}]	Level
D_{6h}	-692.0749168	199.60472	HF/6-311G(*d*,*p*)
D_{3h}	-692.1103647	201.42522	HF/6-311G(*d*,*p*)
D_{3d}	-692.0620651	254.75286	HF/6-311G(*d*,*p*)
C_2	-692.1156477	201.87117	HF/6-311G(*d*,*p*)
D_{6h}	-691.7367184	203.18022	HF/DZ
D_{3h}	-691.7640828	204.63295	HF/DZ
C_2	-691.7667563	204.93268	HF/DZ
D_{6h}	-691.9813591	199.93700	HF/cc-pVDZ
D_{3h}	-692.0154723	201.74798	HF/cc-pVDZ
C_2	-692.0202203	202.07851	HF/cc-pVDZ
D_{6h}	-694.419031	916.03856	MP2/cc-pVDZ
D_{3h}	-694.3626414	185.24159	MP2/cc-pVDZ
D_{3d}	-694.46488483	1679.0944	MP2/cc-pVDZ
D_{6h}	-693.65710278	183.4968	CCSD/DZ
D_{3h}	-693.67656723	184.6288	CCSD/DZ
D_{6h}	-693.65504640		B-CCD/DZ
D_{6h}	-694.67570267		CCSD(T)/cc-pVDZ
D_{6h}	-649.0556888	148.62315	KMLYP/6-311+G(*d*,*p*)
D_{3h}	-649.0644417	149.88110	KMLYP/6-311+G(*d*,*p*)
D_{3d}	-649.0704434	149.87103	KMLYP/6-311+G(*d*,*p*)
C_2	-649.0556821	148.61967	KMLYP/6-311+G(*d*,*p*)
D_{6h}	-696.3401324	193.61570	BHLYP/6-311+G(*d*,*p*)
D_{3h}	-696.3435339	194.64061	BHLYP/6-311+G(*d*,*p*)
D_{3d}	-696.3436076	194.75803	BHLYP/6-311+G(*d*,*p*)
C_2	-696.3440912	195.20046	BHLYP/6-311+G(*d*,*p*)
D_{6h}	-696.540947	185.03216	OLYP/6-311+G(*d*,*p*)
D_{3h}	-696.5409876	185.13651	OLYP/6-311+G(*d*,*p*)
D_{3d}	-696.5407898	184.87437	OLYP/6-311+G(*d*,*p*)
C_2	-696.5407995	185.04477	OLYP/6-311+G(*d*,*p*)
D_{6h}	-696.8229456	187.89899	B3LYP/6-311+G(*d*,*p*)

Table S16. Total energies from stability analyses of **2**-**6**.

species	PG	E /h	ZPVE [kcal mol^{-1}]	Level
**2**	D_{6h}	-230.7568411	66.84426	HF/6-311++G(*d*,*p*)
**2**	D_{6h}	-231.5064538	63.07531	MP2/cc-pVDZ
**2**	D_{6h}	-231.598032597934	62.4270	MP4/cc-pVDZ
**2**	D_{6h}	-231.263033661308	61.5050	CCSD/DZ
**2**	D_{6h}	-231.596908824861	62.4046	CCSD(T)/cc-pVDZ
**2**	D_{6h}	-216.3967092	49.98774	KMLYP/6-311+G(*d*,*p*)
**2**	D_{6h}	-232.1587687	65.03302	BHLYP/6-311+G(*d*,*p*)
**2**	D_{6h}	-232.3112424	62.82834	B3LYP/6-311+G(*d*,*p*)
**3**	D_{6h}	-1832.1666225	374.54118	HF/6-311++G(*d*,*p*)
**3**	D_{6h}	-1838.1212258		MP2/cc-pVDZ
**3**	D_{6h}	-1830.6891662	302.06483	KMLYP/6-311+G(*d*,*p*)
**3**	D_{6h}	-1842.9991005	363.10762	BHLYP/6-311+G(*d*,*p*)
**3**	D_{6h}	-1844.1240272	349.68710	B3LYP/6-311+G(*d*,*p*)
**4**	D_{6h}	-916.1288892	185.86876	HF/6-311++G(*d*,*p*)
**4**	D_{6h}	-919.0955071	169.86125	MP2/cc-pVDZ
**4**	D_{6h}	-915.3925782	150.44394	KMLYP/6-311+G(*d*,*p*)
**4**	D_{6h}	-921.5395247	179.91302	BHLYP/6-311+G(*d*,*p*)
**4**	D_{6h}	-922.1006318	173.40439	B3LYP/6-311+G(*d*,*p*)
**5**	D_{2h}	-688.7808979	160.35586	HF/6-311++G(*d*,*p*)
**5**	D_{2h}	-688.6789499	148.59573	MP2/cc-pVDZ
**5**	D_{2h}	-688.2171819	131.63290	KMLYP/6-311+G(*d*,*p*)
**5**	D_{2h}	-692.8924007	155.78389	BHLYP/6-311+G(*d*,*p*)
**5**	D_{2h}	-693.3291833	150.26554	B3LYP/6-311+G(*d*,*p*)
**6**	C_{2v}	-805.680305	217.43707	HF/6-311++G(*d*,*p*)
**6**	C_{2v}	-808.3445206	202.65191	MP2/cc-pVDZ
**6**	C_{2v}	-804.988863	180.10215	KMLYP/6-311+G(*d*,*p*)
**6**	C_{2v}	-810.5523738	210.24949	BHLYP/6-311+G(*d*,*p*)
**6**	C_{2v}	-811.0707526	202.24542	B3LYP/6-311+G(*d*,*p*)

Table S17. Structures (Ångstroms) from stability analyses of **1**.

***D_{6h}*, HF/6-311G(*d*,*p*)**

1	0.000000000	0.000000000	1.889771000
1	1.636590000	0.000000000	0.944886000
1	1.636590000	0.000000000	-0.944886000
1	0.000000000	0.000000000	-1.889771000
1	-1.636590000	0.000000000	-0.944886000
1	-1.636590000	0.000000000	0.944886000
6	-1.247783000	0.000000000	3.562369000
6	1.247783000	0.000000000	3.562369000
6	2.461210000	0.000000000	2.861796000
6	3.708994000	0.000000000	0.700572000
6	3.708994000	0.000000000	-0.700572000
6	2.461210000	0.000000000	-2.861796000
6	1.247783000	0.000000000	-3.562369000
6	-1.247783000	0.000000000	-3.562369000
6	-2.461210000	0.000000000	-2.861796000
6	-3.708994000	0.000000000	-0.700572000
6	-3.708994000	0.000000000	0.700572000
6	-2.461210000	0.000000000	2.861796000
6	0.000000000	0.000000000	2.959170000
6	2.562716000	0.000000000	1.479585000
6	2.562716000	0.000000000	-1.479585000
6	0.000000000	0.000000000	-2.959170000
6	-2.562716000	0.000000000	-1.479585000
6	-2.562716000	0.000000000	1.479585000
1	1.296851000	0.000000000	4.638956000
1	3.369028000	0.000000000	3.442584000
1	4.665879000	0.000000000	1.196372000
1	4.665879000	0.000000000	-1.196372000
1	3.369028000	0.000000000	-3.442584000
1	1.296851000	0.000000000	-4.638956000
1	-1.296851000	0.000000000	-4.638956000
1	-3.369028000	0.000000000	-3.442584000
1	-4.665879000	0.000000000	-1.196372000
1	-4.665879000	0.000000000	1.196372000
1	-3.369028000	0.000000000	3.442584000
1	-1.296851000	0.000000000	4.638956000

***D_{6h}*, HF/DZ**

1	0.000000000	0.000000000	1.908609000
1	1.652904000	0.000000000	0.954305000
1	1.652904000	0.000000000	-0.954305000
1	0.000000000	0.000000000	-1.908609000
1	-1.652904000	0.000000000	-0.954305000
1	-1.652904000	0.000000000	0.954305000
6	-1.255996000	0.000000000	3.585466000
6	1.255996000	0.000000000	3.585466000
6	2.477107000	0.000000000	2.880457000
6	3.733103000	0.000000000	0.705009000
6	3.733103000	0.000000000	-0.705009000
6	2.477107000	0.000000000	-2.880457000
6	1.255996000	0.000000000	-3.585466000
6	-1.255996000	0.000000000	-3.585466000
6	-2.477107000	0.000000000	-2.880457000
6	-3.733103000	0.000000000	-0.705009000
6	-3.733103000	0.000000000	0.705009000
6	-2.477107000	0.000000000	2.880457000
6	0.000000000	0.000000000	2.977092000
6	2.578237000	0.000000000	1.488546000
6	2.578237000	0.000000000	-1.488546000
6	0.000000000	0.000000000	-2.977092000
6	-2.578237000	0.000000000	-1.488546000
6	-2.578237000	0.000000000	1.488546000
1	1.304979000	0.000000000	4.659865000
1	3.383072000	0.000000000	3.460078000
1	4.688051000	0.000000000	1.199787000
1	4.688051000	0.000000000	-1.199787000
1	3.383072000	0.000000000	-3.460078000
1	1.304979000	0.000000000	-4.659865000
1	-1.304979000	0.000000000	-4.659865000
1	-3.383072000	0.000000000	-3.460078000
1	-4.688051000	0.000000000	-1.199787000
1	-4.688051000	0.000000000	1.199787000
1	-3.383072000	0.000000000	3.460078000
1	-1.304979000	0.000000000	4.659865000

***D_{6h}*, HF/cc-pVDZ**

1	0.000000000	0.000000000	1.889234000

***D_{3h}*, HF/6-311G(*d*,*p*)**

1	0.960360000	1.642267000	0.000000000
1	-0.960360000	1.642267000	0.000000000
1	-1.902424000	0.010562000	0.000000000
1	-0.942065000	-1.652829000	0.000000000
1	0.942065000	-1.652829000	0.000000000
1	1.902424000	0.010562000	0.000000000
6	2.935602000	2.469251000	0.000000000
6	0.738545000	3.680787000	0.000000000
6	-0.738545000	3.680787000	0.000000000
6	-2.935602000	2.469251000	0.000000000
6	-3.606235000	1.307680000	0.000000000
6	-3.556927000	-1.200795000	0.000000000
6	-2.818382000	-2.479992000	0.000000000
6	-0.670633000	-3.776931000	0.000000000
6	0.670633000	-3.776931000	0.000000000
6	2.818382000	-2.479992000	0.000000000
6	3.556927000	-1.200795000	0.000000000
6	3.606235000	1.307680000	0.000000000
6	1.482559000	2.575518000	0.000000000
6	-1.482559000	2.575518000	0.000000000
6	-2.971743000	-0.003826000	0.000000000
6	-1.489185000	-2.571692000	0.000000000
6	1.489185000	-2.571692000	0.000000000
6	2.971743000	-0.003826000	0.000000000
1	1.216669000	4.647034000	0.000000000
1	-1.216669000	4.647034000	0.000000000
1	-3.497569000	3.388434000	0.000000000
1	-4.683254000	1.334767000	0.000000000
1	-4.632784000	-1.269851000	0.000000000
1	-3.416116000	-3.377183000	0.000000000
1	-1.185685000	-4.723200000	0.000000000
1	1.185685000	-4.723200000	0.000000000
1	3.416116000	-3.377183000	0.000000000
1	4.632784000	-1.269851000	0.000000000
1	4.683254000	1.334767000	0.000000000
1	3.497569000	3.388434000	0.000000000

***D_{3h}*, HF/DZ**

1	0.969442000	1.653759000	0.000000000
1	-0.969442000	1.653759000	0.000000000
1	-1.916918000	0.012682000	0.000000000
1	-0.947476000	-1.666441000	0.000000000
1	0.947476000	-1.666441000	0.000000000
1	1.916918000	0.012682000	0.000000000
6	2.945895000	2.483365000	0.000000000
6	0.738684000	3.704197000	0.000000000
6	-0.738684000	3.704197000	0.000000000
6	-2.945895000	2.483365000	0.000000000
6	-3.623605000	1.309537000	0.000000000
6	-3.577271000	-1.212379000	0.000000000
6	-2.838586000	-2.491818000	0.000000000
6	-0.677710000	-3.792902000	0.000000000
6	0.677710000	-3.792902000	0.000000000
6	2.838586000	-2.491818000	0.000000000
6	3.577271000	-1.212379000	0.000000000
6	3.623605000	1.309537000	0.000000000
6	1.490413000	2.586816000	0.000000000
6	-1.490413000	2.586816000	0.000000000
6	-2.985455000	-0.002673000	0.000000000
6	-1.495042000	-2.584143000	0.000000000
6	1.495042000	-2.584143000	0.000000000
6	2.985455000	-0.002673000	0.000000000
1	1.215107000	4.668780000	0.000000000
1	-1.215107000	4.668780000	0.000000000
1	-3.505657000	3.401459000	0.000000000
1	-4.698579000	1.335258000	0.000000000
1	-4.650836000	-1.282076000	0.000000000
1	-3.435728000	-3.386704000	0.000000000
1	-1.192922000	-4.736718000	0.000000000
1	1.192922000	-4.736718000	0.000000000
1	3.435728000	-3.386704000	0.000000000
1	4.650836000	-1.282076000	0.000000000
1	4.698579000	1.335258000	0.000000000
1	3.505657000	3.401459000	0.000000000

***D_{3h}*, HF/cc-pVDZ**

1	0.960102000	1.641720000	0.000000000

1	1.636125000	0.000000000	0.944617000
1	1.636125000	0.000000000	-0.944617000
1	0.000000000	0.000000000	-1.889234000
1	-1.636125000	0.000000000	-0.944617000
1	-1.636125000	0.000000000	0.944617000
6	-1.250575000	0.000000000	3.570011000
6	1.250575000	0.000000000	3.570011000
6	2.466433000	0.000000000	2.868035000
6	3.717008000	0.000000000	0.701976000
6	3.717008000	0.000000000	-0.701976000
6	2.466433000	0.000000000	-2.868035000
6	1.250575000	0.000000000	-3.570011000
6	-1.250575000	0.000000000	-3.570011000
6	-2.466433000	0.000000000	-2.868035000
6	-3.717008000	0.000000000	-0.701976000
6	-3.717008000	0.000000000	0.701976000
6	-2.466433000	0.000000000	2.868035000
6	0.000000000	0.000000000	2.965166000
6	2.567909000	0.000000000	1.482583000
6	2.567909000	0.000000000	-1.482583000
6	0.000000000	0.000000000	-2.965166000
6	-2.567909000	0.000000000	-1.482583000
6	-2.567909000	0.000000000	1.482583000
1	1.299851000	0.000000000	4.653142000
1	3.379813000	0.000000000	3.452275000
1	4.679664000	0.000000000	1.200867000
1	4.679664000	0.000000000	-1.200867000
1	3.379813000	0.000000000	-3.452275000
1	1.299851000	0.000000000	-4.653142000
1	-1.299851000	0.000000000	-4.653142000
1	-3.379813000	0.000000000	-3.452275000
1	-4.679664000	0.000000000	-1.200867000
1	-4.679664000	0.000000000	1.200867000
1	-3.379813000	0.000000000	3.452275000
1	-1.299851000	0.000000000	4.653142000

D_{6h}, MP2/cc-pVDZ

1	0.000000000	0.000000000	1.888654000
1	1.635622000	0.000000000	0.944327000
1	1.635622000	0.000000000	-0.944327000
1	0.000000000	0.000000000	-1.888654000
1	-1.635622000	0.000000000	-0.944327000
1	-1.635622000	0.000000000	0.944327000
6	-1.260352000	0.000000000	3.602308000
6	1.260352000	0.000000000	3.602308000
6	2.489514000	0.000000000	2.892651000
6	3.749867000	0.000000000	0.709657000
6	3.749867000	0.000000000	-0.709657000
6	2.489514000	0.000000000	-2.892651000
6	1.260352000	0.000000000	-3.602308000
6	-1.260352000	0.000000000	-3.602308000
6	-2.489514000	0.000000000	-2.892651000
6	-3.749867000	0.000000000	-0.709657000
6	-3.749867000	0.000000000	0.709657000
6	-2.489514000	0.000000000	2.892651000
6	0.000000000	0.000000000	2.984212000
6	2.584403000	0.000000000	1.492106000
6	2.584403000	0.000000000	-1.492106000
6	0.000000000	0.000000000	-2.984212000
6	-2.584403000	0.000000000	-1.492106000
6	-2.584403000	0.000000000	1.492106000
1	1.301349000	0.000000000	4.700647000
1	3.420205000	0.000000000	3.477325000
1	4.721554000	0.000000000	1.223322000
1	4.721554000	0.000000000	-1.223322000
1	3.420205000	0.000000000	-3.477325000
1	1.301349000	0.000000000	-4.700647000
1	-1.301349000	0.000000000	-4.700647000
1	-3.420205000	0.000000000	-3.477325000
1	-4.721554000	0.000000000	-1.223322000
1	-4.721554000	0.000000000	1.223322000
1	-3.420205000	0.000000000	3.477325000
1	-1.301349000	0.000000000	4.700647000

D_{6h}, CCSD/DZ

1	0.000000000	-1.929516397	0.000000000
1	1.671010215	-0.964758198	0.000000000
1	1.671010215	0.964758198	0.000000000
1	0.000000000	1.929516397	0.000000000
1	-1.671010215	0.964758198	0.000000000
1	-1.671010215	-0.964758198	0.000000000

1	-0.960102000	1.641720000	0.000000000
1	-1.901822000	0.010613000	0.000000000
1	-0.941720000	-1.652333000	0.000000000
1	0.941720000	-1.652333000	0.000000000
1	1.901822000	0.010613000	0.000000000
6	2.939863000	2.474383000	0.000000000
6	0.738853000	3.689551000	0.000000000
6	-0.738853000	3.689551000	0.000000000
6	-2.939863000	2.474383000	0.000000000
6	-3.612810000	1.308805000	0.000000000
6	-3.564671000	-1.204910000	0.000000000
6	-2.825818000	-2.484641000	0.000000000
6	-0.672947000	-3.783188000	0.000000000
6	0.672947000	-3.783188000	0.000000000
6	2.825818000	-2.484641000	0.000000000
6	3.564671000	-1.204910000	0.000000000
6	3.612810000	1.308805000	0.000000000
6	1.485792000	2.580456000	0.000000000
6	-1.485792000	2.580456000	0.000000000
6	-2.977636000	-0.003494000	0.000000000
6	-1.491844000	-2.576961000	0.000000000
6	1.491844000	-2.576961000	0.000000000
6	2.977636000	-0.003494000	0.000000000
1	1.220374000	4.661364000	0.000000000
1	-1.220374000	4.661364000	0.000000000
1	-3.505859000	3.398754000	0.000000000
1	-4.696337000	1.336786000	0.000000000
1	-4.647047000	-1.273807000	0.000000000
1	-3.426673000	-3.387557000	0.000000000
1	-1.190478000	-4.735540000	0.000000000
1	1.190478000	-4.735540000	0.000000000
1	3.426673000	-3.387557000	0.000000000
1	4.647047000	-1.273807000	0.000000000
1	4.696337000	1.336786000	0.000000000
1	3.505859000	3.398754000	0.000000000

D_{3h}, MP2/cc-pVDZ

6	2.580733000	-1.490437000	0.000000000
6	2.580733000	1.490437000	0.000000000
6	-2.581123000	-1.489762000	0.000000000
6	0.000390000	-2.980199000	0.000000000
6	-2.581123000	1.489762000	0.000000000
6	0.000390000	2.980199000	0.000000000
6	3.744874000	-0.708182000	0.000000000
6	3.744874000	0.708182000	0.000000000
6	-2.485740000	-2.889065000	0.000000000
6	-1.259134000	-3.597247000	0.000000000
6	-2.485740000	2.889065000	0.000000000
6	-1.259134000	3.597247000	0.000000000
1	4.714693000	-1.223357000	0.000000000
1	4.714693000	1.223357000	0.000000000
1	-3.416805000	-3.471366000	0.000000000
1	-1.297888000	-4.694723000	0.000000000
1	-3.416805000	3.471366000	0.000000000
1	-1.297888000	4.694723000	0.000000000
1	1.629818000	-0.947274000	0.000000000
1	1.629818000	0.947274000	0.000000000
1	-1.635273000	-0.937826000	0.000000000
1	0.005455000	-1.885101000	0.000000000
1	-1.635273000	0.937826000	0.000000000
1	0.005455000	1.885101000	0.000000000
6	-3.747513000	-0.694155000	0.000000000
6	-3.747513000	0.694155000	0.000000000
6	2.474912000	-2.898364000	0.000000000
6	1.272601000	-3.592519000	0.000000000
6	2.474912000	2.898364000	0.000000000
6	1.272601000	3.592519000	0.000000000
1	-4.768574000	-0.992820000	0.000000000
1	-4.768574000	0.992820000	0.000000000
1	3.244094000	-3.633296000	0.000000000
1	1.524480000	-4.626116000	0.000000000
1	3.244094000	3.633296000	0.000000000
1	1.524480000	4.626116000	0.000000000

D_{3h}, CCSD/DZ

6	-3.031757300	0.000000000	-0.001696966
6	-1.514409035	0.000000000	2.626427323
6	1.517348265	0.000000000	-2.624730358
6	-1.517348265	0.000000000	-2.624730358
6	3.031757300	0.000000000	-0.001696966
6	1.514409035	0.000000000	2.626427323

6	-1.277816580	-3.650752549	0.000000000
6	1.277816580	-3.650752549	0.000000000
6	2.522736163	-2.931997895	0.000000000
6	3.800552743	-0.718754654	0.000000000
6	3.800552743	0.718754654	0.000000000
6	2.522736163	2.931997895	0.000000000
6	1.277816580	3.650752549	0.000000000
6	-1.277816580	3.650752549	0.000000000
6	-2.522736163	2.931997895	0.000000000
6	-3.800552743	0.718754654	0.000000000
6	-3.800552743	-0.718754654	0.000000000
6	-2.522736163	-2.931997895	0.000000000
6	0.000000000	-3.024101978	0.000000000
6	2.618949133	-1.512050989	0.000000000
6	2.618949133	1.512050989	0.000000000
6	0.000000000	3.024101978	0.000000000
6	-2.618949133	1.512050989	0.000000000
6	-2.618949133	-1.512050989	0.000000000
1	1.319692043	-4.749521081	0.000000000
1	3.453359889	-3.517647373	0.000000000
1	4.773051932	-1.231873702	0.000000000
1	4.773051932	1.231873702	0.000000000
1	3.453359889	3.517647373	0.000000000
1	1.319692043	4.749521081	0.000000000
1	-1.319692043	4.749521081	0.000000000
1	-3.453359889	3.517647373	0.000000000
1	-4.773051932	1.231873702	0.000000000
1	-4.773051932	-1.231873702	0.000000000
1	-3.453359889	-3.517647373	0.000000000
1	-1.319692043	-4.749521081	0.000000000

D_{6h}, B-CCD/DZ

1	0.000000000	-1.929453726	0.000000000
1	1.670955942	-0.964726866	0.000000000
1	1.670955942	0.964726866	0.000000000
1	0.000000000	1.929453726	0.000000000
1	-1.670955942	0.964726866	0.000000000
1	-1.670955942	-0.964726866	0.000000000
6	-1.277720439	-3.650410748	0.000000000
6	1.277720439	-3.650410748	0.000000000
6	2.522488223	-2.931743736	0.000000000
6	3.800208662	-0.718667017	0.000000000
6	3.800208662	0.718667017	0.000000000
6	2.522488223	2.931743736	0.000000000
6	1.277720439	3.650410748	0.000000000
6	-1.277720439	3.650410748	0.000000000
6	-2.522488223	2.931743736	0.000000000
6	-3.800208662	0.718667017	0.000000000
6	-3.800208662	-0.718667017	0.000000000
6	-2.522488223	-2.931743736	0.000000000
6	0.000000000	-3.023909918	0.000000000
6	2.618782808	-1.511954959	0.000000000
6	2.618782808	1.511954959	0.000000000
6	0.000000000	3.023909918	0.000000000
6	-2.618782808	1.511954959	0.000000000
6	-2.618782808	-1.511954959	0.000000000
1	1.319746193	-4.749096702	0.000000000
1	3.452965292	-3.517482080	0.000000000
1	4.772711485	-1.231614622	0.000000000
1	4.772711485	1.231614622	0.000000000
1	3.452965292	3.517482080	0.000000000
1	1.319746193	4.749096702	0.000000000
1	-1.319746193	4.749096702	0.000000000
1	-3.452965292	3.517482080	0.000000000
1	-4.772711485	1.231614622	0.000000000
1	-4.772711485	-1.231614622	0.000000000
1	-3.452965292	-3.517482080	0.000000000
1	-1.319746193	-4.749096702	0.000000000

D_{6h}, CCSD(T)/cc-pVDZ

1	0.000000000	-1.894939098	0.000000000
1	1.641065397	-0.947469549	0.000000000
1	1.641065397	0.947469549	0.000000000
1	0.000000000	1.894939098	0.000000000
1	-1.641065397	0.947469549	0.000000000
1	-1.641065397	-0.947469549	0.000000000
6	-1.263706841	-3.612396062	0.000000000
6	1.263706841	-3.612396062	0.000000000
6	2.496573338	-2.900600258	0.000000000
6	3.760280174	-0.711795804	0.000000000
6	3.760280174	0.711795804	0.000000000

6	-3.686091921	0.000000000	1.326343917
6	-2.991693486	0.000000000	2.529077284
6	0.694398436	0.000000000	-3.855421201
6	-0.694398436	0.000000000	-3.855421201
6	3.686091921	0.000000000	1.326343917
6	2.991693486	0.000000000	2.529077284
1	-4.785399865	0.000000000	1.346235288
1	-3.558573889	0.000000000	3.471160210
1	1.226825976	0.000000000	-4.817395493
1	-1.226825976	0.000000000	-4.817395493
1	4.785399865	0.000000000	1.346235288
1	3.558573889	0.000000000	3.471160210
1	-1.937415637	0.000000000	0.010750051
1	-0.978017634	0.000000000	1.672476136
1	0.959398003	0.000000000	-1.683226182
1	-0.959398003	0.000000000	-1.683226182
1	1.937415637	0.000000000	0.010750051
1	0.978017634	0.000000000	1.672476136
6	2.894883884	0.000000000	-2.536882368
6	3.644446519	0.000000000	-1.238601805
6	-3.644446519	0.000000000	-1.238601805
6	-2.894883884	0.000000000	-2.536882368
6	-0.749562634	0.000000000	3.775484172
6	0.749562634	0.000000000	3.775484172
1	3.496319562	0.000000000	-3.457531245
1	4.742469672	0.000000000	-1.299135935
1	-4.742469672	0.000000000	-1.299135935
1	-3.496319562	0.000000000	-3.457531245
1	-1.246150116	0.000000000	4.756667180
1	1.246150116	0.000000000	4.756667180

D_{3h}, KMLYP/6-311+G(*d*,*p*)

6	1.667208000	2.882034000	0.000000000
6	3.329518000	0.002828000	0.000000000
6	-3.329518000	0.002828000	0.000000000
6	-1.667208000	2.882034000	0.000000000
6	-1.662310000	-2.884861000	0.000000000
6	1.662310000	-2.884861000	0.000000000
6	3.155713000	2.771597000	0.000000000
6	3.978130000	1.347128000	0.000000000
6	-3.978130000	1.347129000	0.000000000
6	-3.155712000	2.771597000	0.000000000
6	-0.822417000	-4.118726000	0.000000000
6	0.822417000	-4.118726000	0.000000000
1	3.842068000	3.787010000	0.000000000
1	5.200681000	1.433823000	0.000000000
1	-5.200681000	1.433823000	0.000000000
1	-3.842068000	3.787010000	0.000000000
1	-1.358613000	-5.220833000	0.000000000
1	1.358613000	-5.220833000	0.000000000
1	1.047817000	1.839121000	0.000000000
1	2.116634000	-0.012125000	0.000000000
1	-2.116634000	-0.012124000	0.000000000
1	-1.047817000	1.839121000	0.000000000
1	-1.068817000	-1.826997000	0.000000000
1	1.068817000	-1.826997000	0.000000000
6	-4.025628000	-1.453523000	0.000000000
6	-3.271602000	-2.759535000	0.000000000
6	0.754027000	4.213058000	0.000000000
6	-0.754026000	4.213058000	0.000000000
6	4.025628000	-1.453523000	0.000000000
6	3.271602000	-2.759535000	0.000000000
1	-5.249852000	-1.490941000	0.000000000
1	-3.916119000	-3.801035000	0.000000000
1	1.333733000	5.291976000	0.000000000
1	-1.333733000	5.291976000	0.000000000
1	5.249852000	-1.490941000	0.000000000
1	3.916118000	-3.801035000	0.000000000

D_{3h}, BHLYP/6-311+G(*d*,*p*)

6	1.480716000	2.562101000	0.000000000
6	2.959203000	0.001287000	0.000000000
6	-2.959203000	0.001287000	0.000000000
6	-1.480716000	2.562101000	0.000000000
6	-1.478487000	-2.563388000	0.000000000
6	1.478486000	-2.563388000	0.000000000
6	2.827128000	2.465951000	0.000000000
6	3.549140000	1.215389000	0.000000000
6	-3.549140000	1.215389000	0.000000000
6	-2.827128000	2.465951000	0.000000000
6	-0.722013000	-3.681340000	0.000000000

6	2.496573338	2.900600258	0.000000000
6	1.263706841	3.612396062	0.000000000
6	-1.263706841	3.612396062	0.000000000
6	-2.496573338	2.900600258	0.000000000
6	-3.760280174	0.711795804	0.000000000
6	-3.760280174	-0.711795804	0.000000000
6	-2.496573338	-2.900600258	0.000000000
6	0.000000000	-2.990245678	0.000000000
6	2.589628724	-1.495122842	0.000000000
6	2.589628724	1.495122842	0.000000000
6	0.000000000	2.990245678	0.000000000
6	-2.589628724	1.495122842	0.000000000
6	-2.589628724	-1.495122842	0.000000000
1	1.304992806	-4.711312796	0.000000000
1	3.427620162	-3.485813319	0.000000000
1	4.732612968	-1.225499477	0.000000000
1	4.732612968	1.225499477	0.000000000
1	3.427620162	3.485813319	0.000000000
1	1.304992806	4.711312796	0.000000000
1	-1.304992806	4.711312796	0.000000000
1	-3.427620162	3.485813319	0.000000000
1	-4.732612968	1.225499477	0.000000000
1	-4.732612968	-1.225499477	0.000000000
1	-3.427620162	-3.485813319	0.000000000

D_{6h}, KMLYP/6-311+G(*d*,*p*)

1	0.000000000	0.000000000	2.107962000
1	1.825549000	0.000000000	1.053981000
1	1.825549000	0.000000000	-1.053981000
1	0.000000000	0.000000000	-2.107962000
1	-1.825549000	0.000000000	-1.053981000
1	-1.825549000	0.000000000	1.053981000
6	-1.395207000	0.000000000	3.986124000
6	1.395207000	0.000000000	3.986124000
6	2.754481000	0.000000000	3.201347000
6	4.149688000	0.000000000	0.784777000
6	4.149688000	0.000000000	-0.784777000
6	2.754481000	0.000000000	-3.201347000
6	1.395207000	0.000000000	-3.986124000
6	-1.395207000	0.000000000	-3.986124000
6	-2.754481000	0.000000000	-3.201347000
6	-4.149688000	0.000000000	-0.784777000
6	-4.149688000	0.000000000	0.784777000
6	-2.754481000	0.000000000	3.201347000
6	0.000000000	0.000000000	3.321065000
6	2.876126000	0.000000000	1.660532000
6	2.876126000	0.000000000	-1.660532000
6	0.000000000	0.000000000	-3.321065000
6	-2.876126000	0.000000000	-1.660532000
6	-2.876126000	0.000000000	1.660532000
1	1.458845000	0.000000000	5.209741000
1	3.782346000	0.000000000	3.868267000
1	5.241190000	0.000000000	1.341474000
1	5.241190000	0.000000000	-1.341474000
1	3.782346000	0.000000000	-3.868267000
1	1.458845000	0.000000000	-5.209741000
1	-1.458845000	0.000000000	-5.209741000
1	-3.782346000	0.000000000	-3.868267000
1	-5.241190000	0.000000000	-1.341474000
1	-5.241190000	0.000000000	1.341474000
1	-3.782346000	0.000000000	3.868267000
1	-1.458845000	0.000000000	5.209741000

D_{6h}, BHLYP/6-311+G(*d*,*p*)

1	0.000000000	0.000000000	1.882557000
1	1.630342000	0.000000000	0.941278000
1	1.630342000	0.000000000	-0.941278000
1	0.000000000	0.000000000	-1.882557000
1	-1.630342000	0.000000000	-0.941278000
1	-1.630342000	0.000000000	0.941278000
6	-1.245912000	0.000000000	3.558212000
6	1.245912000	0.000000000	3.558212000
6	2.458546000	0.000000000	2.858098000
6	3.704458000	0.000000000	0.700114000
6	3.704458000	0.000000000	-0.700114000
6	2.458546000	0.000000000	-2.858098000
6	1.245912000	0.000000000	-3.558212000
6	-1.245912000	0.000000000	-3.558212000
6	-2.458546000	0.000000000	-2.858098000
6	-3.704458000	0.000000000	-0.700114000

6	0.722013000	-3.681340000	0.000000000
1	3.417408000	3.369390000	0.000000000
1	4.626681000	1.274867000	0.000000000
1	-4.626681000	1.274867000	0.000000000
1	-3.417408000	3.369390000	0.000000000
1	-1.209274000	-4.644257000	0.000000000
1	1.209273000	-4.644257000	0.000000000
1	0.936002000	1.637148000	0.000000000
1	1.885813000	-0.007973000	0.000000000
1	-1.885813000	-0.007972000	0.000000000
1	-0.936002000	1.637148000	0.000000000
1	-0.949811000	-1.629175000	0.000000000
1	0.949811000	-1.629176000	0.000000000
6	-3.579943000	-1.280677000	0.000000000
6	-2.899071000	-2.459983000	0.000000000
6	0.680873000	3.740660000	0.000000000
6	-0.680872000	3.740660000	0.000000000
6	3.579943000	-1.280677000	0.000000000
6	2.899071000	-2.459983000	0.000000000
1	-4.658285000	-1.314020000	0.000000000
1	-3.467117000	-3.377182000	0.000000000
1	1.191167000	4.691203000	0.000000000
1	-1.191167000	4.691203000	0.000000000
1	4.658285000	-1.314021000	0.000000000
1	3.467117000	-3.377183000	0.000000000

C_2, HF/6-311G(*d*,*p*)

1	0.485755000	1.003032000	-1.717585000
1	0.468454000	2.024643000	0.017563000
1	-0.466905000	0.926281000	1.664136000
1	0.466905000	-0.926281000	1.664136000
1	-0.468454000	-2.024643000	0.017563000
1	-0.485755000	-1.003032000	-1.717585000
6	-0.116017000	0.727666000	-3.661783000
6	-0.237240000	2.906444000	-2.456058000
6	-0.276099000	3.574809000	-1.299276000
6	-0.329785000	3.513573000	1.189869000
6	-0.170468000	2.795179000	2.466915000
6	-0.079171000	0.663376000	3.770940000
6	0.079171000	-0.663376000	3.770940000
6	0.170468000	-2.795179000	2.466915000
6	0.329785000	-3.513573000	1.189869000
6	0.276099000	-3.574809000	-1.299276000
6	0.237240000	-2.906444000	-2.456058000
6	0.116017000	-0.727666000	-3.661783000
6	0.079171000	1.485984000	-2.585443000
6	-0.006058000	2.987238000	0.011205000
6	-0.239163000	1.469387000	2.561537000
6	0.239163000	-1.469387000	2.561537000
6	0.006058000	-2.987238000	0.011205000
6	-0.079171000	-1.485984000	-2.585443000
1	-0.504136000	3.429117000	-3.359671000
1	-0.576436000	4.609492000	-1.314750000
1	-0.754830000	4.504165000	1.237446000
1	-0.013393000	3.392167000	3.351702000
1	-0.120846000	1.180772000	4.715072000
1	0.120846000	-1.180772000	4.715072000
1	0.013393000	-3.392167000	3.351702000
1	0.754830000	-4.504165000	1.237446000
1	0.576436000	-4.609492000	-1.314750000
1	0.504136000	-3.429117000	-3.359671000
1	0.463117000	-1.172850000	-4.581356000
1	-0.463117000	1.172850000	-4.581356000

C_2, HF/DZ

1	0.308577000	1.042382000	-1.717740000
1	0.155469000	2.028248000	0.005726000
1	-0.516701000	0.883334000	1.680686000
1	0.516701000	-0.883334000	1.680686000
1	-0.155469000	-2.028248000	0.005726000
1	-0.308577000	-1.042382000	-1.717740000
6	-0.183851000	0.714645000	-3.696625000
6	-0.548573000	2.877913000	-2.474281000
6	-0.659503000	3.543475000	-1.303135000
6	-0.676911000	3.491904000	1.205553000
6	-0.458762000	2.787456000	2.484025000
6	-0.140259000	0.660917000	3.791761000
6	0.140259000	-0.660917000	3.791761000
6	0.458762000	-2.787456000	2.484025000
6	0.676911000	-3.491904000	1.205553000
6	0.659503000	-3.543475000	-1.303135000

6	-3.704458000	0.000000000	0.700114000
6	-2.458546000	0.000000000	2.858098000
6	0.000000000	0.000000000	2.956031000
6	2.559998000	0.000000000	1.478016000
6	2.559998000	0.000000000	-1.478016000
6	0.000000000	0.000000000	-2.956031000
6	-2.559998000	0.000000000	-1.478016000
6	-2.559998000	0.000000000	1.478016000
1	1.293024000	0.000000000	4.636242000
1	3.368592000	0.000000000	3.437913000
1	4.661616000	0.000000000	1.198329000
1	4.661616000	0.000000000	-1.198329000
1	3.368592000	0.000000000	-3.437913000
1	1.293024000	0.000000000	-4.636242000
1	-1.293024000	0.000000000	-4.636242000
1	-3.368592000	0.000000000	-3.437913000
1	-4.661616000	0.000000000	-1.198329000
1	-4.661616000	0.000000000	1.198329000
1	-3.368592000	0.000000000	3.437913000
1	-1.293024000	0.000000000	4.636242000

D_{3d}, HF/6-311G(*d*,*p*)

6	0.000000000	-2.982202000	0.036329000
6	-2.582663000	1.491101000	0.036329000
6	2.582663000	1.491101000	0.036329000
6	0.000000000	2.982202000	-0.036329000
6	2.582663000	-1.491101000	-0.036329000
6	-2.582663000	-1.491101000	-0.036329000
6	-1.259622000	-3.600406000	-0.007228000
6	1.259622000	-3.600406000	-0.007228000
6	-2.488232000	2.891068000	-0.007228000
6	-3.747854000	0.709338000	-0.007228000
6	3.747854000	0.709338000	-0.007228000
6	2.488232000	2.891068000	-0.007228000
6	-1.259622000	3.600406000	0.007228000
6	1.259622000	3.600406000	0.007228000
6	3.747854000	-0.709338000	0.007228000
6	2.488232000	-2.891068000	0.007228000
6	-2.488232000	-2.891068000	0.007228000
6	-3.747854000	-0.709338000	0.007228000
1	0.000000000	-1.890230000	0.131447000
1	-1.636988000	0.945115000	0.131447000
1	1.636988000	0.945115000	0.131447000
1	0.000000000	1.890230000	-0.131447000
1	1.636988000	-0.945115000	-0.131447000
1	-1.636988000	-0.945115000	-0.131447000
1	-1.299619000	-4.698281000	-0.042250000
1	1.299619000	-4.698281000	-0.042250000
1	-3.419021000	3.474644000	-0.042250000
1	-4.718640000	1.223637000	-0.042250000
1	4.718640000	1.223637000	-0.042250000
1	3.419021000	3.474644000	-0.042250000
1	-1.299619000	4.698281000	0.042250000
1	1.299619000	4.698281000	0.042250000
1	4.718640000	-1.223637000	0.042250000
1	3.419021000	-3.474644000	0.042250000
1	-3.419021000	-3.474644000	0.042250000
1	-4.718640000	-1.223637000	0.042250000

D_{3d}, MP2/cc-pVDZ

6	-1.489998342	0.035429645	-2.580752834
6	2.979996684	0.035429645	0.000000000
6	-1.489998342	0.035429645	2.580752834
6	1.489998342	-0.035429645	2.580752834
6	-2.979996684	-0.035429645	0.000000000
6	1.489998342	-0.035429645	-2.580752834
6	-0.708720713	-0.007179977	-3.744775641
6	-2.888710481	-0.007179977	-2.486157961
6	3.597431194	-0.007179977	1.258617680
6	3.597431194	-0.007179977	-1.258617680
6	-2.888710481	-0.007179977	2.486157961
6	-0.708720713	-0.007179977	3.744775641
6	2.888710481	0.007179977	2.486157961
6	0.708720713	0.007179977	3.744775641
6	-3.597431194	0.007179977	1.258617680
6	-3.597431194	0.007179977	-1.258617680
6	0.708720713	0.007179977	-3.744775641
6	2.888710481	0.007179977	-2.486157961
1	-0.944457573	0.129242849	-1.635848503
1	1.888915145	0.129242849	0.000000000
1	-0.944457573	0.129242849	1.635848503

6	0.548573000	-2.877913000	-2.474281000
6	0.183851000	-0.714645000	-3.696625000
6	-0.106166000	1.488978000	-2.598717000
6	-0.355002000	2.969968000	0.007804000
6	-0.370281000	1.448147000	2.579350000
6	0.370281000	-1.448147000	2.579350000
6	0.355002000	-2.969968000	0.007804000
6	0.106166000	-1.488978000	-2.598717000
1	-0.838805000	3.379909000	-3.379947000
1	-1.035630000	4.550859000	-1.318476000
1	-1.137094000	4.463434000	1.261223000
1	-0.387711000	3.395807000	3.369139000
1	-0.228901000	1.172076000	4.733684000
1	0.228901000	-1.172076000	4.733684000
1	0.387711000	-3.395807000	3.369139000
1	1.137094000	-4.463434000	1.261223000
1	1.035630000	-4.550859000	-1.318476000
1	0.838805000	-3.379909000	-3.379947000
1	0.530854000	-1.130512000	-4.626930000
1	-0.530854000	1.130512000	-4.626930000

C_2, HF/cc-pVDZ

1	0.373798000	1.045814000	-1.714365000
1	0.251893000	2.057200000	0.015582000
1	-0.543895000	0.876023000	1.661110000
1	0.543895000	-0.876023000	1.661110000
1	-0.251893000	-2.057200000	0.015582000
1	-0.373798000	-1.045814000	-1.714365000
6	-0.188055000	0.712897000	-3.671309000
6	-0.532797000	2.872239000	-2.461855000
6	-0.639606000	3.535943000	-1.301089000
6	-0.683399000	3.472405000	1.194183000
6	-0.455312000	2.772437000	2.472050000
6	-0.144320000	0.654832000	3.776399000
6	0.144320000	-0.654832000	3.776399000
6	0.455312000	-2.772437000	2.472050000
6	0.683399000	-3.472405000	1.194183000
6	0.639606000	-3.535943000	-1.301089000
6	0.532797000	-2.872239000	-2.461855000
6	0.188055000	-0.712897000	-3.671309000
6	-0.075701000	1.489504000	-2.590169000
6	-0.313308000	2.977215000	0.010151000
6	-0.381475000	1.441993000	2.566373000
6	0.381475000	-1.441993000	2.566373000
6	0.313308000	-2.977215000	0.010151000
6	0.075701000	-1.489504000	-2.590169000
1	-0.852121000	3.369026000	-3.370952000
1	-1.044654000	4.541411000	-1.317815000
1	-1.201710000	4.425040000	1.242550000
1	-0.369024000	3.384930000	3.364008000
1	-0.236080000	1.168035000	4.726843000
1	0.236080000	-1.168035000	4.726843000
1	0.369024000	-3.384930000	3.364008000
1	1.201710000	-4.425040000	1.242550000
1	1.044654000	-4.541411000	-1.317815000
1	0.852121000	-3.369026000	-3.370952000
1	0.573735000	-1.125274000	-4.598592000
1	-0.573735000	1.125274000	-4.598592000

C_2, KMLYP/6-311+G(*d*,*p*)

1	0.038987000	1.826154000	-1.046653000
1	-0.006033000	1.825526000	1.061797000
1	0.000000000	0.000000000	2.115439000
1	0.006033000	-1.825526000	1.061797000
1	-0.038987000	-1.826154000	-1.046653000
1	0.000000000	0.000000000	-2.100205000
6	-0.000689000	2.754407000	-3.193936000
6	-0.005680000	4.149731000	-0.777476000
6	-0.001774000	4.149919000	0.792115000
6	0.002910000	2.754372000	3.209144000
6	0.001034000	1.395733000	3.993370000
6	-0.001034000	-1.395733000	3.993370000
6	-0.002910000	-2.754372000	3.209144000
6	0.001774000	-4.149919000	0.792115000
6	0.005680000	-4.149731000	-0.777476000
6	0.000689000	-2.754407000	-3.193936000
6	0.000099000	-1.394604000	-3.977614000
6	-0.000099000	1.394604000	-3.977614000
6	0.010793000	2.876810000	-1.652554000
6	0.000708000	2.876472000	1.667698000
6	0.000000000	0.000000000	3.328527000

1	0.944457573	-0.129242849	1.635848503
1	-1.888915145	-0.129242849	0.000000000
1	0.944457573	-0.129242849	-1.635848503
1	-3.471890882	-0.041706443	-3.416030456
1	-1.222423713	-0.041706443	-4.714760930
1	4.694314596	-0.041706443	-1.298730475
1	4.694314596	-0.041706443	1.298730475
1	-1.222423713	-0.041706443	4.714760930
1	-3.471890882	-0.041706443	3.416030456
1	1.222423713	0.041706443	4.714760930
1	3.471890882	0.041706443	3.416030456
1	-4.694314596	0.041706443	-1.298730475
1	-4.694314596	0.041706443	1.298730475
1	3.471890882	0.041706443	-3.416030456
1	1.222423713	0.041706443	-4.714760930

D_{3d}, KMLYP/6-311+G(*d*,*p*)

1	0.985526000	-0.774209000	-1.859377000
1	2.225855000	0.261120000	-0.019330000
1	0.952564000	-0.770616000	1.878479000
1	-0.952564000	0.770616000	1.878479000
1	-2.225855000	-0.261120000	-0.019330000
1	-0.985526000	0.774209000	-1.859377000
6	0.817680000	-0.073015000	-4.063009000
6	3.227212000	-0.563019000	-2.752964000
6	3.978783000	-0.586553000	-1.460668000
6	3.869492000	-0.674540000	1.321839000
6	3.078573000	-0.433364000	2.740326000
6	0.729125000	-0.164861000	4.213481000
6	-0.729125000	0.164861000	4.213481000
6	-3.078573000	0.433364000	2.740326000
6	-3.869492000	0.674540000	1.321839000
6	-3.978783000	0.586553000	-1.460668000
6	-3.227212000	0.563019000	-2.752964000
6	-0.817680000	0.073015000	-4.063009000
6	1.606067000	-0.463148000	-2.863436000
6	3.299380000	-0.319683000	-0.005462000
6	1.596892000	-0.462334000	2.868353000
6	-1.596892000	0.462334000	2.868353000
6	-3.299380000	0.319683000	-0.005462000
6	-1.606067000	0.463148000	-2.863436000
1	3.853626000	-0.662840000	-3.800973000
1	5.188293000	-0.779265000	-1.485709000
1	4.992197000	-1.165098000	1.393998000
1	3.774257000	-0.231330000	3.731055000
1	1.312329000	-0.258362000	5.286706000
1	-1.312329000	0.258362000	5.286706000
1	-3.774257000	0.231330000	3.731055000
1	-4.992197000	1.165098000	1.393998000
1	-5.188293000	0.779265000	-1.485709000
1	-3.853626000	0.662840000	-3.800973000
1	-1.390114000	-0.147552000	-5.126014000
1	1.390114000	0.147552000	-5.126014000

D_{3d}, BHLYP/6-311+G(*d*,*p*)

1	0.912806000	-0.318741000	-1.633431000
1	1.880427000	-0.184727000	-0.009860000
1	0.905250000	-0.267274000	1.638853000
1	-0.905250000	0.267274000	1.638853000
1	-1.880427000	0.184727000	-0.009860000
1	-0.912806000	0.318741000	-1.633431000
6	0.715384000	-0.099618000	-3.677523000
6	2.862919000	-0.459287000	-2.459572000
6	3.535934000	-0.558814000	-1.281806000
6	3.499329000	-0.572341000	1.213223000
6	2.789860000	-0.438698000	2.464759000
6	0.670903000	-0.111827000	3.741650000
6	-0.670903000	0.111827000	3.741650000
6	-2.789860000	0.438698000	2.464759000
6	-3.499329000	0.572341000	1.213223000
6	-3.535934000	0.558814000	-1.281806000
6	-2.862919000	0.459287000	-2.459572000
6	-0.715384000	0.099618000	-3.677523000
6	1.451244000	-0.284734000	-2.562076000
6	2.924848000	-0.434106000	0.000462000
6	1.454781000	-0.271163000	2.561424000
6	-1.454781000	0.271163000	2.561424000
6	-2.924848000	0.434106000	0.000462000
6	-1.451244000	0.284734000	-2.562076000
1	3.426120000	-0.523055000	-3.377566000
1	4.600891000	-0.728361000	-1.313789000

6	-0.000708000	-2.876472000	1.667698000
6	-0.010793000	-2.876810000	-1.652554000
6	0.000000000	0.000000000	-3.313273000
1	-0.024421000	5.240996000	-1.334034000
1	-0.005124000	5.241458000	1.348446000
1	0.008158000	3.782035000	3.876051000
1	0.004249000	1.459844000	5.216859000
1	-0.004249000	-1.459844000	5.216859000
1	-0.008158000	-3.782035000	3.876051000
1	0.005124000	-5.241458000	1.348446000
1	0.024421000	-5.240996000	-1.334034000
1	0.012715000	-3.781879000	-3.859995000
1	0.007462000	-1.458673000	-5.201085000
1	-0.007462000	1.458673000	-5.201085000
1	-0.012715000	3.781879000	-3.859995000

C_2, BHLYP/6-311+G(*d*,*p*)

1	-0.995822000	0.117056000	-1.664058000
1	-1.923717000	-0.110738000	0.000057000
1	-0.870927000	-0.411405000	1.643096000
1	0.870927000	0.411405000	1.643096000
1	1.923717000	0.110738000	0.000057000
1	0.995822000	-0.117056000	-1.664058000
6	-0.696741000	-0.193643000	-3.682047000
6	-2.808219000	-0.682355000	-2.454644000
6	-3.469890000	-0.826312000	-1.278729000
6	-3.446648000	-0.804131000	1.210954000
6	-2.756025000	-0.595682000	2.464814000
6	-0.661303000	-0.151694000	3.743914000
6	0.661303000	0.151694000	3.743914000
6	2.756025000	0.595682000	2.464814000
6	3.446648000	0.804131000	1.210954000
6	3.469890000	0.826312000	-1.278729000
6	2.808219000	0.682355000	-2.454644000
6	0.696741000	0.193643000	-3.682047000
6	-1.458146000	-0.227385000	-2.571234000
6	-2.904131000	-0.550658000	0.004170000
6	-1.429568000	-0.380984000	2.560546000
6	1.429568000	0.380984000	2.560546000
6	2.904131000	0.550658000	0.004170000
6	1.458146000	0.227385000	-2.571234000
1	-3.317171000	-0.962261000	-3.363855000
1	-4.476862000	-1.212843000	-1.303797000
1	-4.451367000	-1.195437000	1.264262000
1	-3.349814000	-0.636628000	3.365408000
1	-1.166605000	-0.245812000	4.692468000
1	1.166605000	0.245812000	4.692468000
1	3.349814000	0.636628000	3.365408000
1	4.451367000	1.195437000	1.264262000
1	4.476862000	1.212843000	-1.303797000
1	3.317171000	0.962261000	-3.363855000
1	1.134662000	0.462812000	-4.631496000
1	-1.134662000	-0.462812000	-4.631496000

C_2, OLYP/6-311+G(*d*,*p*)

1	-0.951957000	-0.149071000	-1.666518000
1	-1.878456000	-0.408152000	0.000668000
1	-0.914555000	-0.310855000	1.666661000
1	0.914555000	0.310855000	1.666661000
1	1.878456000	0.408152000	0.000668000
1	0.951957000	0.149071000	-1.666518000
6	-0.687572000	-0.169205000	-3.756515000
6	-2.814675000	-0.685602000	-2.491230000
6	-3.505159000	-0.844980000	-1.264126000
6	-3.510881000	-0.824396000	1.264788000
6	-2.823003000	-0.654343000	2.490906000
6	-0.690606000	-0.157505000	3.755458000
6	0.690606000	0.157505000	3.755458000
6	2.823003000	0.654343000	2.490906000
6	3.510881000	0.824396000	1.264788000
6	3.505159000	0.844980000	-1.264126000
6	2.814675000	0.685602000	-2.491230000
6	0.687572000	0.169205000	-3.756515000
6	-1.466663000	-0.329281000	-2.604872000
6	-2.928564000	-0.682892000	-0.000011000
6	-1.457586000	-0.368909000	2.604806000
6	1.457586000	0.368909000	2.604806000
6	2.928564000	0.682892000	-0.000011000
6	1.466663000	0.329281000	-2.604872000
1	-3.382789000	-0.861484000	-3.406369000
1	-4.559064000	-1.123097000	-1.320559000

1	4.554277000	-0.792648000	1.271780000
1	3.380588000	-0.479340000	3.367112000
1	1.177492000	-0.182918000	4.691489000
1	-1.177492000	0.182918000	4.691489000
1	-3.380588000	0.479340000	3.367112000
1	-4.554277000	0.792648000	1.271780000
1	-4.600891000	0.728361000	-1.313789000
1	-3.426120000	0.523055000	-3.377566000
1	-1.208136000	0.101775000	-4.637836000
1	1.208136000	-0.101775000	-4.637836000

D_{3d}, OLYP/6-311+G(*d*,*p*)

6	1.209185000	2.755699000	-0.060316000
6	0.302745000	3.802854000	-0.258562000
6	-1.096259000	3.638724000	-0.408906000
6	-1.759398000	2.406767000	-0.393920000
6	-3.124874000	2.133614000	-0.529458000
6	-3.694337000	0.837001000	-0.497306000
6	-2.969138000	-0.346944000	-0.324291000
6	-3.428071000	-1.667728000	-0.272856000
6	2.599730000	2.801814000	0.087422000
1	0.769749000	1.764232000	-0.014297000
1	0.690879000	4.821936000	-0.303143000
1	-1.123024000	1.537710000	-0.259255000
1	-4.777201000	0.767374000	-0.614668000
1	-1.896647000	-0.223163000	-0.211470000
1	3.091214000	3.775826000	0.056591000
1	-1.690098000	4.543748000	-0.548640000
1	-3.808501000	2.972773000	-0.669572000
1	-4.499457000	-1.847202000	-0.378082000
1	-3.091214000	-3.775826000	-0.056591000
6	-2.599730000	-2.801814000	-0.087422000
6	-1.209185000	-2.755699000	0.060316000
6	-0.302745000	-3.802854000	0.258562000
6	1.096259000	-3.638724000	0.408906000
6	1.759398000	-2.406767000	0.393920000
6	3.124874000	-2.133614000	0.529458000
6	3.694337000	-0.837001000	0.497306000
6	2.969138000	0.346944000	0.324291000
6	3.428071000	1.667728000	0.272856000
1	4.499457000	1.847202000	0.378082000
1	1.896647000	0.223163000	0.211470000
1	4.777201000	-0.767374000	0.614668000
1	3.808501000	-2.972773000	0.669572000
1	1.123024000	-1.537710000	0.259255000
1	1.690098000	-4.543748000	0.548640000
1	-0.690879000	-4.821936000	0.303143000
1	-0.769749000	-1.764232000	0.014297000

D_{6h}, B3LYP/6-311+G(*d*,*p*)

1	0.000000000	1.899665000	0.000000000
1	1.645158000	0.949833000	0.000000000
1	1.645158000	-0.949833000	0.000000000
1	0.000000000	-1.899665000	0.000000000
1	-1.645158000	-0.949833000	0.000000000
1	-1.645158000	0.949833000	0.000000000
6	-1.255563000	3.584887000	0.000000000
6	1.255563000	3.584887000	0.000000000
6	2.476821000	2.879793000	0.000000000
6	3.732385000	0.705094000	0.000000000
6	3.732385000	-0.705094000	0.000000000
6	2.476821000	-2.879793000	0.000000000
6	1.255563000	-3.584887000	0.000000000
6	-1.255563000	-3.584887000	0.000000000
6	-2.476821000	-2.879793000	0.000000000
6	-3.732385000	-0.705094000	0.000000000
6	-3.732385000	0.705094000	0.000000000
6	-2.476821000	2.879793000	0.000000000
6	0.000000000	2.982298000	0.000000000
6	2.582746000	1.491149000	0.000000000
6	2.582746000	-1.491149000	0.000000000
6	0.000000000	-2.982298000	0.000000000
6	-2.582746000	-1.491149000	0.000000000
6	-2.582746000	1.491149000	0.000000000
1	1.305074000	4.670892000	0.000000000
1	3.392574000	3.465673000	0.000000000
1	4.697648000	1.205219000	0.000000000
1	4.697648000	-1.205219000	0.000000000
1	3.392574000	-3.465673000	0.000000000
1	1.305074000	-4.670892000	0.000000000
1	-1.305074000	-4.670892000	0.000000000

1	-4.571346000	-1.076248000	1.320586000
1	-3.407686000	-0.755366000	3.406938000
1	-1.180194000	-0.244456000	4.726966000
1	1.180194000	0.244456000	4.726966000
1	3.407686000	0.755366000	3.406938000
1	4.571346000	1.076248000	1.320586000
1	4.559064000	1.123097000	-1.320559000
1	3.382789000	0.861484000	-3.406369000
1	1.165634000	0.308687000	-4.727748000
1	-1.165634000	-0.308687000	-4.727748000

D_{3h}, OLYP/6-311+G(*d*,*p*)

1	0.960772000	1.665183000	0.000000000
1	-0.960772000	1.665183000	0.000000000
1	-1.922477000	-0.000539000	0.000000000
1	-0.961705000	-1.664645000	0.000000000
1	0.961705000	-1.664645000	0.000000000
1	1.922477000	-0.000539000	0.000000000
6	2.898080000	2.491311000	0.000000000
6	0.708013000	3.756068000	0.000000000
6	-0.708013000	3.756068000	0.000000000
6	-2.898080000	2.491311000	0.000000000
6	-3.606578000	1.264155000	0.000000000
6	-3.606856000	-1.264877000	0.000000000
6	-2.898844000	-2.491191000	0.000000000
6	-0.708498000	-3.755466000	0.000000000
6	0.708498000	-3.755466000	0.000000000
6	2.898844000	-2.491191000	0.000000000
6	3.606856000	-1.264877000	0.000000000
6	3.606578000	1.264155000	0.000000000
6	1.504063000	2.604857000	0.000000000
6	-1.504063000	2.604857000	0.000000000
6	-3.007904000	0.000129000	0.000000000
6	-1.503841000	-2.604986000	0.000000000
6	1.503841000	-2.604986000	0.000000000
6	3.007904000	0.000129000	0.000000000
1	1.205304000	4.727658000	0.000000000
1	-1.205304000	4.727658000	0.000000000
1	-3.491553000	3.407265000	0.000000000
1	-4.696555000	1.320141000	0.000000000
1	-4.696924000	-1.320005000	0.000000000
1	-3.491620000	-3.407653000	0.000000000
1	-1.205002000	-4.727406000	0.000000000
1	1.205002000	-4.727406000	0.000000000
1	3.491620000	-3.407653000	0.000000000
1	4.696924000	-1.320005000	0.000000000
1	4.696555000	1.320141000	0.000000000
1	3.491553000	3.407265000	0.000000000

D_{6h}, OLYP/6-311+G(*d*,*p*)

1	0.000000000	0.000000000	1.922549000
1	1.664976000	0.000000000	0.961274000
1	1.664976000	0.000000000	-0.961274000
1	0.000000000	0.000000000	-1.922549000
1	-1.664976000	0.000000000	-0.961274000
1	-1.664976000	0.000000000	0.961274000
6	-1.264468000	0.000000000	3.606770000
6	1.264468000	0.000000000	3.606770000
6	2.491320000	0.000000000	2.898447000
6	3.755789000	0.000000000	0.708323000
6	3.755789000	0.000000000	-0.708323000
6	2.491320000	0.000000000	-2.898447000
6	1.264468000	0.000000000	-3.606770000
6	-1.264468000	0.000000000	-3.606770000
6	-2.491320000	0.000000000	-2.898447000
6	-3.755789000	0.000000000	-0.708323000
6	-3.755789000	0.000000000	0.708323000
6	-2.491320000	0.000000000	2.898447000
6	0.000000000	0.000000000	3.008036000
6	2.605036000	0.000000000	1.504018000
6	2.605036000	0.000000000	-1.504018000
6	0.000000000	0.000000000	-3.008036000
6	-2.605036000	0.000000000	-1.504018000
6	-2.605036000	0.000000000	1.504018000
1	1.320612000	0.000000000	4.696676000
1	3.407135000	0.000000000	3.492022000
1	4.727747000	0.000000000	1.204654000
1	4.727747000	0.000000000	-1.204654000
1	3.407135000	0.000000000	-3.492022000
1	1.320612000	0.000000000	-4.696676000
1	-1.320612000	0.000000000	-4.696676000

1	-3.392574000	-3.465673000	0.000000000	1	-3.407135000	0.000000000	-3.492022000
1	-4.697648000	-1.205219000	0.000000000	1	-4.727747000	0.000000000	-1.204654000
1	-4.697648000	1.205219000	0.000000000	1	-4.727747000	0.000000000	1.204654000
1	-3.392574000	3.465673000	0.000000000	1	-3.407135000	0.000000000	3.492022000
1	-1.305074000	4.670892000	0.000000000	1	-1.320612000	0.000000000	4.696676000

Table S18. Structures (Ångstroms) from stability analyses of **2-6**.

2, HF/6-311++G(*d,p*)

6	0.000000000	1.386421000	0.000000000
6	1.200676000	0.693210000	0.000000000
6	1.200676000	-0.693210000	0.000000000
6	0.000000000	-1.386421000	0.000000000
6	-1.200676000	-0.693210000	0.000000000
6	-1.200676000	0.693210000	0.000000000
1	2.131916000	1.230862000	0.000000000
1	0.000000000	2.461724000	0.000000000
1	2.131916000	-1.230862000	0.000000000
1	0.000000000	-2.461724000	0.000000000
1	-2.131916000	-1.230862000	0.000000000
1	-2.131916000	1.230862000	0.000000000

2, MP4/cc-pVDZ

6	0.000000000	-1.409679592	0.000000000
6	1.220818338	-0.704839796	0.000000000
6	1.220818338	0.704839796	0.000000000
6	0.000000000	1.409679592	0.000000000
6	-1.220818338	0.704839796	0.000000000
6	-1.220818338	-0.704839796	0.000000000
1	2.170764071	-1.253291220	0.000000000
1	0.000000000	-2.506582440	0.000000000
1	2.170764071	1.253291220	0.000000000
1	0.000000000	2.506582440	0.000000000
1	-2.170764071	1.253291220	0.000000000
1	-2.170764071	-1.253291220	0.000000000

2, CCSD(T)/cc-pVDZ

6	0.000000000	-1.409544747	0.000000000
6	1.220701559	-0.704772373	0.000000000
6	1.220701559	0.704772373	0.000000000
6	0.000000000	1.409544747	0.000000000
6	-1.220701559	0.704772373	0.000000000
6	-1.220701559	-0.704772373	0.000000000
1	2.170578356	-1.253183998	0.000000000
1	0.000000000	-2.506367997	0.000000000
1	2.170578356	1.253183998	0.000000000
1	0.000000000	2.506367997	0.000000000
1	-2.170578356	1.253183998	0.000000000
1	-2.170578356	-1.253183998	0.000000000

2, BHLYP/6-311+G(*d,p*)

6	0.000000000	0.000000000	1.385218000
6	1.199634000	0.000000000	0.692609000
6	1.199634000	0.000000000	-0.692609000
6	0.000000000	0.000000000	-1.385218000
6	-1.199634000	0.000000000	-0.692609000
6	-1.199634000	0.000000000	0.692609000
1	2.131887000	0.000000000	1.230846000
1	0.000000000	0.000000000	2.461692000
1	2.131887000	0.000000000	-1.230846000
1	0.000000000	0.000000000	-2.461692000
1	-2.131887000	0.000000000	-1.230846000
1	-2.131887000	0.000000000	1.230846000

3, HF/6-311++G(*d,p*)

1	0.000000000	1.850786000	0.000000000
1	1.602828000	0.925393000	0.000000000
1	1.602828000	-0.925393000	0.000000000
1	0.000000000	-1.850786000	0.000000000
1	-1.602828000	-0.925393000	0.000000000
1	-1.602828000	0.925393000	0.000000000
6	-1.216192000	3.572998000	0.000000000
6	1.216192000	3.572998000	0.000000000
6	2.486211000	2.839752000	0.000000000
6	3.702403000	0.733246000	0.000000000
6	3.702403000	-0.733246000	0.000000000
6	2.486211000	-2.839752000	0.000000000
6	-1.216192000	-3.572998000	0.000000000
6	-3.702403000	-0.733246000	0.000000000
6	-2.486211000	2.839752000	0.000000000
6	0.000000000	2.914977000	0.000000000
6	2.524444000	1.457488000	0.000000000
6	2.524444000	-1.457488000	0.000000000
6	0.000000000	-2.914977000	0.000000000
6	-2.524444000	-1.457488000	0.000000000
6	1.218740000	4.981342000	0.000000000
6	-1.218740000	4.981342000	0.000000000

2, MP2/cc-pVDZ

6	0.000000000	0.000000000	1.405745000
6	1.217411000	0.000000000	0.702873000
6	1.217411000	0.000000000	-0.702873000
6	0.000000000	0.000000000	-1.405745000
6	-1.217411000	0.000000000	-0.702873000
6	-1.217411000	0.000000000	0.702873000
1	2.165928000	0.000000000	1.250499000
1	0.000000000	0.000000000	2.500998000
1	2.165928000	0.000000000	-1.250499000
1	0.000000000	0.000000000	-2.500998000
1	-2.165928000	0.000000000	-1.250499000
1	-2.165928000	0.000000000	1.250499000

2, CCSD/DZ

6	0.000000000	-1.425359389	0.000000000
6	1.234397439	-0.712679694	0.000000000
6	1.234397439	0.712679694	0.000000000
6	0.000000000	1.425359389	0.000000000
6	-1.234397439	0.712679694	0.000000000
6	-1.234397439	-0.712679694	0.000000000
1	2.184224398	-1.261062543	0.000000000
1	0.000000000	-2.522125091	0.000000000
1	2.184224398	1.261062543	0.000000000
1	0.000000000	2.522125091	0.000000000
1	-2.184224398	1.261062543	0.000000000
1	-2.184224398	-1.261062543	0.000000000

2, KMLYP/6-311+G(*d,p*)

6	0.000000000	0.000000000	1.546957000
6	1.339704000	0.000000000	0.773479000
6	1.339704000	0.000000000	-0.773479000
6	0.000000000	0.000000000	-1.546957000
6	-1.339704000	0.000000000	-0.773479000
6	-1.339704000	0.000000000	0.773479000
1	2.397434000	0.000000000	1.384159000
1	0.000000000	0.000000000	2.768318000
1	2.397434000	0.000000000	-1.384159000
1	0.000000000	0.000000000	-2.768318000
1	-2.397434000	0.000000000	-1.384159000
1	-2.397434000	0.000000000	1.384159000

2, B3LYP/6-311+G(*d,p*)

6	0.000000000	1.394680000	0.000000000
6	1.207828000	0.697340000	0.000000000
6	1.207828000	-0.697340000	0.000000000
6	0.000000000	-1.394680000	0.000000000
6	-1.207828000	-0.697340000	0.000000000
6	-1.207828000	0.697340000	0.000000000
1	2.146914000	1.239521000	0.000000000
1	0.000000000	2.479042000	0.000000000
1	2.146914000	-1.239521000	0.000000000
1	0.000000000	-2.479042000	0.000000000
1	-2.146914000	-1.239521000	0.000000000
1	-2.146914000	1.239521000	0.000000000

3, MP2/cc-pVDZ

1	0.000000000	1.837759000	0.000000000
1	1.591546000	0.918879000	0.000000000
1	1.591546000	-0.918879000	0.000000000
1	0.000000000	-1.837759000	0.000000000
1	-1.591546000	-0.918879000	0.000000000
1	-1.591546000	0.918879000	0.000000000
6	-1.233553000	3.596979000	0.000000000
6	1.233553000	3.596979000	0.000000000
6	2.498298000	2.866778000	0.000000000
6	3.731852000	0.730201000	0.000000000
6	3.731852000	-0.730201000	0.000000000
6	2.498298000	-2.866778000	0.000000000
6	-1.233553000	-3.596979000	0.000000000
6	-3.731852000	-0.730201000	0.000000000
6	-2.498298000	2.866778000	0.000000000
6	0.000000000	2.926106000	0.000000000
6	2.534082000	1.463053000	0.000000000
6	2.534082000	-1.463053000	0.000000000
6	0.000000000	-2.926106000	0.000000000
6	-2.534082000	-1.463053000	0.000000000
6	1.236953000	5.035079000	0.000000000
6	-1.236953000	5.035079000	0.000000000

6	2.499038000	5.668054000	0.000000000
6	-2.499038000	5.668054000	0.000000000
6	0.000000000	5.657040000	0.000000000
1	0.000000000	6.733907000	0.000000000
6	-2.524444000	1.457488000	0.000000000
6	-3.702403000	0.733246000	0.000000000
6	-3.704599000	3.546131000	0.000000000
6	-4.923339000	1.435211000	0.000000000
6	-3.659160000	4.998257000	0.000000000
6	-6.158198000	0.669797000	0.000000000
6	-4.899140000	2.828520000	0.000000000
1	-5.831735000	3.366954000	0.000000000
6	-2.486211000	-2.839752000	0.000000000
6	-4.923339000	-1.435211000	0.000000000
6	-3.704599000	-3.546131000	0.000000000
6	-6.158198000	-0.669797000	0.000000000
6	-3.659160000	-4.998257000	0.000000000
6	-4.899140000	-2.828520000	0.000000000
1	-5.831735000	-3.366954000	0.000000000
6	1.216192000	-3.572998000	0.000000000
6	1.218740000	-4.981342000	0.000000000
6	-1.218740000	-4.981342000	0.000000000
6	2.499038000	-5.668054000	0.000000000
6	-2.499038000	-5.668054000	0.000000000
6	0.000000000	-5.657040000	0.000000000
1	0.000000000	-6.733907000	0.000000000
6	4.923339000	-1.435211000	0.000000000
6	3.704599000	-3.546131000	0.000000000
6	6.158198000	-0.669797000	0.000000000
6	3.659160000	-4.998257000	0.000000000
6	4.899140000	-2.828520000	0.000000000
1	5.831735000	-3.366954000	0.000000000
6	3.704599000	3.546131000	0.000000000
6	4.923339000	1.435211000	0.000000000
6	3.659160000	4.998257000	0.000000000
6	6.158198000	0.669797000	0.000000000
6	4.899140000	2.828520000	0.000000000
1	5.831735000	3.366954000	0.000000000
1	-2.499096000	-6.743994000	0.000000000
1	-4.590922000	-5.536278000	0.000000000
1	-7.090018000	-1.207716000	0.000000000
1	-7.090018000	1.207716000	0.000000000
1	-4.590922000	5.536278000	0.000000000
1	-2.499096000	6.743994000	0.000000000
1	2.499096000	6.743994000	0.000000000
1	4.590922000	5.536278000	0.000000000
1	7.090018000	1.207716000	0.000000000
1	7.090018000	-1.207716000	0.000000000
1	4.590922000	-5.536278000	0.000000000
1	2.499096000	-6.743994000	0.000000000

3, BHLYP/6-311+G(*d*,*p*)

1	0.000000000	1.835038000	0.000000000
1	1.589190000	0.917519000	0.000000000
1	1.589190000	-0.917519000	0.000000000
1	0.000000000	-1.835038000	0.000000000
1	-1.589190000	-0.917519000	0.000000000
1	-1.589190000	0.917519000	0.000000000
6	-1.215814000	3.560365000	0.000000000
6	1.215814000	3.560365000	0.000000000
6	2.475459000	2.833109000	0.000000000
6	3.691274000	0.727256000	0.000000000
6	3.691274000	-0.727256000	0.000000000
6	2.475459000	-2.833109000	0.000000000
6	-1.215814000	-3.560365000	0.000000000
6	-3.691274000	-0.727256000	0.000000000
6	-2.475459000	2.833109000	0.000000000
6	0.000000000	2.903235000	0.000000000
6	2.514275000	1.451618000	0.000000000
6	2.514275000	-1.451618000	0.000000000
6	0.000000000	-2.903235000	0.000000000
6	-2.514275000	-1.451618000	0.000000000
6	1.218753000	4.973521000	0.000000000
6	-1.218753000	4.973521000	0.000000000
6	2.487073000	5.653979000	0.000000000
6	-2.487073000	5.653979000	0.000000000
6	0.000000000	5.647579000	0.000000000
1	0.000000000	6.725701000	0.000000000
6	-2.514275000	1.451618000	0.000000000
6	-3.691274000	0.727256000	0.000000000
6	-3.697818000	3.542232000	0.000000000

6	2.508533000	5.722783000	0.000000000
6	-2.508533000	5.722783000	0.000000000
6	0.000000000	5.719554000	0.000000000
1	0.000000000	6.817655000	0.000000000
6	-2.534082000	1.463053000	0.000000000
6	-3.731852000	0.730201000	0.000000000
6	-3.742030000	3.588772000	0.000000000
6	-4.978982000	1.446307000	0.000000000
6	-3.701809000	5.033844000	0.000000000
6	-6.210342000	0.688938000	0.000000000
6	-4.953279000	2.859777000	0.000000000
1	-5.904262000	3.408827000	0.000000000
6	-2.498298000	-2.866778000	0.000000000
6	-4.978982000	-1.446307000	0.000000000
6	-3.742030000	-3.588772000	0.000000000
6	-6.210342000	-0.688938000	0.000000000
6	-3.701809000	-5.033844000	0.000000000
6	-4.953279000	-2.859777000	0.000000000
1	-5.904262000	-3.408827000	0.000000000
6	1.233553000	-3.596979000	0.000000000
6	1.236953000	-5.035079000	0.000000000
6	-1.236953000	-5.035079000	0.000000000
6	2.508533000	-5.722783000	0.000000000
6	-2.508533000	-5.722783000	0.000000000
6	0.000000000	-5.719554000	0.000000000
1	0.000000000	-6.817655000	0.000000000
6	4.978982000	-1.446307000	0.000000000
6	3.742030000	-3.588772000	0.000000000
6	6.210342000	-0.688938000	0.000000000
6	3.701809000	-5.033844000	0.000000000
6	4.953279000	-2.859777000	0.000000000
1	5.904262000	-3.408827000	0.000000000
6	3.742030000	3.588772000	0.000000000
6	4.978982000	1.446307000	0.000000000
6	3.701809000	5.033844000	0.000000000
6	6.210342000	0.688938000	0.000000000
6	4.953279000	2.859777000	0.000000000
1	5.904262000	3.408827000	0.000000000
1	-2.510467000	-6.819604000	0.000000000
1	-4.650717000	-5.583930000	0.000000000
1	-7.161184000	-1.235674000	0.000000000
1	-7.161184000	1.235674000	0.000000000
1	-4.650717000	5.583930000	0.000000000
1	-2.510467000	6.819604000	0.000000000
1	2.510467000	6.819604000	0.000000000
1	4.650717000	5.583930000	0.000000000
1	7.161184000	1.235674000	0.000000000
1	7.161184000	-1.235674000	0.000000000
1	4.650717000	-5.583930000	0.000000000
1	2.510467000	-6.819604000	0.000000000

3, KMLYP/6-311+G(*d*,*p*)

1	0.000000000	0.000000000	2.055248000
1	0.000000000	1.779901000	1.027630000
1	0.000000000	1.779901000	-1.027630000
1	0.000000000	0.000000000	-2.055248000
1	0.000000000	-1.779901000	-1.027630000
1	0.000000000	-1.779901000	1.027630000
6	0.000000000	-1.357089000	3.989597000
6	0.000000000	1.357089000	3.989597000
6	0.000000000	2.776551000	3.170069000
6	0.000000000	4.133638000	0.819526000
6	0.000000000	4.133638000	-0.819526000
6	0.000000000	2.776551000	-3.170069000
6	0.000000000	-1.357089000	-3.989597000
6	0.000000000	-4.133638000	-0.819526000
6	0.000000000	-2.776551000	3.170069000
6	0.000000000	0.000000000	3.259805000
6	0.000000000	2.823077000	1.629904000
6	0.000000000	2.823077000	-1.629904000
6	0.000000000	0.000000000	-3.259805000
6	0.000000000	-2.823077000	-1.629904000
6	0.000000000	1.361438000	5.565231000
6	0.000000000	-1.361438000	5.565231000
6	0.000000000	2.788868000	6.326947000
6	0.000000000	-2.788868000	6.326947000
6	0.000000000	0.000000000	6.317894000
1	0.000000000	0.000000000	7.541787000
6	0.000000000	-2.823077000	1.629904000
6	0.000000000	-4.133638000	0.819526000
6	0.000000000	-4.138919000	3.961651000

6	-4.916572000	1.431289000	0.000000000
6	-3.652953000	4.980858000	0.000000000
6	-6.140026000	0.673121000	0.000000000
6	-4.890947000	2.823789000	0.000000000
1	-5.824628000	3.362851000	0.000000000
6	-2.475459000	-2.833109000	0.000000000
6	-4.916572000	-1.431289000	0.000000000
6	-3.697818000	-3.542232000	0.000000000
6	-6.140026000	-0.673121000	0.000000000
6	-3.652953000	-4.980858000	0.000000000
6	-4.890947000	-2.823789000	0.000000000
1	-5.824628000	-3.362851000	0.000000000
6	1.215814000	-3.560365000	0.000000000
6	1.218753000	-4.973521000	0.000000000
6	-1.218753000	-4.973521000	0.000000000
6	2.487073000	-5.653979000	0.000000000
6	-2.487073000	-5.653979000	0.000000000
6	0.000000000	-5.647579000	0.000000000
1	0.000000000	-6.725701000	0.000000000
6	4.916572000	-1.431289000	0.000000000
6	3.697818000	-3.542232000	0.000000000
6	6.140026000	-0.673121000	0.000000000
6	3.652953000	-4.980858000	0.000000000
6	4.890947000	-2.823789000	0.000000000
1	5.824628000	-3.362851000	0.000000000
6	3.697818000	3.542232000	0.000000000
6	4.916572000	1.431289000	0.000000000
6	3.652953000	4.980858000	0.000000000
6	6.140026000	0.673121000	0.000000000
6	4.890947000	2.823789000	0.000000000
1	5.824628000	3.362851000	0.000000000
1	-2.487749000	-6.731180000	0.000000000
1	-4.585499000	-5.520044000	0.000000000
1	-7.073248000	-1.211136000	0.000000000
1	-7.073248000	1.211136000	0.000000000
1	-4.585499000	5.520044000	0.000000000
1	-2.487749000	6.731180000	0.000000000
1	2.487749000	6.731180000	0.000000000
1	4.585499000	5.520044000	0.000000000
1	7.073248000	1.211136000	0.000000000
1	7.073248000	-1.211136000	0.000000000
1	4.585499000	-5.520044000	0.000000000
1	2.487749000	-6.731180000	0.000000000

3, B3LYP/6-311+G(*d*,*p*)

1	0.000000000	1.846802000	0.000000000
1	1.599378000	0.923401000	0.000000000
1	1.599378000	-0.923401000	0.000000000
1	0.000000000	-1.846802000	0.000000000
1	-1.599378000	-0.923401000	0.000000000
1	-1.599378000	0.923401000	0.000000000
6	-1.225657000	3.583299000	0.000000000
6	1.225657000	3.583299000	0.000000000
6	2.490399000	2.853100000	0.000000000
6	3.716056000	0.730199000	0.000000000
6	3.716056000	-0.730199000	0.000000000
6	2.490399000	-2.853100000	0.000000000
6	-1.225657000	-3.583299000	0.000000000
6	-3.716056000	-0.730199000	0.000000000
6	-2.490399000	2.853100000	0.000000000
6	0.000000000	2.923570000	0.000000000
6	2.531886000	1.461785000	0.000000000
6	2.531886000	-1.461785000	0.000000000
6	0.000000000	-2.923570000	0.000000000
6	-2.531886000	-1.461785000	0.000000000
6	1.228786000	5.010310000	0.000000000
6	-1.228786000	5.010310000	0.000000000
6	2.500592000	5.691417000	0.000000000
6	-2.500592000	5.691417000	0.000000000
6	0.000000000	5.687885000	0.000000000
1	0.000000000	6.773917000	0.000000000
6	-2.531886000	1.461785000	0.000000000
6	-3.716056000	0.730199000	0.000000000
6	-3.724663000	3.569315000	0.000000000
6	-4.953449000	1.440995000	0.000000000
6	-3.678616000	5.011285000	0.000000000
6	-6.179208000	0.680132000	0.000000000
6	-4.925853000	2.843943000	0.000000000
1	-5.866384000	3.386958000	0.000000000
6	-2.490399000	-2.853100000	0.000000000
6	-4.953449000	-1.440995000	0.000000000

6	0.000000000	-5.500351000	1.603578000
6	0.000000000	-4.084872000	5.578698000
6	0.000000000	-6.873731000	0.748248000
6	0.000000000	-5.471471000	3.158951000
1	0.000000000	-6.531393000	3.770894000
6	0.000000000	-2.776551000	-3.170069000
6	0.000000000	-5.500351000	-1.603578000
6	0.000000000	-4.138919000	-3.961651000
6	0.000000000	-6.873731000	-0.748248000
6	0.000000000	-4.084872000	-5.578698000
6	0.000000000	-5.471471000	-3.158951000
1	0.000000000	-6.531393000	-3.770894000
6	0.000000000	1.357089000	-3.989597000
6	0.000000000	1.361438000	-5.565231000
6	0.000000000	-1.361438000	-5.565231000
6	0.000000000	2.788868000	-6.326947000
6	0.000000000	-2.788868000	-6.326947000
6	0.000000000	0.000000000	-6.317894000
1	0.000000000	0.000000000	-7.541787000
6	0.000000000	5.500351000	-1.603578000
6	0.000000000	4.138919000	-3.961651000
6	0.000000000	6.873731000	-0.748248000
6	0.000000000	4.084872000	-5.578698000
6	0.000000000	5.471471000	-3.158951000
1	0.000000000	6.531393000	-3.770894000
6	0.000000000	4.138919000	3.961651000
6	0.000000000	5.500351000	1.603578000
6	0.000000000	4.084872000	5.578698000
6	0.000000000	6.873731000	0.748248000
6	0.000000000	5.471471000	3.158951000
1	0.000000000	6.531393000	3.770894000
1	0.000000000	-2.790047000	-7.549367000
1	0.000000000	-5.142928000	-6.190930000
1	0.000000000	-7.932967000	-1.358440000
1	0.000000000	-7.932967000	1.358440000
1	0.000000000	-5.142928000	6.190930000
1	0.000000000	-2.790047000	7.549367000
1	0.000000000	2.790047000	7.549367000
1	0.000000000	5.142928000	6.190930000
1	0.000000000	7.932967000	1.358440000
1	0.000000000	7.932967000	-1.358440000
1	0.000000000	5.142928000	-6.190930000
1	0.000000000	2.790047000	-7.549367000

4, HF/6-311++G(*d*,*p*)

6	0.000000000	-1.425735000	0.000000000
6	1.234722000	-0.712867000	0.000000000
6	1.234722000	0.712867000	0.000000000
6	0.000000000	1.425735000	0.000000000
6	-1.234722000	0.712867000	0.000000000
6	-1.234722000	-0.712867000	0.000000000
6	-1.246104000	-3.511852000	0.000000000
6	1.246104000	-3.511852000	0.000000000
6	2.418301000	-2.835083000	0.000000000
6	3.664405000	-0.676768000	0.000000000
6	3.664405000	0.676768000	0.000000000
6	2.418301000	2.835083000	0.000000000
6	1.246104000	3.511852000	0.000000000
6	-1.246104000	3.511852000	0.000000000
6	-2.418301000	2.835083000	0.000000000
6	-3.664405000	0.676768000	0.000000000
6	-3.664405000	-0.676768000	0.000000000
6	-2.418301000	-2.835083000	0.000000000
6	0.000000000	-2.822197000	0.000000000
6	2.444095000	-1.411099000	0.000000000
6	2.444095000	1.411099000	0.000000000
6	0.000000000	2.822197000	0.000000000
6	-2.444095000	1.411099000	0.000000000
6	-2.444095000	-1.411099000	0.000000000
1	1.243186000	-4.587735000	0.000000000
1	3.351502000	-3.370498000	0.000000000
1	4.594688000	-1.217237000	0.000000000
1	4.594688000	1.217237000	0.000000000
1	3.351502000	3.370498000	0.000000000
1	1.243186000	4.587735000	0.000000000
1	-1.243186000	4.587735000	0.000000000
1	-3.351502000	3.370498000	0.000000000
1	-4.594688000	1.217237000	0.000000000
1	-4.594688000	-1.217237000	0.000000000
1	-3.351502000	-3.370498000	0.000000000
1	-1.243186000	-4.587735000	0.000000000

6	-3.724663000	-3.569315000	0.000000000
6	-6.179208000	-0.680132000	0.000000000
6	-3.678616000	-5.011285000	0.000000000
6	-4.925853000	-2.843943000	0.000000000
1	-5.866384000	-3.386958000	0.000000000
6	1.225657000	-3.583299000	0.000000000
6	1.228786000	-5.010310000	0.000000000
6	-1.228786000	-5.010310000	0.000000000
6	2.500592000	-5.691417000	0.000000000
6	-2.500592000	-5.691417000	0.000000000
6	0.000000000	-5.687885000	0.000000000
1	0.000000000	-6.773917000	0.000000000
6	4.953449000	-1.440995000	0.000000000
6	3.724663000	-3.569315000	0.000000000
6	6.179208000	-0.680132000	0.000000000
6	3.678616000	-5.011285000	0.000000000
6	4.925853000	-2.843943000	0.000000000
1	5.866384000	-3.386958000	0.000000000
6	3.724663000	3.569315000	0.000000000
6	4.953449000	1.440995000	0.000000000
6	3.678616000	5.011285000	0.000000000
6	6.179208000	0.680132000	0.000000000
6	4.925853000	2.843943000	0.000000000
1	5.866384000	3.386958000	0.000000000
1	-2.502289000	-6.776549000	0.000000000
1	-4.617519000	-5.555320000	0.000000000
1	-7.119808000	-1.221229000	0.000000000
1	-7.119808000	1.221229000	0.000000000
1	-4.617519000	5.555320000	0.000000000
1	-2.502289000	6.776549000	0.000000000
1	2.502289000	6.776549000	0.000000000
1	4.617519000	5.555320000	0.000000000
1	7.119808000	1.221229000	0.000000000
1	7.119808000	-1.221229000	0.000000000
1	4.617519000	-5.555320000	0.000000000
1	2.502289000	-6.776549000	0.000000000

4, MP2/cc-pVDZ

6	0.000000000	-1.431751000	0.000000000
6	1.239932000	-0.715875000	0.000000000
6	1.239932000	0.715875000	0.000000000
6	0.000000000	1.431751000	0.000000000
6	-1.239932000	0.715875000	0.000000000
6	-1.239932000	-0.715875000	0.000000000
6	-1.251509000	-3.554250000	0.000000000
6	1.251509000	-3.554250000	0.000000000
6	2.452316000	-2.860963000	0.000000000
6	3.703825000	-0.693286000	0.000000000
6	3.703825000	0.693286000	0.000000000
6	2.452316000	2.860963000	0.000000000
6	1.251509000	3.554250000	0.000000000
6	-1.251509000	3.554250000	0.000000000
6	-2.452316000	2.860963000	0.000000000
6	-3.703825000	0.693286000	0.000000000
6	-3.703825000	-0.693286000	0.000000000
6	-2.452316000	-2.860963000	0.000000000
6	0.000000000	-2.864543000	0.000000000
6	2.480767000	-1.432272000	0.000000000
6	2.480767000	1.432272000	0.000000000
6	0.000000000	2.864543000	0.000000000
6	-2.480767000	1.432272000	0.000000000
6	-2.480767000	-1.432272000	0.000000000
1	1.250154000	-4.650901000	0.000000000
1	3.402722000	-3.408116000	0.000000000
1	4.652876000	-1.242786000	0.000000000
1	4.652876000	1.242786000	0.000000000
1	3.402722000	3.408116000	0.000000000
1	1.250154000	4.650901000	0.000000000
1	-1.250154000	4.650901000	0.000000000
1	-3.402722000	3.408116000	0.000000000
1	-4.652876000	1.242786000	0.000000000
1	-4.652876000	-1.242786000	0.000000000
1	-3.402722000	-3.408116000	0.000000000
1	-1.250154000	-4.650901000	0.000000000

4, BHLYP/6-311+G(*d*,*p*)

6	0.000000000	1.418784000	0.000000000
6	1.228703000	0.709392000	0.000000000
6	1.228703000	-0.709392000	0.000000000
6	0.000000000	-1.418784000	0.000000000
6	-1.228703000	-0.709392000	0.000000000

4, KMLYP/6-311+G(*d*,*p*)

6	0.000000000	1.587428000	0.000000000
6	1.374753000	0.793714000	0.000000000
6	1.374753000	-0.793714000	0.000000000
6	0.000000000	-1.587428000	0.000000000
6	-1.374753000	-0.793714000	0.000000000
6	-1.374753000	0.793714000	0.000000000
6	-1.388781000	3.916929000	0.000000000
6	1.388781000	3.916929000	0.000000000
6	2.697770000	3.161184000	0.000000000
6	4.086551000	0.755745000	0.000000000
6	4.086551000	-0.755745000	0.000000000
6	2.697770000	-3.161184000	0.000000000
6	1.388781000	-3.916929000	0.000000000
6	-1.388781000	-3.916929000	0.000000000
6	-2.697770000	-3.161184000	0.000000000
6	-4.086551000	-0.755745000	0.000000000
6	-4.086551000	0.755745000	0.000000000
6	-2.697770000	3.161184000	0.000000000
6	0.000000000	3.152471000	0.000000000
6	2.730120000	1.576235000	0.000000000
6	2.730120000	-1.576235000	0.000000000
6	0.000000000	-3.152471000	0.000000000
6	-2.730120000	-1.576235000	0.000000000
6	-2.730120000	1.576235000	0.000000000
1	1.386763000	5.139521000	0.000000000
1	3.757574000	3.770732000	0.000000000
1	5.144337000	1.368788000	0.000000000
1	5.144337000	-1.368788000	0.000000000
1	3.757574000	-3.770732000	0.000000000
1	1.386763000	-5.139521000	0.000000000
1	-1.386763000	-5.139521000	0.000000000
1	-3.757574000	-3.770732000	0.000000000
1	-5.144337000	-1.368788000	0.000000000
1	-5.144337000	1.368788000	0.000000000
1	-3.757574000	3.770732000	0.000000000
1	-1.386763000	5.139521000	0.000000000

4, B3LYP/6-311+G(*d*,*p*)

6	0.000000000	-1.425833000	0.000000000
6	1.234808000	-0.712917000	0.000000000
6	1.234808000	0.712917000	0.000000000
6	0.000000000	1.425833000	0.000000000
6	-1.234808000	0.712917000	0.000000000

6	-1.228703000	0.709392000	0.000000000	6	-1.234808000	-0.712917000	0.000000000
6	-1.240445000	3.506742000	0.000000000	6	-1.246881000	-3.530185000	0.000000000
6	1.240445000	3.506742000	0.000000000	6	1.246881000	-3.530185000	0.000000000
6	2.416705000	2.827628000	0.000000000	6	2.433789000	-2.844923000	0.000000000
6	3.657150000	0.679114000	0.000000000	6	3.680670000	-0.685261000	0.000000000
6	3.657150000	-0.679114000	0.000000000	6	3.680670000	0.685261000	0.000000000
6	2.416705000	-2.827628000	0.000000000	6	2.433789000	2.844923000	0.000000000
6	1.240445000	-3.506742000	0.000000000	6	1.246881000	3.530185000	0.000000000
6	-1.240445000	-3.506742000	0.000000000	6	-1.246881000	3.530185000	0.000000000
6	-2.416705000	-2.827628000	0.000000000	6	-2.433789000	2.844923000	0.000000000
6	-3.657150000	-0.679114000	0.000000000	6	-3.680670000	0.685261000	0.000000000
6	-3.657150000	0.679114000	0.000000000	6	-3.680670000	-0.685261000	0.000000000
6	-2.416705000	2.827628000	0.000000000	6	-2.433789000	-2.844923000	0.000000000
6	0.000000000	2.823404000	0.000000000	6	0.000000000	-2.845742000	0.000000000
6	2.445139000	1.411702000	0.000000000	6	2.464485000	-1.422871000	0.000000000
6	2.445139000	-1.411702000	0.000000000	6	2.464485000	1.422871000	0.000000000
6	0.000000000	-2.823404000	0.000000000	6	0.000000000	2.845742000	0.000000000
6	-2.445139000	-1.411702000	0.000000000	6	-2.464485000	1.422871000	0.000000000
6	-2.445139000	1.411702000	0.000000000	6	-2.464485000	-1.422871000	0.000000000
1	1.237718000	4.583917000	0.000000000	1	1.245677000	-4.615084000	0.000000000
1	3.350930000	3.363854000	0.000000000	1	3.373942000	-3.386330000	0.000000000
1	4.588648000	1.220063000	0.000000000	1	4.619619000	-1.228754000	0.000000000
1	4.588648000	-1.220063000	0.000000000	1	4.619619000	1.228754000	0.000000000
1	3.350930000	-3.363854000	0.000000000	1	3.373942000	3.386330000	0.000000000
1	1.237718000	-4.583917000	0.000000000	1	1.245677000	4.615084000	0.000000000
1	-1.237718000	-4.583917000	0.000000000	1	-1.245677000	4.615084000	0.000000000
1	-3.350930000	-3.363854000	0.000000000	1	-3.373942000	3.386330000	0.000000000
1	-4.588648000	-1.220063000	0.000000000	1	-4.619619000	1.228754000	0.000000000
1	-4.588648000	1.220063000	0.000000000	1	-4.619619000	-1.228754000	0.000000000
1	-3.350930000	3.363854000	0.000000000	1	-3.373942000	-3.386330000	0.000000000
1	-1.237718000	4.583917000	0.000000000	1	-1.245677000	-4.615084000	0.000000000

5, HF/6-311++G(*d*,*p*)

6	0.718664000	-0.004492000	-2.427029000
6	-0.718664000	0.004492000	-2.427029000
6	1.404873000	-0.008781000	-3.697823000
1	2.480773000	-0.015505000	-3.697541000
6	-1.404873000	0.008781000	-3.697823000
6	0.721117000	-0.004507000	-4.852597000
1	-2.480773000	0.015505000	-3.697541000
1	1.242846000	-0.007768000	-5.792913000
6	-0.721117000	0.004507000	-4.852597000
1	-1.242846000	0.007768000	-5.792913000
6	1.394596000	-0.008716000	-1.232077000
1	2.471029000	-0.015444000	-1.231176000
6	-1.394596000	0.008716000	-1.232077000
1	-2.471029000	0.015444000	-1.231176000
6	-0.713202000	0.004458000	0.000000000
6	0.713202000	-0.004458000	0.000000000
6	1.394596000	-0.008716000	1.232077000
1	2.471029000	-0.015444000	1.231176000
6	-1.394596000	0.008716000	1.232077000
6	0.718664000	-0.004492000	2.427029000
1	-2.471029000	0.015444000	1.231176000
6	-0.718664000	0.004492000	2.427029000
6	-1.404873000	0.008781000	3.697823000
1	-2.480773000	0.015505000	3.697541000
6	-0.721117000	0.004507000	4.852597000
6	1.404873000	-0.008781000	3.697823000
1	-1.242846000	0.007768000	5.792913000
1	2.480773000	-0.015505000	3.697541000
6	0.721117000	-0.004507000	4.852597000
1	1.242846000	-0.007768000	5.792913000

5, MP2/cc-pVDZ

6	0.727395000	-0.004546000	-2.463923000
6	-0.727395000	0.004546000	-2.463923000
6	1.417025000	-0.008857000	-3.722984000
1	2.513605000	-0.015710000	-3.721992000
6	-1.417025000	0.008857000	-3.722984000
6	0.716425000	-0.004478000	-4.915281000
1	-2.513605000	0.015710000	-3.721992000
1	1.257790000	-0.007861000	-5.867447000
6	-0.716425000	0.004478000	-4.915281000
1	-1.257790000	0.007861000	-5.867447000
6	1.417428000	-0.008859000	-1.238621000
1	2.515443000	-0.015722000	-1.240083000
6	-1.417428000	0.008859000	-1.238621000
1	-2.515443000	0.015722000	-1.240083000
6	-0.730561000	0.004566000	0.000000000
6	0.730561000	-0.004566000	0.000000000
6	1.417428000	-0.008859000	1.238621000
1	2.515443000	-0.015722000	1.240083000
6	-1.417428000	0.008859000	1.238621000
6	0.727395000	-0.004546000	2.463923000
1	-2.515443000	0.015722000	1.240083000
6	-0.727395000	0.004546000	2.463923000
6	-1.417025000	0.008857000	3.722984000
1	-2.513605000	0.015710000	3.721992000
6	-0.716425000	0.004478000	4.915281000
6	1.417025000	-0.008857000	3.722984000
1	-1.257790000	0.007861000	5.867447000
1	2.513605000	-0.015710000	3.721992000
6	0.716425000	-0.004478000	4.915281000
1	1.257790000	-0.007861000	5.867447000

5, KMLYP/6-311+G(*d*,*p*)

6	0.000000000	2.707852000	0.805203000
6	0.000000000	2.707852000	-0.805203000
6	0.000000000	4.120843000	1.565829000
1	0.000000000	4.121725000	2.788901000
6	0.000000000	4.120843000	-1.565829000
6	0.000000000	5.411872000	0.801099000
1	0.000000000	4.121725000	-2.788901000
1	0.000000000	6.480183000	1.393297000
6	0.000000000	5.411872000	-0.801099000
1	0.000000000	6.480183000	-1.393297000
6	0.000000000	1.371771000	1.557956000
1	0.000000000	1.371471000	2.782173000
6	0.000000000	1.371771000	-1.557956000
1	0.000000000	1.371471000	-2.782173000
6	0.000000000	0.000000000	-0.802383000
6	0.000000000	0.000000000	0.802383000

5, BHLYP/6-311+G(*d*,*p*)

6	0.000000000	2.427823000	0.718672000
6	0.000000000	2.427823000	-0.718672000
6	0.000000000	3.684990000	1.399790000
1	0.000000000	3.683995000	2.476960000
6	0.000000000	3.684990000	-1.399790000
6	0.000000000	4.847821000	0.713433000
1	0.000000000	3.683995000	-2.476960000
1	0.000000000	5.787211000	1.238925000
6	0.000000000	4.847821000	-0.713433000
1	-0.000001000	5.787211000	-1.238925000
6	0.000000000	1.226913000	1.394805000
1	0.000000000	1.227026000	2.472664000
6	0.000000000	1.226913000	-1.394805000
1	0.000000000	1.227026000	-2.472664000
6	0.000000000	0.000000000	-0.717441000
6	0.000000000	0.000000000	0.717441000

6	0.000000000	-1.371771000	1.557956000
1	0.000000000	-1.371471000	2.782173000
6	0.000000000	-1.371771000	-1.557956000
6	0.000000000	-2.707852000	0.805203000
1	0.000000000	-1.371471000	-2.782173000
6	0.000000000	-2.707852000	-0.805203000
6	0.000000000	-4.120843000	-1.565829000
1	0.000000000	-4.121725000	-2.788901000
6	0.000000000	-5.411872000	-0.801099000
6	0.000000000	-4.120843000	1.565829000
1	0.000000000	-6.480183000	-1.393297000
1	0.000000000	-4.121725000	2.788901000
6	0.000000000	-5.411872000	0.801099000
1	0.000000000	-6.480183000	1.393297000

5, B3LYP/6-311+G(*d*,*p*)

6	0.725139000	-0.004532000	-2.447282000
6	-0.725139000	0.004532000	-2.447282000
6	1.407744000	-0.008799000	-3.708062000
1	2.492819000	-0.015581000	-3.707589000
6	-1.407744000	0.008799000	-3.708062000
6	0.715046000	-0.004469000	-4.883356000
1	-2.492819000	0.015581000	-3.707589000
1	1.245331000	-0.007784000	-5.829004000
6	-0.715046000	0.004469000	-4.883356000
1	-1.245331000	0.007784000	-5.829004000
6	1.404760000	-0.008780000	-1.234267000
1	2.490618000	-0.015567000	-1.234442000
6	-1.404760000	0.008780000	-1.234267000
1	-2.490618000	0.015567000	-1.234442000
6	-0.725114000	0.004532000	0.000000000
6	0.725114000	-0.004532000	0.000000000
6	1.404760000	-0.008780000	1.234267000
1	2.490618000	-0.015567000	1.234442000
6	-1.404760000	0.008780000	1.234267000
6	0.725139000	-0.004532000	2.447282000
1	-2.490618000	0.015567000	1.234442000
6	-0.725139000	0.004532000	2.447282000
6	-1.407744000	0.008799000	3.708062000
1	-2.492819000	0.015581000	3.707589000
6	-0.715046000	0.004469000	4.883356000
6	1.407744000	-0.008799000	3.708062000
1	-1.245331000	0.007784000	5.829004000
1	2.492819000	-0.015581000	3.707589000
6	0.715046000	-0.004469000	4.883356000
1	1.245331000	-0.007784000	5.829004000

6, MP2/cc-pVDZ

6	-2.226822000	-1.187631000	0.397429000
6	-2.226822000	1.187631000	0.397429000
6	-3.628644000	-1.622849000	0.370031000
1	-3.862624000	-2.684034000	0.213314000
6	-3.628644000	1.622849000	0.370031000
6	-4.673453000	-0.737620000	0.480374000
1	-3.862624000	2.684034000	0.213314000
1	-5.678109000	-1.178846000	0.452203000
6	-4.673453000	0.737620000	0.480374000
1	-5.678109000	1.178846000	0.452203000
6	-1.271976000	-1.566842000	-0.496948000
1	-1.466267000	-2.396323000	-1.193815000
6	-1.271976000	1.566842000	-0.496948000
1	-1.466267000	2.396323000	-1.193815000
6	0.000000000	0.792661000	-0.735278000
6	0.000000000	-0.792661000	-0.735278000
6	1.271976000	-1.566842000	-0.496948000
1	1.466267000	-2.396323000	-1.193815000
6	1.271976000	1.566842000	-0.496948000
6	2.226822000	-1.187631000	0.397429000
1	1.466267000	2.396323000	-1.193815000
6	2.226822000	1.187631000	0.397429000
6	3.628644000	1.622849000	0.370031000
1	3.862624000	2.684034000	0.213314000

6	0.000000000	-1.226913000	1.394805000
1	0.000000000	-1.227026000	2.472664000
6	0.000000000	-1.226913000	-1.394805000
6	0.000000000	-2.427823000	0.718672000
1	0.000001000	-1.227026000	-2.472664000
6	0.000000000	-2.427823000	-0.718672000
6	0.000000000	-3.684990000	-1.399790000
1	0.000000000	-3.683995000	-2.476960000
6	0.000000000	-4.847821000	-0.713433000
6	0.000000000	-3.684990000	1.399790000
1	-0.000001000	-5.787211000	-1.238925000
1	0.000000000	-3.683995000	2.476960000
6	0.000000000	-4.847821000	0.713433000
1	-0.000001000	-5.787211000	1.238925000

6, HF/6-311++G(*d*,*p*)

6	-2.215770000	-1.185229000	0.411232000
6	-2.215770000	1.185229000	0.411232000
6	-3.630064000	-1.611358000	0.353052000
1	-3.854653000	-2.659247000	0.243467000
6	-3.630064000	1.611358000	0.353052000
6	-4.644913000	-0.743841000	0.384387000
1	-3.854653000	2.659247000	0.243467000
1	-5.634894000	-1.164707000	0.324854000
6	-4.644913000	0.743841000	0.384387000
1	-5.634894000	1.164707000	0.324854000
6	-1.279953000	-1.552102000	-0.449698000
1	-1.464248000	-2.362555000	-1.139133000
6	-1.279953000	1.552102000	-0.449698000
1	-1.464248000	2.362555000	-1.139133000
6	0.000000000	0.781285000	-0.696810000
6	0.000000000	-0.781285000	-0.696810000
6	1.279953000	-1.552102000	-0.449698000
1	1.464248000	-2.362555000	-1.139133000
6	1.279953000	1.552102000	-0.449698000
6	2.215770000	-1.185229000	0.411232000
1	1.464248000	2.362555000	-1.139133000
6	2.215770000	1.185229000	0.411232000
6	3.630064000	1.611358000	0.353052000
1	3.854653000	2.659247000	0.243467000
6	4.644913000	0.743841000	0.384387000
6	3.630064000	-1.611358000	0.353052000
1	5.634894000	1.164707000	0.324854000
1	3.854653000	-2.659247000	0.243467000
6	4.644913000	-0.743841000	0.384387000
1	5.634894000	-1.164707000	0.324854000
6	1.967363000	0.000000000	1.301646000
1	0.961247000	0.000000000	1.678953000
1	2.639096000	0.000000000	2.148835000
6	-1.967363000	0.000000000	1.301646000
1	-2.639096000	0.000000000	2.148835000
1	-0.961247000	0.000000000	1.678953000
6	0.000000000	0.000000000	-1.989795000
1	-0.906972000	0.000000000	-2.570777000
1	0.906972000	0.000000000	-2.570777000

6, KMLYP/6-311+G(*d*,*p*)

6	-2.500725000	-1.335669000	0.468091000
6	-2.500725000	1.335669000	0.468091000
6	-4.080161000	-1.802918000	0.360652000
1	-4.334151000	-2.992747000	0.221834000
6	-4.080161000	1.802918000	0.360652000
6	-5.212254000	-0.831530000	0.374122000
1	-4.334151000	2.992747000	0.221834000
1	-6.338117000	-1.303051000	0.281659000
6	-5.212254000	0.831530000	0.374122000
1	-6.338117000	1.303051000	0.281659000
6	-1.438506000	-1.755055000	-0.465913000
1	-1.627261000	-2.683800000	-1.248686000
6	-1.438506000	1.755055000	-0.465913000
1	-1.627261000	2.683800000	-1.248686000
6	0.000000000	0.891876000	-0.730595000
6	0.000000000	-0.891876000	-0.730595000
6	1.438506000	-1.755055000	-0.465913000
1	1.627261000	-2.683800000	-1.248686000
6	1.438506000	1.755055000	-0.465913000
6	2.500725000	-1.335669000	0.468091000
1	1.627261000	2.683800000	-1.248686000
6	2.500725000	1.335669000	0.468091000
6	4.080161000	1.802918000	0.360652000
1	4.334151000	2.992747000	0.221834000

6	4.673453000	0.737620000	0.480374000
6	3.628644000	-1.622849000	0.370031000
1	5.678109000	1.178846000	0.452203000
1	3.862624000	-2.684034000	0.213314000
6	4.673453000	-0.737620000	0.480374000
1	5.678109000	-1.178846000	0.452203000
6	1.949108000	0.000000000	1.280749000
1	0.906956000	0.000000000	1.619937000
1	2.610372000	0.000000000	2.160749000
6	-1.949108000	0.000000000	1.280749000
1	-2.610372000	0.000000000	2.160749000
1	-0.906956000	0.000000000	1.619937000
6	0.000000000	0.000000000	-2.045925000
1	-0.930883000	0.000000000	-2.625096000
1	0.930883000	0.000000000	-2.625096000

6, BHLYP/6-311+G(*d*,*p*)

6	-2.208877000	-1.181733000	0.403783000
6	-2.208877000	1.181733000	0.403783000
6	-3.609855000	-1.604417000	0.355966000
1	-3.834599000	-2.650919000	0.222707000
6	-3.609855000	1.604417000	0.355966000
6	-4.630405000	-0.735311000	0.417853000
1	-3.834599000	2.650919000	0.222707000
1	-5.620227000	-1.160787000	0.367924000
6	-4.630405000	0.735311000	0.417853000
1	-5.620227000	1.160787000	0.367924000
6	-1.269361000	-1.552558000	-0.459627000
1	-1.452394000	-2.368100000	-1.145016000
6	-1.269361000	1.552558000	-0.459627000
1	-1.452394000	2.368100000	-1.145016000
6	0.000000000	0.782671000	-0.705563000
6	0.000000000	-0.782671000	-0.705563000
6	1.269361000	-1.552558000	-0.459627000
1	1.452394000	-2.368100000	-1.145016000
6	1.269361000	1.552558000	-0.459627000
6	2.208877000	-1.181733000	0.403783000
1	1.452394000	2.368100000	-1.145016000
6	2.208877000	1.181733000	0.403783000
6	3.609855000	1.604417000	0.355966000
1	3.834599000	2.650919000	0.222707000
6	4.630405000	0.735311000	0.417853000
6	3.609855000	-1.604417000	0.355966000
1	5.620227000	1.160787000	0.367924000
1	3.834599000	-2.650919000	0.222707000
6	4.630405000	-0.735311000	0.417853000
1	5.620227000	-1.160787000	0.367924000
6	1.952897000	0.000000000	1.286200000
1	0.939198000	0.000000000	1.650493000
1	2.617361000	0.000000000	2.140591000
6	-1.952897000	0.000000000	1.286200000
1	-2.617361000	0.000000000	2.140591000
1	-0.939198000	0.000000000	1.650493000
6	0.000000000	0.000000000	-1.997882000
1	-0.909113000	0.000000000	-2.576692000
1	0.909113000	0.000000000	-2.576692000

6	5.212254000	0.831530000	0.374122000
6	4.080161000	-1.802918000	0.360652000
1	6.338117000	1.303051000	0.281659000
1	4.334151000	-2.992747000	0.221834000
6	5.212254000	-0.831530000	0.374122000
1	6.338117000	-1.303051000	0.281659000
6	2.244853000	0.000000000	1.469571000
1	1.111943000	0.000000000	1.925824000
1	3.034216000	0.000000000	2.411882000
6	-2.244853000	0.000000000	1.469571000
1	-3.034216000	0.000000000	2.411882000
1	-1.111943000	0.000000000	1.925824000
6	0.000000000	0.000000000	-2.187021000
1	-1.034717000	0.000000000	-2.843417000
1	1.034717000	0.000000000	-2.843417000

6, B3LYP/6-311+G(*d*,*p*)

6	-2.228896000	-1.190871000	0.404915000
6	-2.228896000	1.190871000	0.404915000
6	-3.632567000	-1.612737000	0.353888000
1	-3.859191000	-2.664238000	0.199848000
6	-3.632567000	1.612737000	0.353888000
6	-4.664622000	-0.735631000	0.428927000
1	-3.859191000	2.664238000	0.199848000
1	-5.660865000	-1.166172000	0.378528000
6	-4.664622000	0.735631000	0.428927000
1	-5.660865000	1.166172000	0.378528000
6	-1.273648000	-1.569104000	-0.458705000
1	-1.450991000	-2.396606000	-1.143734000
6	-1.273648000	1.569104000	-0.458705000
1	-1.450991000	2.396606000	-1.143734000
6	0.000000000	0.792436000	-0.706480000
6	0.000000000	-0.792436000	-0.706480000
6	1.273648000	-1.569104000	-0.458705000
1	1.450991000	-2.396606000	-1.143734000
6	1.273648000	1.569104000	-0.458705000
6	2.228896000	-1.190871000	0.404915000
1	1.450991000	2.396606000	-1.143734000
6	2.228896000	1.190871000	0.404915000
6	3.632567000	1.612737000	0.353888000
1	3.859191000	2.664238000	0.199848000
6	4.664622000	0.735631000	0.428927000
6	3.632567000	-1.612737000	0.353888000
1	5.660865000	1.166172000	0.378528000
1	3.859191000	-2.664238000	0.199848000
6	4.664622000	-0.735631000	0.428927000
1	5.660865000	-1.166172000	0.378528000
6	1.971401000	0.000000000	1.290349000
1	0.949215000	0.000000000	1.656054000
1	2.639347000	0.000000000	2.151701000
6	-1.971401000	0.000000000	1.290349000
1	-2.639347000	0.000000000	2.151701000
1	-0.949215000	0.000000000	1.656054000
6	0.000000000	0.000000000	-2.008670000
1	-0.915449000	0.000000000	-2.591350000
1	0.915449000	0.000000000	-2.591350000
